# Direct Synthesis of Bicyclo[1.1.1]pentanes by Sequential C=C, C–C Functionalization Reactions

**DOI:** 10.1021/jacs.5c09039

**Published:** 2025-08-14

**Authors:** Joshua K. Sailer, Duc Ly, Djamaladdin G. Musaev, Huw M. L. Davies

**Affiliations:** † Department of Chemistry, 1371 Emory University, 1515 Dickey Drive, Atlanta, Georgia 30322, United States; ‡ Cherry L. Emerson Center for Scientific Computation, Emory University, 1521 Dickey Drive, Atlanta, Georgia 30322, United States

## Abstract

2-Substituted bicyclo[1.1.1]­pentane carboxylates have been synthesized using two sequential carbene reactions. A dirhodium-catalyzed intramolecular cyclopropanation to form a bicyclo[1.1.0]­butane is followed by a photoinduced formation of a triplet carbene, which undergoes a diradical addition into the strained C–C bond of the bicyclo[1.1.0]­butane. A variety of novel 2-substituted bicyclo[1.1.1]­pentanes have been synthesized in good to moderate yields, offering rapid access to these valuable scaffolds. Additionally, a one-pot, two-reaction sequence was developed in which the bicyclo[1.1.1]­pentanes were afforded starting from two distinct diazo compounds. Computational analysis supports the triplet carbene intermediate addition into the bicyclo[1.1.0]­butane C–C bond. Additionally, computational analysis shows the importance of an aryl substituent to stabilize the diradical intermediate, which is required for the productive formation of the bicyclo[1.1.1]­pentanes.

## Graphical abstract

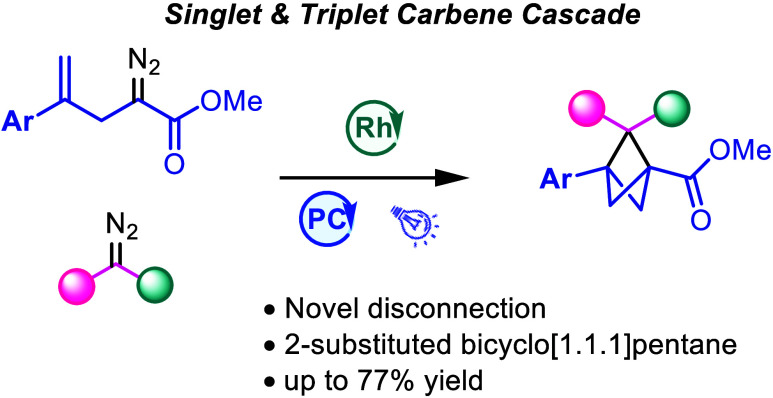

## Introduction

Bicyclo­[1.1.1]­pentane (BCP) has become a highly sought-after scaffold in recent years as a bioisostere for a substituted benzene ring (Scheme 1). In an effort to “escape from flatland”,^1^ *para*-substituted benzene rings have been replaced with 1,3-disubstituted BCPs in some common drug scaffolds in order to increase the pharmacological properties of these drugs.^2^ In particular, increasing more C­(sp^3^) character often improves pharmacological properties of these target molecules; thus, developing facile methods to make BCP building blocks is an important area of research.^3^ 1,3-Disubstituted BCPs are the most common substitution pattern, with many available synthetic routes to access these moieties.^3–8^ However, a much more challenging scaffold to construct is the 1,2-disubstituted or 1,2,3-trisubstituted BCPs. This substitution pattern has been shown to be a possible bioisostere for *ortho*- or *meta*-substituted benzene rings, extending the utility of this valuable motif.^2,7^ Methods have been developed to access the 1,2,3-trisubstituted BCP, such as strain-release addition across [1.1.1]­propellane,^9,10^ intramolecular cyclization,^11,12^ cross-coupling,^13,14^ biocatalysis,^15^ and skeletal editing approaches.^16,17^ However, many of these strategies involve long synthetic sequences or are of limited scope, and consequently, the development of new direct methods to access these compounds is of considerable interest.

**Scheme 1:**
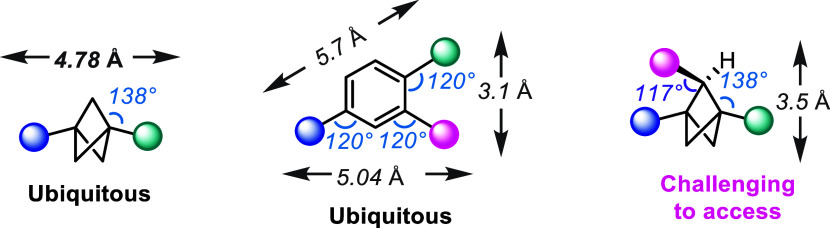
BCPs as Substituted Benzene Bioisosteres

In recent years, a number of C–C functionalization methods of bicyclo[1.1.0]­butanes (BCBs) (**1**) have been developed to access several types of expanded bicyclic systems.^18–21^ Of particular interest has been the ring expansion of BCBs to BCPs as illustrated in Scheme 2A. The addition of dihalocarbenes into BCBs has been known for many years,^22^ but has seen a resurgence from several groups reporting the difluorocarbene addition into the strained C–C bond (Scheme 2A).^23–28^ Recently, the Tan and Lu groups reported an indirect approach involving an imine cycloaddition followed by a nitrogen deletion strategy.^17,29^ When metallocarbene intermediates are used in reaction with a monosubstituted BCB, the reaction fragments to afford the skipped diene products (Scheme 2B).^30,31^ One of the challenges of all of these approaches is that BCBs need to be preformed, which can be problematic due to their instability. In the current study, we report a direct entry to BCPs by two sequential carbene reactions, a rhodium-catalyzed intramolecular cyclopropanation followed by a C–C functionalization of the resulting BCB by a triplet carbene (Scheme 2C). We also report that the process can be carried out in a one-pot process, avoiding the necessity of isolating the relatively unstable BCB intermediate.^26,29^

**Scheme 2:**
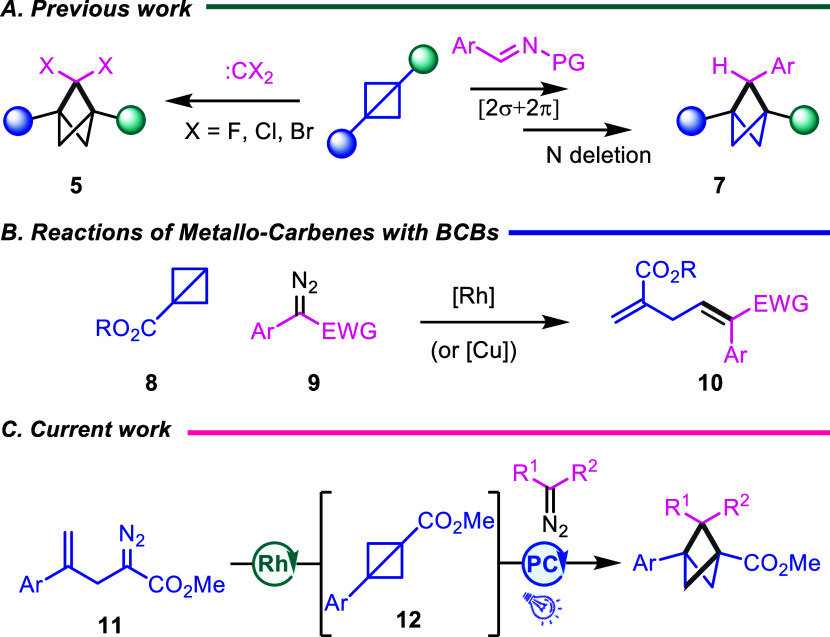
C–C Functionalization of BCBs to Generate Expanded Bicyclic Systems

We have already established the rhodium-catalyzed intramolecular cyclopropanation to generate BCBs,^26,32^ and so the key requirement was to develop a method for C–C functionalization with an appropriate carbene source. Metallocarbene intermediates are not suitable because they prefer the fragmentation pathway with monosubstituted BCBs and are likely to be too sterically demanding for more highly substituted BCBs. Therefore, we turned to carbenes generated by light-induced reactions on diazo compounds, which we popularized a few years ago^33^ and has become a very active field of research.^34–36^ We envisioned that the use of triplet carbenes, recently used in various transformations by Koenigs,^37–39^ Gryko,^40,41^ and others,^42–44^ would be the most promising intermediates to apply to our desired transformation.

## Results and Discussion

The study began by employing ethyl diazoacetate (**13**) with methyl 3-phenylbicyclo[1.1.0]­butane-1-carboxylate (**14**) under 440 nm light irradiation. The optimum conditions were found to be using Ir­(ppy)_3_ (1 mol %) in dichloromethane at −65 °C at a concentration of 0.05 M (Table 1, entry 1). In the small-scale optimization study (0.10 mmol), BCP product **15** was obtained in 50% yield (by NMR, entry 1). The initial hit was obtained in a room-temperature reaction, forming **15** in 13% yield (entry 2). An improvement in the yield was observed on cooling to 0 and −40 °C (entries 3 and 4), which led to the exploration of even lower temperatures. A reaction temperature of −65 °C was eventually selected as optimum because a slightly lower yield was obtained when was conducted at −78 °C (entry 5). Increasing the photocatalyst loading to either 2.5 or 5.0 mol % proved to be suboptimal (entries 6 and 7). Next, several known triplet sensitizers were screened (entries 8–11), but none performed as well as Ir­(ppy)_3_. Dichloromethane was seen to be the optimum solvent, with only 6% of **15** being formed when the reaction was run in chloroform (entry 12) and none observed when the reaction was run in THF, diethyl ether, or hexanes (entry 13). Varying the equivalents of EDA or increasing the concentration resulted in decreased yields compared with the standard conditions (entries 14 and 15). Having established that the conditions in entry 1 were optimum, the reaction was repeated at a 0.2 mmol scale in order to record an isolated yield, and under the larger scale conditions, the yield of **15** improved to 66%.

**Table 1:**
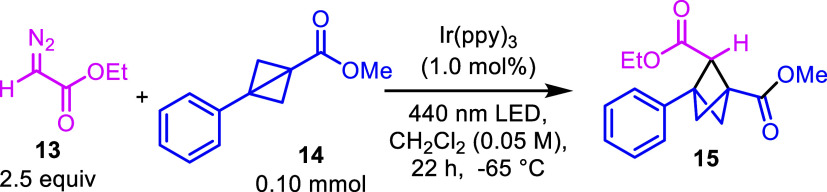
Optimization of Reaction Conditions

entry	deviations from above	yield (%)^a^
1	none	50 (66)^b^
2	25 °C	13
3	0 °C	36
4	–40 °C	36
5	–78 °C	45
6	lr(ppy)_3_ (2.5 mol %)	40
7	lr(ppy)_3_ (5.0 mol %)	21
8	[lr(ppy)_2_(dtbbpy)]PF_6_ as PC	22
9	[lr[dF(CF_3_)ppy]_2_(dtbpy)]PF_6_ as PC	34
10	4-CzIPN as PC	35
11	TX as PC	<10
12	CHCl_3_ as solvent	6
13	THF, diethyl ether, or hexane as solvent	0
14	5 equiv of EDA	25
15	CH_2_Cl_2_ (0.10 M)	36
Control Reactions		
16	no photocatalyst or no light	0
17	2.5 equiv of TEMPO	0
18	Oxygen atmosphere	5
19	2.5 equiv of perylene	14
20	370 nm light, as PC	7

a NMR yield using 1,3,5-trimethoxybenzene as internal standard.

b Reaction ran at 0.20 mmol, isolated yield. Triplet energies of catalysts:^45,46^ Ir­(ppy)_3_ E*_T_ = 59.6 kcal/mol, [Ir­(ppy)_2_(dtbbpy)]­PF_6_ E*_T_ = 50.0 kcal/mol, [Ir­[dF­(CF_3_)­ppy]_2_(dtbpy)]­PF_6_ E*_T_ = 66.9 kcal/mol, 4-CzIPN E*_T_ = 59.7 kcal/mol, TX E*_T_ = 65.5 kcal/mol.

Control experiments were carried out to confirm that the reaction is likely to proceed through triplet carbenes (entries 15–19). When the reaction was conducted in the absence of 440 nm light or photocatalyst, no product was obtained (entry 16). When TEMPO, a radical trap, was added, none of BCP was observed, and the HR-MS analysis of the crude reaction mixture indicated the presence of TEMPO diadducts (entry 17). When triplet quenchers were added, the yield dropped considerably (5% with oxygen and 14% with perylene; entries 18 and 19). Finally, when a photochemical reaction was conducted with 370 nm light in the absence of a photocatalyst, **15** was obtained in low yield (7%, entry 20), indicating an energy transfer mechanism. Overall, the results are consistent with the reaction of the triplet diradical with the central bond of BCB to form a diradical that then closes to form BCP.

With the optimized conditions in hand, the scope of this transformation was evaluated. A variety of diazo compounds were amenable to this methodology (Table 2). Alkyl diazoester compounds were found to work nicely, with both methyl 2-diazopropionate and methyl 2-diazobutanoate yielding products **16** and **17** in 59% and 40% yield, respectively. These results were somewhat surprising due to the fact that alkyl diazoesters are highly prone to undergo a 1,2-shift from the corresponding singlet carbene resulting in the formation of α, β-unsaturated esters.^47–50^ The propensity to undergo the hydride shift increases with decreasing bond strength of the β C–H bond, thus it would be expected for the methine carbene derivative to be the most susceptible to this side reaction.^51^ Indeed, when methyl 2-diazo-3-methylbutanoate was subjected to the reaction conditions, no BCP was detected. Benzyl esters were compatible, as seen with the formation of **18** and **19** in 48% and 77% yield, respectively. The formation of **19** was run on a 1.0 mmol scale, illustrating the scalability of these reactions. When 2,2,2-trichloroethyl 2-diazoacetate was used as the carbene precursor under the standard conditions, only a trace product was observed. We hypothesized that using a photocatalyst with a higher triplet energy would be more effective. Gratifyingly, using thioxanthone (TX, E*_T_ = 65.5 kcal/mol)^45^ as the photocatalyst under 390 nm irradiation gave product **20** in 39% yield, in which the two carboxylic acids have orthogonal ester protecting groups. It was found that four other diazo compounds needed the TX photocatalyst to give appreciable yields. The phosphonate and trifluoromethyl derivatives **21** and **22** were formed in 24% and 52% yields, respectively. These compounds provide interesting scaffolds for further drug discovery, allowing access to synthetically challenging substituents on the BCP core. Finally, the cyclic γ-lactone and valerolactone-derived diazo compounds gave the spiro-BCP **23** and **24** in 41% and 61% yields, respectively. Further confirmation of the structural assignment of **24** was unambiguously obtained by X-ray crystallographic analysis. The influence of the aryl substituent was then examined in the reaction of the bicyclo[1.1.0]­butanes with methyl 2-diazopropionate as the carbene precursor. A naphthyl group on BCB was shown to be compatible, generating BCP **25** in 52% yield. The methodology was effective with aryl BCBs with *para*-bromo, -methyl, and -trifluoromethyl substituents forming the desired products in 67%, 77%, and 39% yields, respectively (**26–28**). A 3,4-dichloro substitution pattern was shown to be amenable, with **29** being formed in 58% yield. Finally, the steric environment around the phenyl ring seems to have a minimal effect on the reaction, as the *ortho-*methyl substituent resulted in a 71% yield of the desired product **30**. The triplet carbene ring expansion of the arylbicyclobutanes has limitations with regard to carbene structure (see Supporting Information for details). The reaction with tertiary alkyldiazoacetates such as isopropyldiazoacetate is very low yielding, and no product was obtained with a diazoketone or diazoacetoacetate. The use of carbene precursors lacking an acceptor group also failed to give ring-expanded products.

**Table 2:**
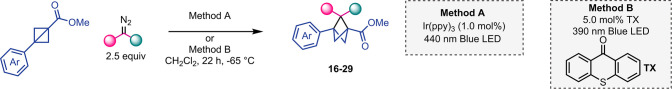

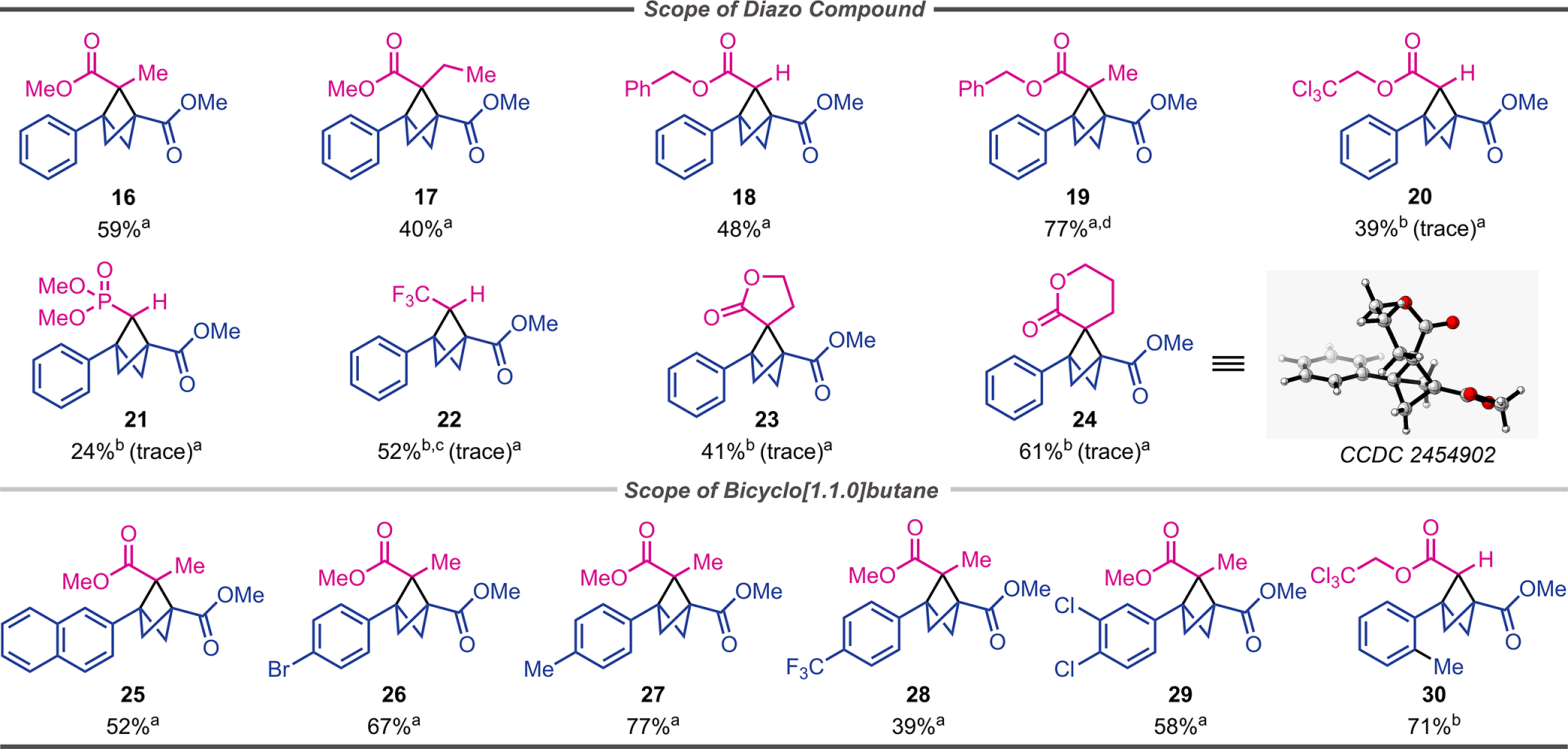
Scope of the Reaction with Triplet Carbene Addition^e^

a Prepared using Method A.

b Prepared using Method B.

c 5.0 equiv of diazo compound used.

d 1.0 mmol scale.

e Reaction conditions: 0.20 mmol of BCB, 2.5 equiv of diazo compound in CH_2_Cl_2_.

In order to evaluate the importance of the aryl substituent in these ring expansions, monosubstituted BCB **31** was prepared and subjected to the reaction conditions (Scheme 3). Interestingly, the major product from the reaction with **31** was cyclopropane **33**, presumably generated from diene **32**, through the opening of both rings in BCB, with the remaining mass balance being recovered BCB.

**Scheme 3:**
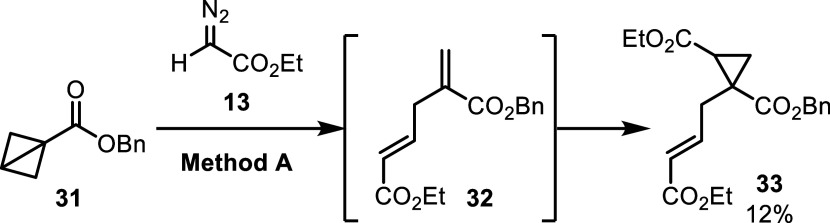
Reaction with Monosubstituted BCB

Next, the practicality of this methodology was tested by showcasing a one-pot sequential reaction procedure (Table 3). The dirhodium-catalyzed cyclopropanation to generate BCB proceeds in essentially quantitative yield with a high tolerance toward additives and impurities needing only 0.01 mol % catalyst loading. Previously, it has been shown that the optimum dirhodium catalyst for the cyclopropanation is Rh_2_(Oct)_4_.^26,33^ We hypothesized that the rhodium-catalyzed cyclopropanation would be tolerant of both the iridium and TX photocatalyst, allowing for the rhodium and photocatalyst to be added at the beginning of the reaction sequence. Then, following completion of the BCB formation, pyridine could be added to deactivate the dirhodium catalyst, followed by the addition of the second diazo compound and blue light irradiation at −65 °C to afford the desired BCP product. This protocol was applied to nine diazo compounds as illustrated in Table 3. Remarkably, in several of the cases (substrates **16**, **18**, **20**, and **24**), the one-pot process gives an improved overall yield over the two isolated steps, which may be due to the instability of the BCB starting material over time. Additionally, when the reaction is done in one pot, the carbene cyclization step extrudes dinitrogen, further degassing the solvent from any dissolved oxygen in the system, which also could contribute to the increased yields. In order to evaluate scalability, one of the reactions was conducted on a 2 mmol scale, resulting in the formation of **20** in 54% yield. Additionally, the one-pot sequence was used with two new diazo compounds, with 2-diazoacetonitrile and ethyl 2-diazo-2-(trimethylsilyl)­acetate yielding **34** and **35** in 58% and 13% yields, respectively. The one-pot process is particularly appealing, as it offers a novel disconnection strategy and a new mode of cross-coupling between two diazo compounds.

**Table 3:**
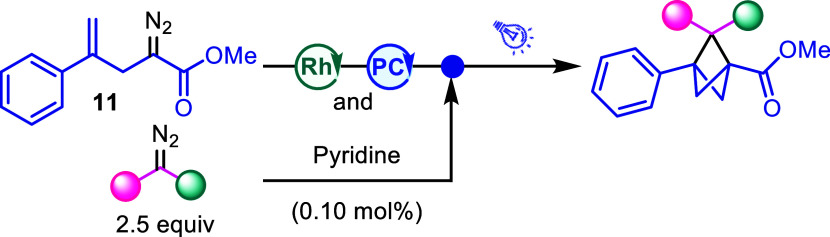

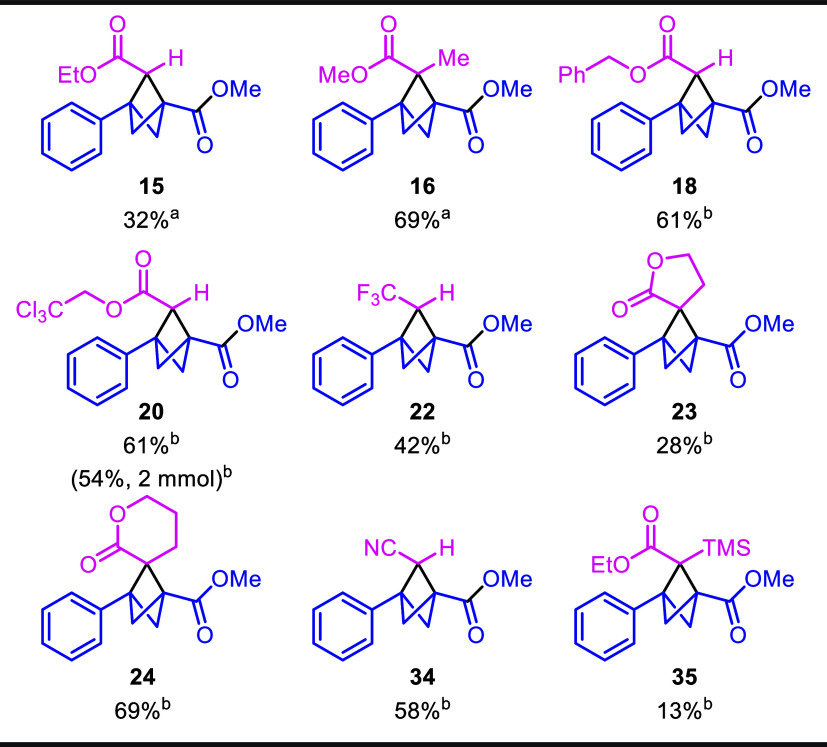
BCP Generation by a One-Pot Procedure^c^

a Ir­(ppy)_3_ (1.0 mol %) was used as photocatalyst.

b TX (5.0 mol %) was used as photocatalyst.

c Reaction conditions: diazo **11** (0.2 mmol), Rh_2_(Oct)_4_ (0.01 mol %), and photocatalyst, then pyridine­(0.1 mol%) and diazo (2.5 equiv) in CH_2_Cl_2_.

To gain a better understanding of these results and the mechanism of the C–C activation reaction, a computational analysis by density functional theory (DFT) was performed. The calculated energy profiles at [(U)­ωB97X-D^52^ + CPCM­(CH_2_Cl_2_)][Bibr ref53]/def2TZVPP^54^//[(U)­ωB97X-D + CPCM­(CH_2_Cl_2_)]/def2SVP level of theory for the C–C activation reaction of monosubstituted BCB (**36**) and 1,3-disubstituted BCB (**14**) are shown in Figure 1. Herein, we are hypothesizing that at first, the diazo compound **13** excites to its triplet state ^**3**^ **13** by the photocatalyst via the energy transfer pathway, which then undergoes a nitrogen extrusion to generate the reactive triplet carbene ^**3**^ **I** species^36,55–57^ (see Figure S2). The addition of ^**3**^ **I** to BCB **14** is favored to happen on the ester side of the C–C bond by 1.0 kcal/mol, forming triplet diradical intermediate ^**3**^ **II**, presumably due to the formation of a more stable radical center. Intermediate ^**3**^ **II** undergoes intersystem crossing to form singlet diradical intermediate ^**1**^ **II**. The radical recombination was found to be a critical step for the successful formation of BCP **15** due to the competitive fragmentation pathway to form the skipped diene ^**1**^ **IV** which is exclusively favored for singlet carbene (see Figure S3).^26^ Fortunately, the formation of BCP **15** is 6.4 kcal/mol more favored than the deleterious fragmentation pathway. On the other hand, the C–C activation of monosubstituted BCB **36** is almost identical to that of BCB **14**, except for the final step. In this case, the fragmentation of intermediate ^**1**^ **V** to skipped diene ^**1**^ **VII** was more favorable than the formation of BCP product ^**1**^ **VI** by 2.6 kcal/mol. A possible reason for the change in the favored pathway is that the radical recombination in ^**1**^ **V** to form ^**1**^ **VI** involves a reaction between two electrophilic radicals, which would be expected to be less favorable. This result is in good agreement with the experimental observation (Scheme 3).

**Figure 1:**
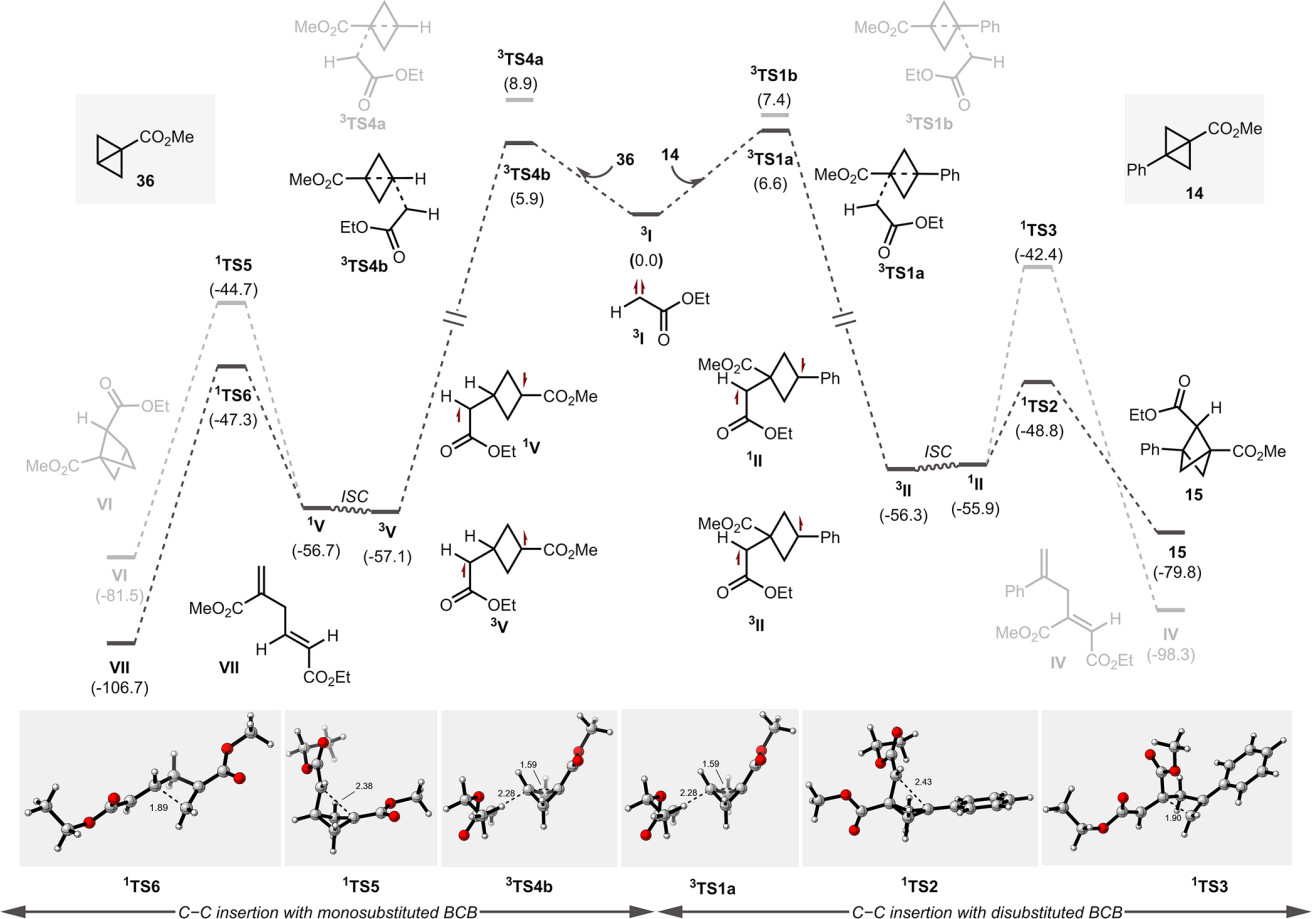
Computational study on C–C activation of monosubstituted bicyclobutane and disubstituted bicyclobutane with triplet carbene. The DFT calculation was the [(U)­ωB97X-D + CPCM­(CH_2_Cl_2_)]/def2TZVPP//[(U)­ωB97X-D + CPCM­(CH_2_Cl_2_)]/def2SVP level at the reaction conditions. The reported energies are free energies in kcal/mol.

## Conclusions

In summary, a facile synthesis of 2-substituted bicyclo[1.1.1]­pentanes is disclosed using a photosensitized carbene addition into a bicyclo[1.1.0]­butane.^58^ This methodology is generalizable to a variety of diazo carbene precursors, giving rise to novel BCP scaffolds that will generate interest in drug development. Additionally, it was shown that switching the mode of carbene reactivity from singlet to triplet opened new avenues of reactivity, which we believe will help drive the field of photosensitized carbene methodology forward. A one-pot sequential reaction setup was shown to be operational, starting from dirhodium-catalyzed BCB cyclization followed by photocatalyzed BCP formation. Finally, computational analysis was performed to validate the triplet carbene pathway, showing that the triplet carbene is the operational intermediate for this novel reaction. This methodology enables novel scaffolds to be explored for drug discovery and hopefully inspires further work in the area of diazo photosensitization.

## Supplementary Material



## Data Availability

The data underlying this study are available in the published article and its online Supporting Information.

## Notes

Complete experimental procedures, materials, computational details and findings, compound characterizations, and Cartesian coordinates of all calculated systems (PDF)
