# Supplementary material for: Direct Synthesis of Bicyclo[1.1.1]pentanes by Sequential C=C, C–C Functionalization Reactions
Source: J Am Chem Soc. 2025 Aug 14;147(34):31034–41. doi: 10.1021/jacs.5c09039 (PMC12395405; doi:10.1021/jacs.5c09039)
Supplement: Supplementary file 1 [file ja5c09039_si_001.pdf]

Direct Synthesis of Bicyclo[1.1.1]pentanes by Sequential C=C, C–C Functionalization Reactions

Joshua K. Sailer,[1‡] Duc Ly,[1‡], Djamaladdin G. Musaev[1,2], Huw M. L. Davies[1*]

[1]Department of Chemistry, Emory University, 1515 Dickey Drive, Atlanta, Georgia 30322, United States.

[2]Cherry L. Emerson Center for Scientific Computation, Emory University, 1521 Dickey Drive, Atlanta, Georgia, 30322, United States.

*Corresponding authors. Email: hmdavie@emory.edu (H.M.L.D.)

‡These authors contributed equally

Supporting Information

Complete experimental procedures, materials, computational details, and compound characterizations

Table of Contents

1. General Considerations....... S2

2. Low temperature irradiation setup....... S3

3. Known Compounds:....... S4

4. General Procedures....... S5

5. C-C activation reaction....... S6

6. One-pot procedure....... S16

7. Copies of NMR of Novel Compounds....... S22

8. DFT computational study....... S44

9. X-ray determination....... S77

10. References....... S93

1. General Considerations

All experiments were carried out in flame-dried or oven-dried glassware under argon atmosphere unless otherwise stated. Flash column chromatography was performed on silica gel. Unless otherwise noted, all other reagents were obtained from commercial sources (Sigma Aldrich, Fisher, TCI Chemicals, AK Scientific, Combi Blocks, Oakwood Chemicals, Ambeed) and used as received without purification. ^{1}H, ^{13}C, and ^{19}F NMR spectra were recorded at either 400 MHz (^{13}C at 100 MHz) on Bruker 400 spectrometer or 600 MHz (^{13}C at 151 MHz) on INOVA 600 or Bruker 600 spectrometer. NMR spectra were run in solutions of deuterated chloroform ($CDCl_3$) with residual chloroform taken as an internal standard (7.26 ppm for ^{1}H, and 77.16 ppm for ^{13}C), and were reported in parts per million (ppm). The abbreviations for multiplicity are as follows: s = singlet, d = doublet, t = triplet, q = quartet, p = pentet, m = multiplet, dd = doublet of doublet, etc. Coupling constants (J values) are obtained from the spectra. Thin layer chromatography was performed on aluminum-back silica gel plates with UV light and cerium aluminum molybdate (CAM) stain to visualize. Mass spectra were taken on a Thermo Finnigan LTQ-FTMS spectrometer with APCI or ESI.

2. Low temperature irradiation setup

The screw-cap photoreaction vials were placed in a crystallization dish in an acetone bath, completely submerging the vial in acetone up to the solvent line. The acetone bath was cooled using Thermo/Neslab CB80 Cryocool. The reactions were irradiated using either a 440 nm Kessel lamp at 100% intensity or 390 nm Kessel lamp at 100% intensity. The light source was ~13 cm away from the vials. The temperature of the acetone bath was verified using a low temperature alcohol thermometer.

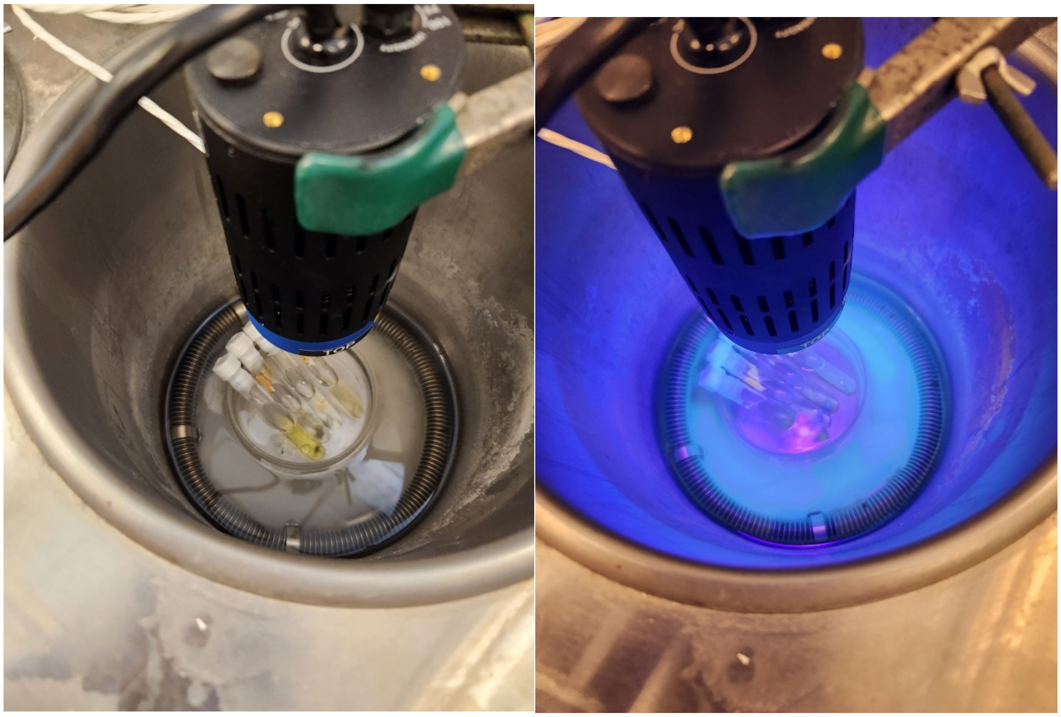

3. Known Compounds:

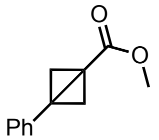
14

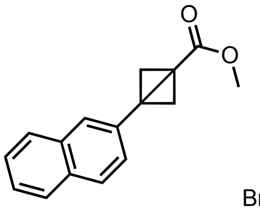
S1

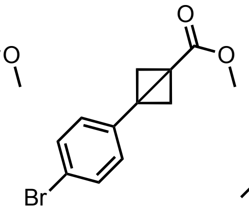
S2

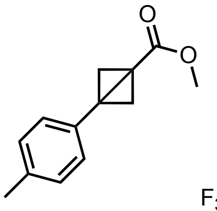
S3

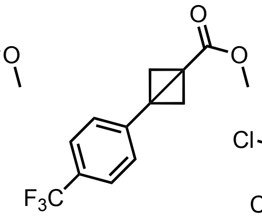
S4

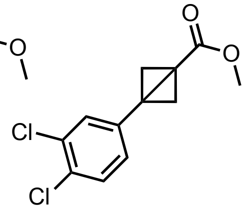
S5

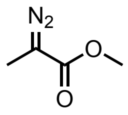
S6

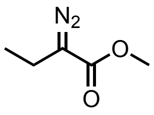
S7

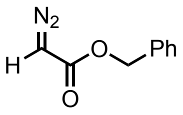
S8

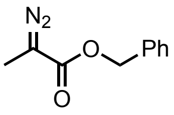
S9

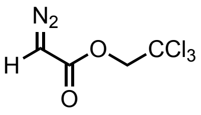
S10

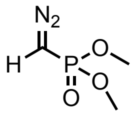
S11

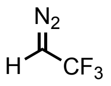
S12

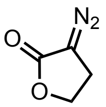
S13

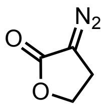
S14

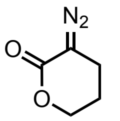
S15

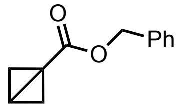
30

Compound **8, S1-S5** were synthesized according to known methods and spectra matched the literature reported spectra.[1]

Compounds **S6** and **S7** were synthesized according to known methods and spectra matched the literature reported spectra.[2]

Compounds **S8**,[3] **S9**, **S10**,[4] **S11**,[5] **S12**,[6] **S13**[7]**, S14-15**[8] and **30**[9] were synthesized according to known methods and spectra matched the literature reported spectra.

4. General Procedures

General Procedure A

To an oven dried photoreaction tube under inert atmosphere was added Ir(ppy)$_3$ (1.0 mol%, 1.31 mg) and the bicyclo[1.0]butane (0.20 mmol, 1.0 equiv). This was purged and backfilled three times with nitrogen. Then, 2 mL of DCM (degassed for 20 minutes using an argon balloon) as added to the reaction vessel. The diazo was weighed out into a separate vial and was purged with nitrogen, followed by the addition of 2 mL of DCM. Then, the diazo solution was added to the reaction vessel, the septum was sealed with parafilm, and the vessel was placed in a -65 °C acetone bath using the constant chiller. The reaction was irradiated with 440 nm Kessel lamp at 100% intensity for 22 h. At this time the reaction solution was concentrated and analyzed for crude NMR before column chromatography to afford the desired product.

General Procedure B

To an oven dried photoreaction tube under inert atmosphere was added thioxanthone (5.0 mol%, 2.12 mg) and the bicyclo[1.0]butane (0.20 mmol, 1.0 equiv). This was purged and backfilled three times with nitrogen. Then, 2 mL of DCM (degassed for 20 minutes using an argon balloon) as added to the reaction vessel. The diazo (0.5-1.0 mmol, 2.5-5.0 equiv) was weighed out into a separate vial and was purged with nitrogen, followed by the addition of 2 mL of DCM. Then, the diazo solution was added to the reaction vessel, the septum was sealed with parafilm, and the vessel was placed in a -65 °C acetone bath using the constant chiller. The reaction was irradiated with 390 nm Kessel lamp at 100% intensity for 22 h. At this time the reaction solution was concentrated and analyzed for crude NMR before column chromatography to afford the desired product.

5. C-C activation reaction

2-ethyl 1-methyl 3-phenylbicyclo[1.1.1]pentane-1,2-dicarboxylate (15)

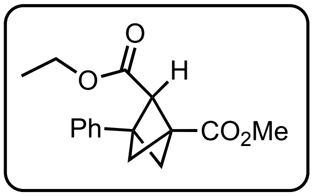

General procedure A was used for the reaction of methyl 3-phenylbicyclo[1.1.0]butane-1-carboxylate (0.20 mmol, 37.6 mg, 1 equiv) with ethyl 2-diazoacetate (0.50 mmol, 55.6 µL 83% wt in toluene, 2.5 equiv) using $Ir(ppy)_3$ (1.0 mol%, 1.31 mg) as catalyst and 440 nm Kessil lamp. The reaction was purified using column chromatography (0-12% diethyl ether/hexanes gradient) affording a clear, colorless oil (28.8 mg, 55%).

^{1}H NMR (400 MHz, $CDCl_3$) δ 7.39 – 7.24 (m, 5H), 4.17 (q, J = 7.1 Hz, 2H), 3.77 (s, 3H), 3.37 (d, J = 7.0 Hz, 1H), 3.08 (dd, J = 9.8, 2.8 Hz, 1H), 2.37 (dd, J = 7.0, 2.8 Hz, 1H), 2.31 (dd, J = 9.8, 1.9 Hz, 1H), 2.25 (d, J = 1.8 Hz, 1H), 1.23 (t, J = 7.2 Hz, 3H).

^{13}C NMR (101 MHz, $CDCl_3$) δ 169.4, 169.1, 137.1, 128.3, 127.5, 126.6, 64.2, 60.5, 52.7, 52.0, 48.8, 46.3, 40.3, 14.2**.**

HRMS (+pAPCI): Calcd for $C_{16}H_{19}O_4$ [M+H] 275.1278, found 275.1280.

dimethyl 2-methyl-3-phenylbicyclo[1.1.1]pentane-1,2-dicarboxylate (16)

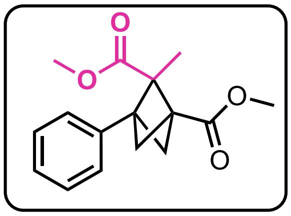

General procedure A was used for the reaction of methyl 3-phenylbicyclo[1.1.0]butane-1-carboxylate (0.20 mmol, 37.6 mg, 1 equiv) with methyl 2-diazopropanoate (0.50 mmol, 57.1 mg, 2.5 equiv) using $Ir(ppy)_3$ (1.0 mol%, 1.31 mg) as catalyst and 440 nm Kessil lamp. The reaction was purified using column chromatography (0-12% diethyl ether/hexanes gradient) affording a clear, colorless oil (32.1 mg, 59%).

^{1}H NMR (400 MHz, $CDCl_3$) δ 7.40 – 7.24 (m, 5H), 3.75 (s, 3H), 3.68 (s, 3H), 2.84 (dd, J = 10.3, 3.0 Hz, 1H), 2.46 (dd, J = 10.3, 3.5 Hz, 1H), 2.24 (d, J = 3.5 Hz, 1H), 2.05 (d, J = 3.0 Hz, 1H), 1.58 (s, 3H).

^{13}C NMR (101 MHz, $CDCl_3$) δ 174.5, 169.1, 136.2, 128.2, 127.4, 127.2, 69.9, 51.8, 51.7, 49.1, 48.7, 47.2, 42.9, 13.1.

HRMS (+pAPCI): Calcd for $C_{16}H_{19}O_4$ [M+H] 275.1278, found 275.1275.

dimethyl 2-ethyl-3-phenylbicyclo[1.1.1]pentane-1,2-dicarboxylate (17)

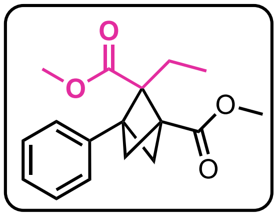

General procedure A was used for the reaction of methyl 3-phenylbicyclo[1.1.0]butane-1-carboxylate (0.20 mmol, 37.6 mg, 1 equiv) with methyl 2-diazobutanoate (0.50 mmol, 64.1 mg, 2.5 equiv) using Ir(ppy)$_3$ (1.0 mol%, 1.31 mg) as catalyst and 440 nm Kessil lamp. The reaction was purified using column chromatography (0-12% diethyl ether/hexanes gradient) affording a clear, colorless oil (21.8 mg, 40%).

^{1}H NMR (400 MHz, CDCl$_3$) δ 7.36 – 7.26 (m, 4H), 3.75 (s, 3H), 3.70 (s, 3H), 2.91 (dd, J = 10.3, 3.1 Hz, 1H), 2.48 (dd, J = 10.3, 3.5 Hz, 1H), 2.21 – 2.09 (m, 2H), 2.09 – 1.94 (m, 2H), 0.86 (t, J = 7.6 Hz, 3H).

^{13}C NMR (101 MHz, CDCl$_3$) δ 173.6, 169.2, 136.6, 128.1, 127.4, 127.3, 75.7, 51.8, 51.4, 49.3, 48.6, 46.4, 43.0, 20.6, 10.3.

HRMS (+pAPCI): Calcd for $C_{17}H_{21}O_4$ [M+H] 289.1434, found 289.1432.

2-benzyl 1-methyl 3-phenylbicyclo[1.1.1]pentane-1,2-dicarboxylate (18)

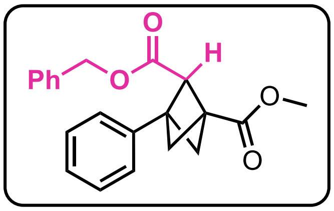

General procedure A was used for the reaction of methyl 3-phenylbicyclo[1.1.0]butane-1-carboxylate (0.20 mmol, 37.6 mg, 1 equiv) with benzyl 2-diazoacetate (0.50 mmol, 88.1 mg, 2.5 equiv) using Ir(ppy)$_3$ (1.0 mol%, 1.31 mg) as catalyst and 440 nm Kessil lamp. The reaction was purified using column chromatography (0-12% diethyl ether/hexanes gradient) affording a clear, colorless oil (32.1 mg, 48%).

^{1}H NMR (400 MHz, CDCl$_3$) δ 7.39 – 7.36 (m, 1H), 7.35 – 7.31 (m, 5H), 7.30 – 7.26 (m, 2H), 7.26 – 7.21 (m, 2H), 5.19 (d, J = 12.5 Hz, 1H), 5.12 (d, J = 12.5 Hz, 1H), 3.72 (s, 3H), 3.45 (d, J = 7.0 Hz, 1H), 3.08 (dd, J = 9.8, 2.9 Hz, 1H), 2.38 (dd, J = 7.1, 2.9 Hz, 1H), 2.33 (dd, J = 9.7, 1.9 Hz, 1H), 2.26 (d, J = 1.9 Hz, 1H).

^{13}C NMR (101 MHz, CDCl$_3$) δ 169.2, 169.0, 137.0, 135.8, 128.5 , 128.3, 128.1, 128.0, 127.5, 126.7, 66.2, 64.1, 52.8, 52.0, 48.7, 40.3.

HRMS (+pAPCI): Calcd for $C_{21}H_{21}O_4$ [M+H] 337.1434, found 337.1434.

2-benzyl 1-methyl 2-methyl-3-phenylbicyclo[1.1.1]pentane-1,2-dicarboxylate (19)

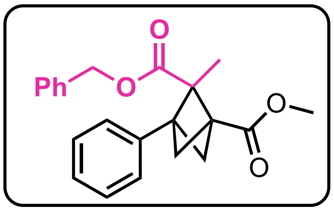

General procedure A was used for the reaction of methyl 3-phenylbicyclo[1.1.0]butane-1-carboxylate (0.20 mmol, 37.6 mg, 1 equiv) with benzyl 2-diazopropanoate (0.50 mmol, 95.1 mg, 2.5 equiv) using Ir(ppy)$_3$ (1.0 mol%, 1.31 mg) as catalyst and 440 nm Kessil lamp. The reaction was purified using column chromatography (0-12% diethyl ether/hexanes gradient) affording a clear, colorless oil (38.6 mg, 55%).

^{1}H NMR (400 MHz, CDCl$_3$) δ 7.36 – 7.32 (m, 4H), 7.32 – 7.29 (m, 2H), 7.29 – 7.23 (m, 4H), 5.20 (d, J = 12.5 Hz, 1H), 5.12 (d, J = 12.5 Hz, 1H), 3.69 (s, 3H), 2.88 (dd, J = 10.3, 3.0 Hz, 1H), 2.50 (dd, J = 10.2, 3.5 Hz, 1H), 2.26 (d, J = 3.5 Hz, 1H), 2.08 (d, J = 3.0 Hz, 1H), 1.64 (s, 3H).

^{13}C NMR (101 MHz, CDCl$_3$) δ 173.8, 169.1, 136.2, 135.8, 128.5, 128.2, 128.1, 128.0, 127.4, 127.3, 69.9, 66.1, 51.7, 49.0, 48.8, 47.2, 42.9, 13.1.

HRMS (+pAPCI): Calcd for $C_{22}H_{23}O_4$ [M+H] 351.1591, found 351.1589.

1-methyl 2-(2,2,2-trichloroethyl) 3-phenylbicyclo[1.1.1]pentane-1,2-dicarboxylate (20)

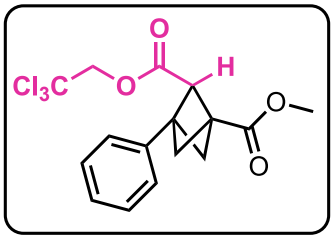

General procedure B was used for the reaction of methyl 3-phenylbicyclo[1.1.0]butane-1-carboxylate (0.20 mmol, 37.6 mg, 1 equiv) with 2,2,2-trichloroethyl 2-diazoacetate (0.50 mmol, 109 mg, 2.5 equiv) using TX (5.0 mol%, 2.12 mg) as catalyst and 390 nm Kessil lamp. The reaction was purified using

column chromatography (0-12% diethyl ether/hexanes gradient) affording a clear, colorless oil (29.6 mg, 39%).

^{1}H NMR (400 MHz, CDCl$_3$) δ 7.36 – 7.26 (m, 5H), 4.73 (d, J = 2.9 Hz, 2H), 3.75 (s, 3H), 3.52 (d, J = 7.0 Hz, 1H), 3.07 (dd, J = 9.8, 3.1 Hz, 1H), 2.42 (dd, J = 7.0, 3.1 Hz, 1H), 2.34 (dd, J = 9.8, 2.0 Hz, 1H), 2.27 (d, J = 2.0 Hz, 1H).

^{13}C NMR (101 MHz, CDCl3) δ 168.7, 167.8, 136.5, 128.4, 127.7, 126.7, 94.6, 74.0, 63.4, 53.3, 52.1, 48.7, 46.7, 40.4.

HRMS (+pAPCI): Calcd for $C_{16}H_{16}O_4{}^{35}Cl_3$ [M+H] 377.0109, found 377.0109.

methyl 2-(dimethoxyphosphoryl)-3-phenylbicyclo[1.1.1]pentane-1-carboxylate (21)

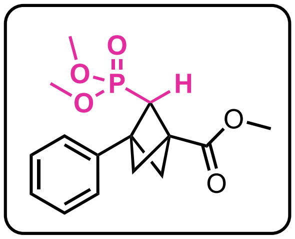

General procedure B was used for the reaction of methyl 3-phenylbicyclo[1.1.0]butane-1-carboxylate (0.20 mmol, 37.6 mg, 1 equiv) with dimethyl (diazomethyl)phosphonate (0.50 mmol, 75.0 mg, 2.5 equiv) using TX (5.0 mol%, 2.12 mg) as catalyst and 390 nm Kessil lamp. The reaction was purified using column chromatography (50-75% ethyl aceate/hexanes gradient) affording a clear, colorless oil (14.8 mg, 24%)

^{1}H NMR (400 MHz, CDCl$_3$) δ 7.40 – 7.26 (m, 5H), 3.75 (s, 3H), 3.68 (d, J = 10.9 Hz, 3H), 3.56 (dd, J = 9.9, 2.8 Hz, 1H), 3.45 (d, J = 10.9 Hz, 3H), 2.94 (t, J = 7.1 Hz, 1H), 2.39 (dd, J = 9.9, 1.7 Hz, 1H), 2.35 (dd, J = 7.5, 2.7 Hz, 1H), 2.32 – 2.20 (m, 1H).

^{13}C NMR (101 MHz, CDCl$_3$) δ 168.9, 137.2, 128.3, 127.6, 126.6, 60.6, 59.1, 56.5, 56.2, 52.5 (d, J = 6.5 Hz), 52.1, 52.0, 49.2 (d, J = 6.9 Hz), 46.1 (d, J = 3.4 Hz), 40.4 (d, J = 3.6 Hz).

^{31}P NMR (162 MHz, CDCl$_3$) δ 24.73 (dddd, J = 28.8, 21.9, 18.0, 11.0 Hz).

HRMS (+pAPCI): Calcd for $C_{15}H_{20}O_5P$ [M+H] 311.1043, found 311.1038.

methyl 3-phenyl-2-(trifluoromethyl)bicyclo[1.1.1]pentane-1-carboxylate (22)

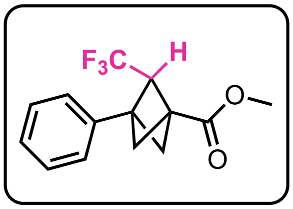

General procedure B was used for the reaction of methyl 3-phenylbicyclo[1.1.0]butane-1-carboxylate (0.20 mmol, 37.6 mg, 1 equiv) with 2-diazo-1,1,1-trifluoroethane (1.0 mmol, 1.37 mL of 0.73 M solution, 5 equiv) using TX (5.0 mol%, 2.12 mg) as catalyst and 390 nm Kessil lamp. The reaction was purified using column chromatography (0-12% diethylether/hexanes gradient) affording a clear, colorless oil (28.1 mg, 52%).

^{1}H NMR (400 MHz, $CDCl_3$) δ 7.40 – 7.27 (m, 3H), 7.26 – 7.19 (m, 2H), 3.76 (s, 3H), 3.24 (qd, J = 9.1, 6.6 Hz, 1H), 3.16 (ddd, J = 10.1, 3.5, 1.6 Hz, 1H), 2.38 (ddd, J = 7.1, 3.5, 1.8 Hz, 1H), 2.26 (dd, J = 10.0, 2.0 Hz, 1H), 2.21 (d, J = 1.9 Hz, 1H).

^{13}C NMR (101 MHz, $CDCl_3$) δ 168.3, 136.1, 128.5, 127.8, 126.4, 124.7 (q, J = 282.1 Hz), 63.2 (q, J = 29.8 Hz), 53.8, 52.2, 47.5 (q, J = 2.1 Hz), 45.7 (q, J = 2.8 Hz), 40.0 (q, J = 3.2 Hz), 35.8.

^{19}F NMR (376 MHz, $CDCl_3$) δ -59.37 (d, J = 9.3 Hz).

HRMS (+pAPCI): Calcd for $C_{14}H_{14}O_2F_3$ [M+H] 271.0940, found 271.0941.

methyl-2'-oxo-3-phenyldihydro-2'H-spiro[bicyclo[1.1.1]pentane-2,3'-furan]-1-carboxylate (23)

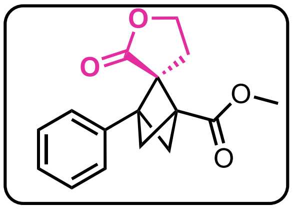

General procedure B was used for the reaction of methyl 3-phenylbicyclo[1.1.0]butane-1-carboxylate (0.20 mmol, 37.6 mg, 1 equiv) with 3-diazodihydrofuran-2(3H)-one (0.50 mmol, 56.0 mg, 2.5 equiv) using TX (5.0 mol%, 2.12 mg) as catalyst and 390 nm Kessil lamp. The reaction was purified using column chromatography (0-12% diethylether/hexanes gradient) affording a clear, colorless oil (22.1 mg, 41%).

^{1}H NMR (400 MHz, $CDCl_3$) δ 7.26 – 7.18 (m, 3H), 7.18 – 7.13 (m, 2H), 4.25 – 4.10 (m, 2H), 3.93 (td, J = 8.5, 5.4 Hz, 1H), 3.65 (s, 3H), 2.64 – 2.54 (m, 2H), 2.32 (ddd, J = 13.4, 8.4, 7.1 Hz, 1H), 2.19 (d, J = 2.5 Hz, 1H), 2.16 (d, J = 2.7 Hz, 1H).

^{13}C NMR (101 MHz, CDCl$_3$) δ 174.4, 168.5, 135.5, 128.7, 128.1, 126.7, 66.0, 64.4, 52.1, 50.8, 50.1 48.1, 45.4, 25.5.

HRMS (+pAPCI): Calcd for $C_{16}H_{17}O_4$ [M+H] 273.1121, found 273.1120.

methyl-2'-oxo-3-phenyldihydro-2'H,4'H-spiro[bicyclo[1.1.1]pentane-2,3'-pyran]-1-carboxylate (24)

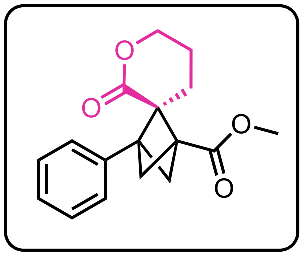

General procedure B was used for the reaction of methyl 3-phenylbicyclo[1.1.0]butane-1-carboxylate (0.20 mmol, 37.6 mg, 1 equiv) with 3-diazotetrahydro-2H-pyran-2-one (0.50 mmol, 63 mg, 2.5 equiv) using TX (5.0 mol%, 2.12 mg) as catalyst and 390 nm Kessil lamp. The reaction was purified using column chromatography (0-20% diethylether/hexanes gradient) affording a clear, colorless oil (35 mg, 61%).

^{1}H NMR (400 MHz, CDCl$_3$) δ 7.40 – 7.32 (m, 2H), 7.27 (dd, J = 7.8, 1.7 Hz, 2H), 3.95 (dt, J = 10.8, 4.0 Hz, 1H), 3.76 (s, 2H), 3.39 – 3.26 (m, 1H), 2.97 (dd, J = 10.4, 3.0 Hz, 1H), 2.55 – 2.36 (m, 2H), 2.24 (d, J = 3.1 Hz, 1H), 2.10 (d, J = 3.1 Hz, 1H), 2.00 – 1.82 (m, 1H), 1.82 – 1.67 (m, 1H).

^{13}C NMR (101 MHz, CDCl$_3$) δ 171.6, 168.9, 136.2, 128.8, 128.2, 126.8, 70.2, 66.6, 51.9, 51.7, 48.4, 22.6, 20.9.

HRMS (+pAPCI): Calcd for $C_{17}H_{19}O_4$ 287.1278 [M+H], found 287.1278.

dimethyl 2-methyl-3-(naphthalen-2-yl)bicyclo[1.1.1]pentane-1,2-dicarboxylate (25)

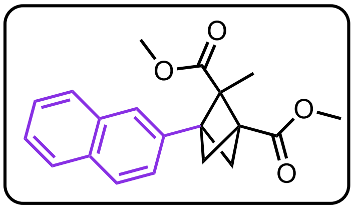

General procedure A was used for the reaction of methyl 3-(naphthalen-2-yl)bicyclo[1.1.0]butane-1-carboxylate (0.20 mmol, 47.7 mg, 1 equiv) with methyl 2-diazopropanoate (0.50 mmol, 57.1 mg, 2.5 equiv) using Ir(ppy)$_3$ (1.0 mol%, 1.31 mg) as catalyst and 440 nm Kessil lamp. The reaction was purified using column chromatography (0-12% diethylether/hexanes gradient) affording a clear, colorless oil (33.8 mg, 52%).

^{1}H NMR (800 MHz, CDCl$_3$) δ 7.86 – 7.79 (m, 3H), 7.70 (s, 1H), 7.49 – 7.44 (m, 2H), 7.42 (d, *J* = 8.2 Hz, 1H), 3.78 (s, 3H), 3.69 (s, 3H), 2.94 (dd, *J* = 10.3, 2.9 Hz, 1H), 2.57 (dd, *J* = 10.3, 3.3 Hz, 1H), 2.33 (d, *J* = 3.3 Hz, 1H), 2.13 (d, *J* = 2.9 Hz, 1H), 1.64 (s, 3H).

^{13}C NMR (201 MHz, CDCl$_3$) δ 174.6, 169.2, 133.9, 133.3, 132.8, 128.0, 127.9, 127.8, 126.3, 126.0, 125.3, 70.2, 51.9, 51.8, 49.3, 49.0, 47.4, 43.1, 13.3.

HRMS (+pAPCI): Calcd for $C_{20}H_{21}O_4$ [M+H] 325.1434, found 325.1435

dimethyl 3-(4-bromophenyl)-2-methylbicyclo[1.1.1]pentane-1,2-dicarboxylate (26)

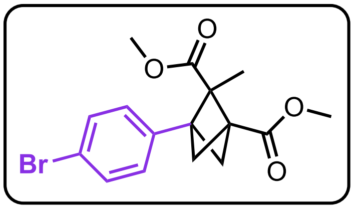

General procedure A was used for the reaction of methyl 3-(4-bromophenyl)bicyclo[1.1.0]butane-1-carboxylate (0.20 mmol, 53.4 mg, 1 equiv) with methyl 2-diazopropanoate (0.50 mmol, 57.1 mg, 2.5 equiv) using Ir(ppy)$_3$ (1.0 mol%, 1.31 mg) as catalyst and 440 nm Kessil lamp. The reaction was purified using column chromatography (0-12% diethylether/hexanes gradient) affording a clear, colorless oil (47.5 mg, 67%).

^{1}H NMR (800 MHz, CDCl$_3$) δ 7.44 (d, *J* = 8.3 Hz, 2H), 7.14 (d, *J* = 8.3 Hz, 2H), 3.75 (s, 3H), 3.68 (s, 3H), 2.80 (dd, *J* = 10.3, 3.0 Hz, 1H), 2.43 (dd, *J* = 10.3, 3.5 Hz, 1H), 2.22 (d, *J* = 3.5 Hz, 1H), 2.04 (d, *J* = 3.0 Hz, 1H), 1.56 (s, 3H).

^{13}C NMR (201 MHz, CDCl$_3$) δ 174.5, 168.9, 135.3, 131.4, 129.1, 121.7, 70.0, 51.9, 51.8, 49.2, 48.3, 47.2, 43.0, 13.2.

HRMS (+pAPCI): Calcd for $C_{16}H_{18}O_4{}^{79}Br$ [M+H] 353.0383, found 353.0383.

dimethyl 2-methyl-3-(p-tolyl)bicyclo[1.1.1]pentane-1,2-dicarboxylate (27)

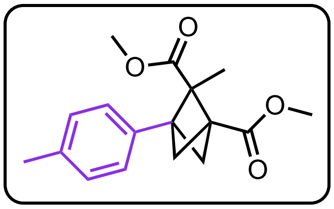

General procedure A was used for the reaction of methyl 3-(p-tolyl)bicyclo[1.1.0]butane-1-carboxylate (0.20 mmol, 40.5 mg, 1 equiv) with methyl 2-diazopropanoate (0.50 mmol, 57.1 mg, 2.5 equiv) using Ir(ppy)$_3$ (1.0 mol%, 1.31 mg) as catalyst and 440 nm Kessil lamp. The reaction was purified using column chromatography (0-12% diethylether/hexanes gradient) affording a clear, colorless oil (44.4 mg, 77%).

^{1}H NMR (400 MHz, CDCl$_3$) δ 7.21 – 7.10 (m, 4H), 3.75 (s, 3H), 3.68 (s, 3H), 2.82 (dd, J = 10.2, 2.9 Hz,1H), 2.45 (dd, J = 10.3, 3.4 Hz, 1H), 2.34 (s, 3H), 2.22 (d, J = 3.5 Hz, 1H), 2.02 (d, J = 2.9 Hz, 1H), 1.57 (s, 3H).

^{13}C NMR (101 MHz, CDCl$_3$) δ 174.6, 169.2, 137.1, 133.2, 128.9, 127.1, 69.8, 51.8, 51.6, 49.1, 48.5, 47.1, 42.9, 21.2, 13.1.

HRMS (+pAPCI): Calcd for $C_{17}H_{21}O_4$ [M+H] 289.1434, found 289.1433

dimethyl 2-methyl-3-(4-(trifluoromethyl)phenyl)bicyclo[1.1.1]pentane-1,2-dicarboxylate (28)

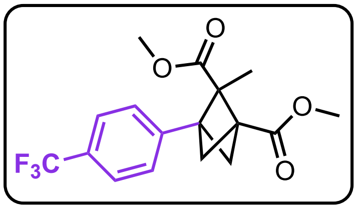

General procedure A was used for the reaction of methyl 3-(4-(trifluoromethyl)phenyl)bicyclo[1.1.0]butane-1-carboxylate (0.20 mmol, 51.2 mg, 1 equiv) with methyl 2-diazopropanoate (0.50 mmol, 57.1 mg, 2.5 equiv) using Ir(ppy)$_3$ (1.0 mol%, 1.31 mg) as catalyst and 440 nm Kessil lamp. The reaction was purified using column chromatography (0-12% diethylether/hexanes gradient) affording a clear, colorless oil (26.6 mg, 39%).

^{1}H NMR (800 MHz, CDCl$_3$) δ 7.58 (d, *J* = 8.0 Hz, 2H), 7.39 (d, *J* = 8.0 Hz, 2H), 3.76 (s, 3H), 3.69 (s, 3H), 2.86 (dd, *J* = 10.3, 3.0 Hz, 1H), 2.48 (dd, *J* = 10.3, 3.4 Hz, 1H), 2.27 (d, *J* = 3.4 Hz, 1H), 2.08 (d, *J* = 2.9 Hz, 1H), 1.59 (s, 3H).

^{13}C NMR (201 MHz, CDCl$_3$) δ 174.4, 168.8, 140.3, 129.7 (q, *J* = 32.4 Hz), 127.8, 125.3 (q, *J* = 3.7 Hz), 124.3 (q, *J* = 271.9 Hz), 70.2, 52.0, 51.9, 49.3, 48.4, 47.3, 43.1, 13.2.

^{19}F NMR (753 MHz, CDCl$_3$) δ -62.54.

HRMS (+pAPCI): Calcd for $C_{17}H_{18}O_4F_3$ [M+H] 343.1152, found 343.1150.

dimethyl 3-(3,4-dichlorophenyl)-2-methylbicyclo[1.1.1]pentane-1,2-dicarboxylate (29)

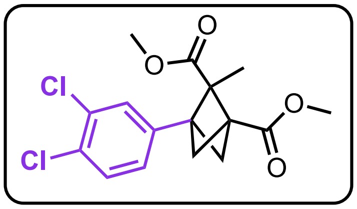

General procedure A was used for the reaction of methyl 3-(3,4-dichlorophenyl)bicyclo[1.1.0]butane-1-carboxylate (0.20 mmol, 51.4 mg, 1 equiv) with methyl 2-diazopropanoate (0.50 mmol, 57.1 mg, 2.5 equiv) using Ir(ppy)$_3$ (1.0 mol%, 1.31 mg) as catalyst and 440 nm Kessil lamp. The reaction was purified using column chromatography (0-12% diethylether/hexanes gradient) affording a clear, colorless oil (39.8 mg, 58%).

^{1}H NMR (400 MHz, CDCl$_3$) δ 7.38 (d, J = 8.2 Hz, 1H), 7.34 (d, J = 2.0 Hz, 1H), 7.11 (dd, J = 8.2, 2.0 Hz, 1H), 3.75 (s, 3H), 3.69 (s, 3H), 2.80 (dd, J = 10.2, 3.0 Hz, 1H), 2.43 (dd, J = 10.3, 3.5 Hz, 1H), 2.23 (d, J = 3.5 Hz, 1H), 2.05 (d, J = 3.0 Hz, 1H), 1.56 (s, 3H).

^{13}C NMR (101 MHz, CDCl$_3$) δ 174.2, 168.5, 136.5, 132.3, 131.6, 130.2, 129.3, 126.8, 70.0, 51.83, 51.80, 49.3, 47.7, 47.1, 42.9, 13.0.

HRMS (+pAPCI): Calcd for $C_{16}H_{17}O_4{}^{35}Cl_2$ [M+H] 343.0498, found 343.0498.

1-methyl 2-(2,2,2-trichloroethyl) 3-(o-tolyl)bicyclo[1.1.1]pentane-1,2-dicarboxylate (30)

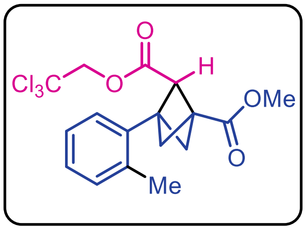

General procedure B was used for the reaction of methyl 3-(o-tolyl)bicyclo[1.1.0]butane-1-carboxylate (0.20 mmol, 40.5 mg, 1 equiv) with 2,2,2-trichloroethyl 2-diazoacetate (0.50 mmol, 109 mg, 2.5 equiv) using TX (5.0 mol%, 2.12 mg) as catalyst and 390 nm Kessil lamp. The reaction was purified using column chromatography (0-20% diethyl ether/hexanes gradient) affording a clear, colorless oil (53.7 mg, 71%).

^{1}H NMR (400 MHz, $CDCl_3$) δ 7.20 – 7.08 (m, 4H), 4.72 (d, *J* = 12.0 Hz, 1H), 4.64 (d, *J* = 11.9 Hz, 1H), 3.75 (s, 3H), 3.12 (dd, *J* = 9.9, 3.1 Hz, 1H), 2.57 (dd, *J* = 7.0, 3.1 Hz, 1H), 2.53 (dd, *J* = 9.8, 2.1 Hz, 1H), 2.43 (s, 3H), 2.31 (d, *J* = 2.1 Hz, 1H).

^{13}C NMR (101 MHz, CDCl3) δ 168.7, 167.8, 137.1, 134.0, 130.9, 128.5, 128.1, 126.0, 94.7, 74.0, 63.1, 53.6, 52.2, 48.9, 47.9, 41.3, 20.7.

HRMS (+pAPCI): Calcd for $C_{17}H_{18}O_4{}^{35}Cl_3$ [M+H] 391.0265, found 391.0261.

1-benzyl 2-ethyl (E)-1-(4-ethoxy-4-oxobut-2-en-1-yl)cyclopropane-1,2-dicarboxylate (33)

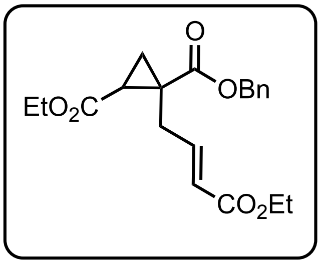

General procedure A was used for the reaction of methyl benzyl bicyclo[1.1.0]butane-1-carboxylate (0.20 mmol, 37.6 mg, 1 equiv) with ethyl 2-diazoacetate (0.50 mmol, 65 mg, 2.5 equiv) using Ir(ppy)$_3$ (1.0 mol%, 1.31 mg) as catalyst and 440 nm Kessil lamp. The reaction was purified using column chromatography (0-12% diethylether/hexanes gradient) affording a clear, colorless oil (8.8 mg, 12%).

^{1}H NMR (400 MHz, $CDCl_3$) δ 7.43 – 7.28 (m, 5H), 6.99 – 6.82 (m, 1H), 5.79 (dt, J = 15.7, 1.6 Hz, 1H), 5.19 – 5.04 (m, 2H), 4.20 – 4.11 (m, 4H), 2.89 (ddd, J = 16.2, 6.1, 1.7 Hz, 1H), 2.61 (ddd, J = 16.3, 6.9, 1.6 Hz, 1H), 2.48 (dd, J = 8.7, 6.7 Hz, 1H), 1.63 (dd, J = 8.7, 4.4 Hz, 1H), 1.32 – 1.18 (m, H).

^{13}C NMR (101 MHz, $CDCl_3$) δ 172.3, 170.7, 166.8, 146.0, 135.8, 129.1, 128.9, 128.7, 123.3, 67.9, 61.7, 60.7, 30.7, 30.3, 27.5, 21.2, 14.7, 14.6.

HRMS (-pAPCI): Calcd for $C_{20}H_{23}O_6$ [M–H] 359.1500, found 359.1497.

6. One-pot procedure

One Pot Procedure

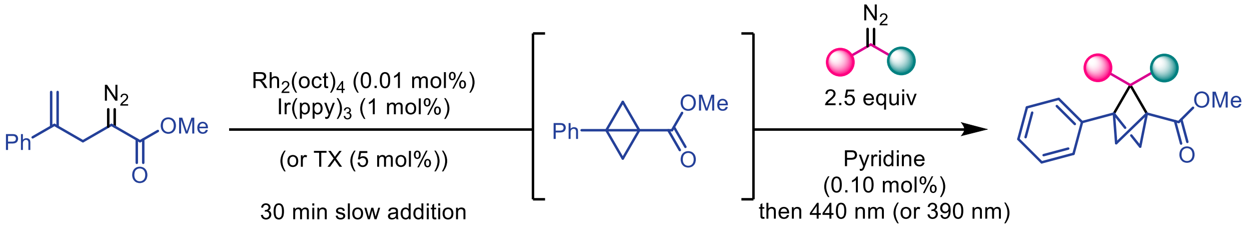

To an oven, a dried photoreaction tube was added Ir(ppy)$_3$ (2.0 μmol, 1.3 mg, 1 mol%) (or TX(10.0 μmol, 2.1 mg, 5 mol%)) photocatalyst. This was purged and backfilled three times with nitrogen. Then, 2 mL of dry degassed DCM was added to the vial along with Rh$_2$(Oct)$_4$ (0.02 μmol, 17.9 μL, 1 mg/ml stock solution in DCM, 0.0001 equiv). Then, methyl 2-diazo-4-phenylpent-4-enoate (0.20 mmol, 43.2 mg, 1.0 equiv) was dissolved in 1 mL of DCM and added to the flask over a period of 30 minutes. The reaction was left to stir for an additional 15 minutes after the addition had finished. Then, pyridine (0.20 μmol, 16 μL, 1 mg/ml stock solution in DCM, 0.001 equiv) was added and the corresponding diazo (0.50 mmol 2.5 equiv). The reaction was placed in a -65 °C acetone (or ethanol) bath and irradiated with either 440 nm (or 390 nm) light for 20 h. After this time the reaction was warmed to room temperature and taken for crude NMR analysis. The crude solution was dry loaded onto silica and columned to afford the desired BCP product

2-ethyl 1-methyl 3-phenylbicyclo[1.1.1]pentane-1,2-dicarboxylate (15)

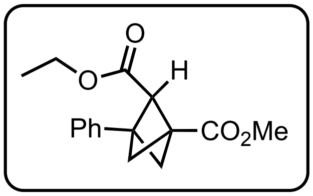

One pot procedure was used for the reaction of methyl 2-diazo-4-phenylpent-4-enoate (0.20 mmol, 43.2 mg, 1.0 equiv) with ethyl 2-diazoacetate (0.50 mmol, 55.6 μL 83% wt in toluene, 2.5 equiv) using Ir(ppy)$_3$ (1.0 mol%, 1.31 mg) as catalyst and 440 nm Kessil lamp. The reaction was purified using column chromatography (0-12% diethyl ether/hexanes gradient) affording a clear, colorless oil (17.3 mg, 32%).

dimethyl 2-methyl-3-phenylbicyclo[1.1.1]pentane-1,2-dicarboxylate (16)

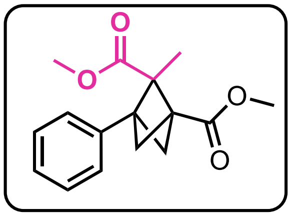

One pot procedure was used for the reaction of methyl 2-diazo-4-phenylpent-4-enoate (0.20 mmol, 43.2 mg, 1.0 equiv) with methyl 2-diazopropanoate (0.50 mmol, 57.1 mg, 2.5 equiv) using Ir(ppy)3 (1.0 mol%, 1.31 mg) as catalyst and 440 nm Kessil lamp. The reaction was purified using column chromatography (0-12% diethyl ether/hexanes gradient) affording a clear, colorless oil (37.7 mg, 69%).

2-benzyl 1-methyl 3-phenylbicyclo[1.1.1]pentane-1,2-dicarboxylate (18)

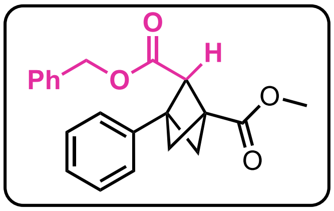

One pot procedure was used for the reaction of methyl 2-diazo-4-phenylpent-4-enoate (0.20 mmol, 43.2 mg, 1.0 equiv) with benzyl 2-diazoacetate (0.50 mmol, 88.1 mg, 2.5 equiv) using TX (5.0 mol%, 2.12 mg) as catalyst and 390 nm Kessil lamp. The reaction was purified using column chromatography (0-12% diethyl ether/hexanes gradient) affording a clear, colorless oil (41.3 mg, 61%).

1-methyl 2-(2,2,2-trichloroethyl) 3-phenylbicyclo[1.1.1]pentane-1,2-dicarboxylate (20)

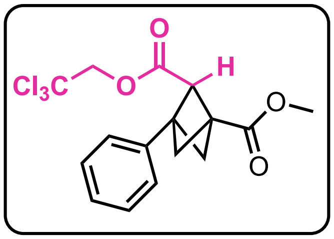

One pot procedure was used for the reaction of methyl 2-diazo-4-phenylpent-4-enoate (0.20 mmol, 43.2 mg, 1.0 equiv) with 2,2,2-trichloroethyl 2-diazoacetate (0.50 mmol, 109 mg, 2.5 equiv) using TX (5.0 mol%, 2.12 mg) as catalyst and 390 nm Kessil lamp. The reaction was purified using column chromatography (0-12% diethyl ether/hexanes gradient) affording a clear, colorless oil (45.8 mg, 61%).

2.00 mmol scale reaction. One pot procedure was used with slightly modification in concentrations from 0.05 M to 0.10 M. Particularly, to a flamed dried 25 ml Schlenk tube was added TX (100.0 μmol, 21.2 mg, 5 mol%) photocatalyst. This was purged and backfilled three times with nitrogen. Then, 10 mL of dry degassed DCM was added to the

vial along with $Rh_2(Oct)_4$ (0.2 µmol, 179 µL, 1 mg/ml stock solution in DCM, 0.0001 equiv). Then, methyl 2-diazo-4-phenylpent-4-enoate (0.20 mmol, 43.2 mg, 1.0 equiv) was dissolved in 5 mL of DCM and added to the flask over a period of 30 minutes. The reaction was left to stir for an additional 15 minutes after the addition had finished. Then, pyridine (2.0 µmol, 160 µL, 1 mg/ml stock solution in DCM, 0.001 equiv) was added and 2,2,2-trichloroethyl 2-diazoacetate (5.0 mmol, 1.09 g, 2.5 equiv). The mixture was parging with N_2 for 5 min before it was in a -65 °C ethanol bath and irradiated with 390 nm light for 18 h. The crude mixture was directly dried loaded and purified using column chromatography (0-12% diethyl ether/hexanes gradient) affording a clear, colorless oil (410.3 mg, 54%).

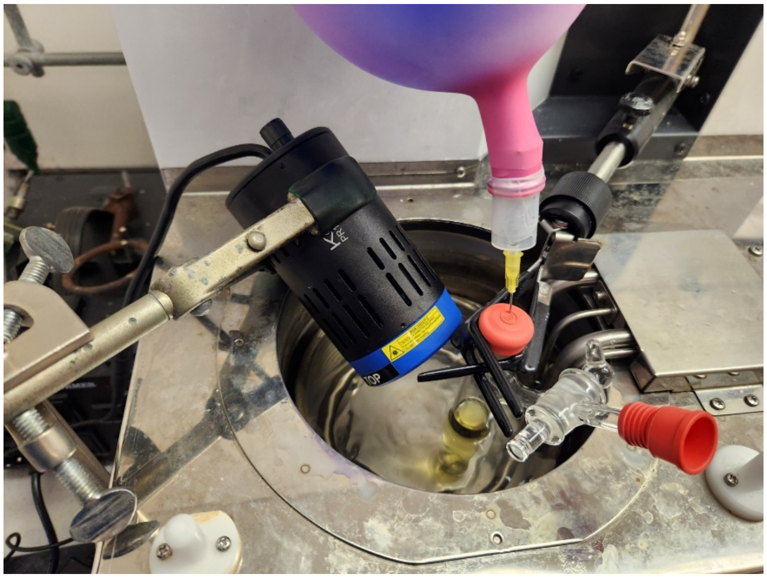

methyl 3-phenyl-2-(trifluoromethyl)bicyclo[1.1.1]pentane-1-carboxylate (22)

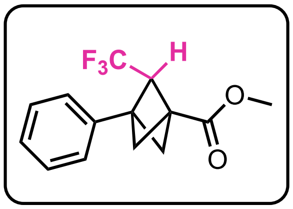

One pot procedure was used for the reaction of methyl 2-diazo-4-phenylpent-4-enoate (0.20 mmol, 43.2 mg, 1.0 equiv) with 2-diazo-1,1,1-trifluoroethane (1.0 mmol, 1.37 mL of 0.73 M solution, 5 equiv) using TX (5.0 mol%, 2.12 mg) as catalyst and 390 nm Kessil lamp. The reaction was purified using column chromatography (0-12% diethylether/hexanes gradient) affording a clear, colorless oil (22.5 mg, 42%).

methyl-2'-oxo-3-phenyldihydro-2'H-spiro[bicyclo[1.1.1]pentane-2,3'-furan]-1-carboxylate (23)

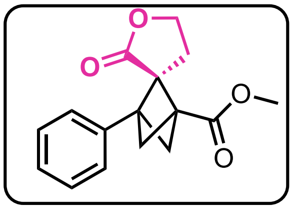

One pot procedure was used for the reaction of methyl 2-diazo-4-phenylpent-4-enoate (0.20 mmol, 43.2 mg, 1.0 equiv) with 3-diazodihydrofuran-2(3H)-one (0.50 mmol, 56.0 mg, 2.5 equiv) using TX (5.0 mol%, 2.12 mg) as catalyst 390 nm Kessil lamp. The reaction was purified using column chromatography (0-12% diethylether/hexanes gradient) affording a clear, colorless oil (15.1 mg, 28%).

methyl-2'-oxo-3-phenyldihydro-2'H,4'H-spiro[bicyclo[1.1.1]pentane-2,3'-pyran]-1-carboxylate (24)

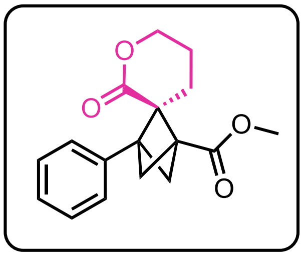

One pot procedure was used for the reaction of methyl 2-diazo-4-phenylpent-4-enoate (0.20 mmol, 43.2 mg, 1.0 equiv) with 3-diazotetrahydro-2H-pyran-2-one (0.50 mmol, 63 mg, 2.5 equiv) using TX (5.0 mol%, 2.12 mg) as catalyst and 390 nm Kessil lamp. The reaction was purified using column chromatography (0-20% diethylether/hexanes gradient) affording a clear oil which solidified under vacuum (39.3 mg, 69%).

methyl 2-cyano-3-phenylbicyclo[1.1.1]pentane-1-carboxylate (34)

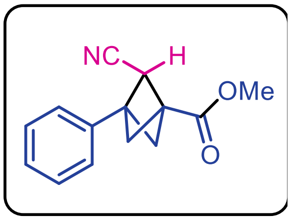

One pot procedure was used for the reaction of methyl 2-diazo-4-phenylpent-4-enoate (0.20 mmol, 43.2 mg, 1.0 equiv) with 2-diazoacetonitrile (1.0 mmol, 2.0 ml, 0.50 M solution in DCM, 5.0 equiv) using TX (5.0 mol%, 2.12 mg) as catalyst and 390 nm Kessil lamp. The reaction was purified using column chromatography (0-20% diethylether/hexanes gradient) affording a clear oil (26.2 mg, 58%).

^{1}H NMR (400 MHz, CDCl$_3$) δ 7.43 – 7.31 (m, 3H), 7.31 – 7.26 (m, 2H), 3.78 (s, 3H), 3.21 (d, *J* = 7.6 Hz, 1H), 3.08 (dd, *J* = 9.8, 3.4 Hz, 1H), 2.49 (dd, *J* = 7.6, 3.3 Hz, 1H), 2.40 (d, *J* = 2.6 Hz, 1H), 2.36 (dd, *J* = 9.8, 2.6 Hz, 1H).

^{13}C NMR (101 MHz, CDCl$_3$) δ 167.6, 135.3, 128.8, 128.5, 126.2, 117.8, 52.5, 52.0, 51.4, 50.1, 46.5, 40.7.

HRMS (+pAPCI): Calcd for $C_{14}H_{14}O_2N$ [M+H] 228.1019, found 228.1020.

2-ethyl 1-methyl 3-phenyl-2-(trimethylsilyl)bicyclo[1.1.1]pentane-1,2-dicarboxylate (35)

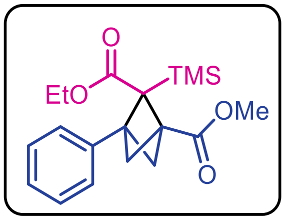

One pot procedure was used for the reaction of methyl 2-diazo-4-phenylpent-4-enoate (0.20 mmol, 43.2 mg, 1.0 equiv) with 2,2,2-trichloroethyl 2-diazo-2-(trimethylsilyl)acetate (0.5 mmol, 149 mg, 4.0 equiv) using TX (5.0 mol%, 2.12 mg) as catalyst and 390 nm Kessil lamp. The reaction was purified using column chromatography (0-15% diethylether/hexanes gradient) then the product is further purified by preparative TLC using (2 hexane/ 3 DCM) as an eluent to affording a clear oil (9.1 mg, 13%). Note. The product is weakly active with 254 nm, and strongly active with CAM stain.

^{1}H NMR (800 MHz, CDCl$_3$) 7.39 (d, *J* = 7.5 Hz, 2H), 7.31 (t, *J* = 7.5 Hz, 2H), 7.26 – 7.22 (m, 1H), 4.16 (dt, *J* = 7.1, 7.1 Hz, 2H), 3.75 (s, 3H), 2.79 (dd, *J* = 10.3, 2.6 Hz, 1H), 2.71 (dd, *J* = 10.3, 3.0 Hz, 1H), 2.25 (d, *J* = 3.0 Hz, 1H), 2.14 (d, *J* = 2.5 Hz, 1H), 1.26 (t, *J* = 7.1 Hz, 3H), 0.09 (s, 9H).

^{13}C NMR (201 MHz, CDCl$_3$) δ 173.9, 169.7, 138.0, 129.8, 128.0, 127.7, 127.4, 71.6, 60.1, 55.0, 51.8, 49.9, 48.4, 43.9, 14.3, 0.2, -0.0.

HRMS (+pAPCI): Calcd for $C_{19}H_{27}O_4{}^{28}Si$ [M+H] 347.1673, found 347.1677.

Unsuccessful examples

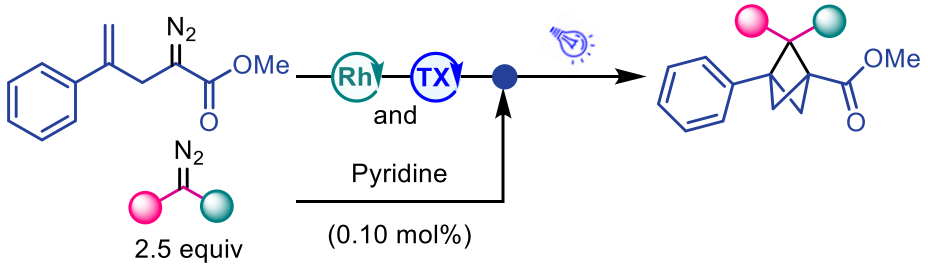

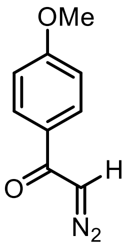

0% yield

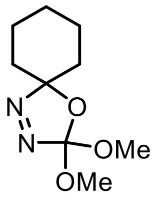

0% yield

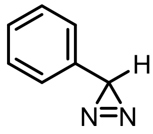

0% yield

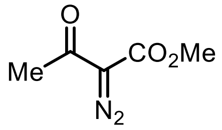

0% yield

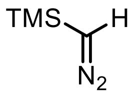

<10% yield

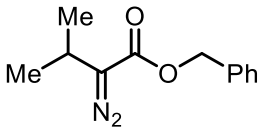

low yielding

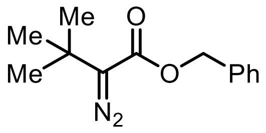

low yielding

Major by product observed in the standard reaction

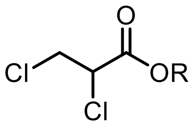

DCM insertion

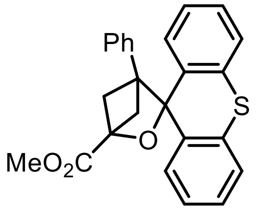

addition of BCB to TX

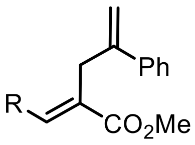

diene from fragmentation

7. Copies of NMR of Novel Compounds

Compound **15**

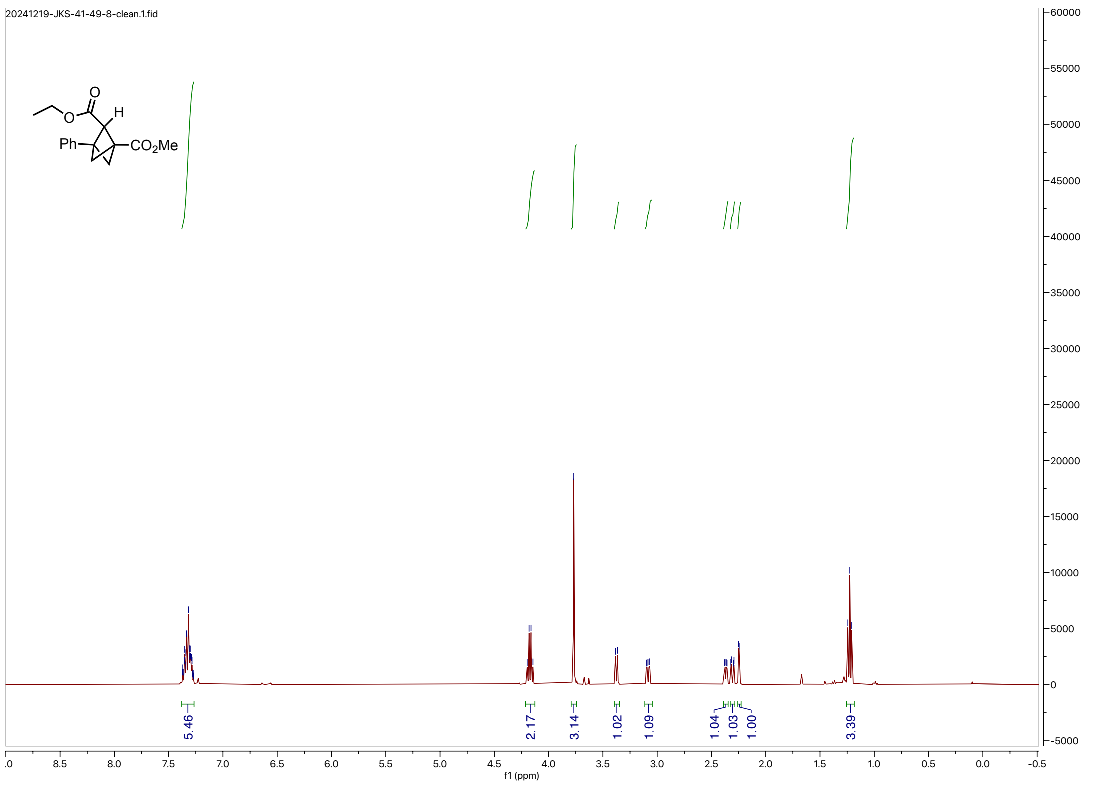

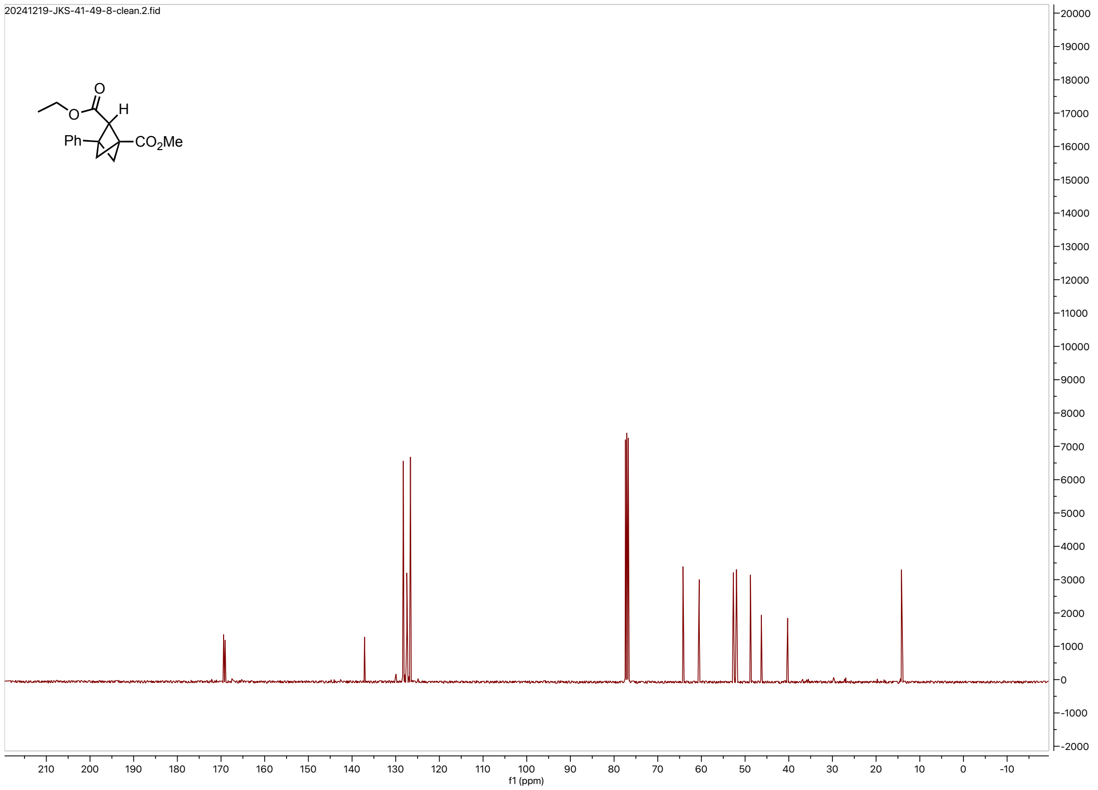

Compound **16**

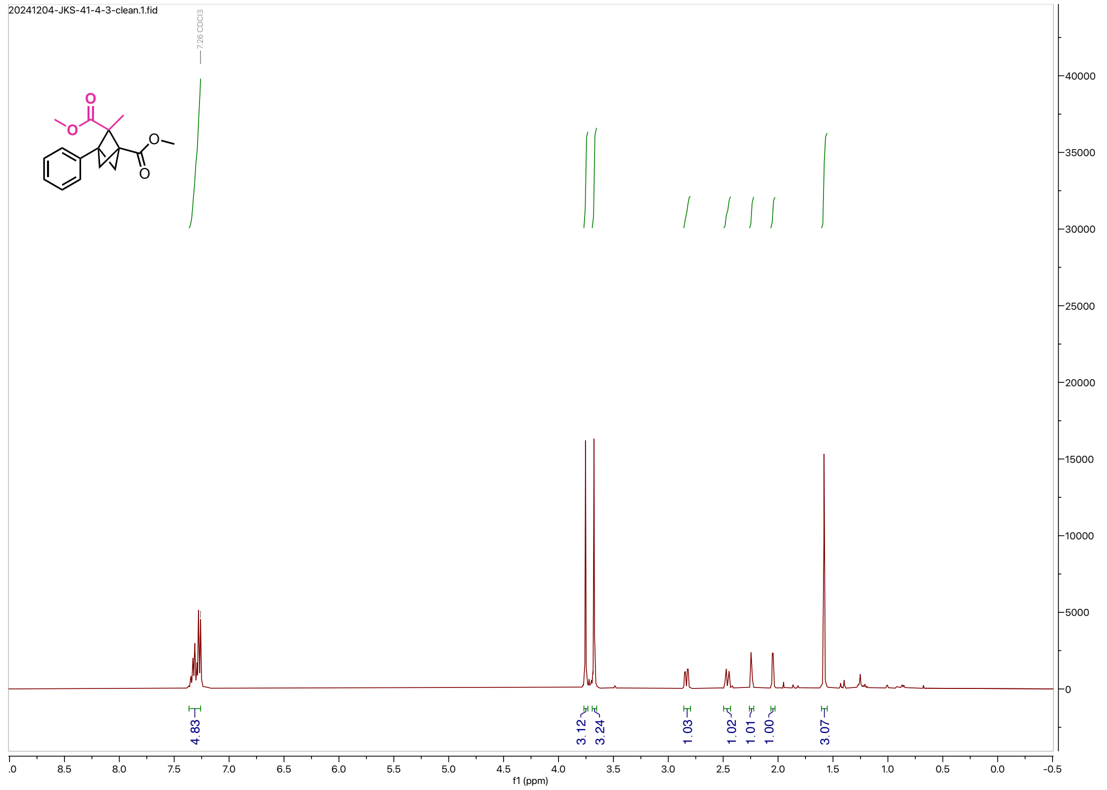

20241204-JKS-41-4-3-clean.1.fid
7.26 CDCl3
4.83
3.12
3.24
1.03
1.02
1.01
1.00
3.07
f1 (ppm)

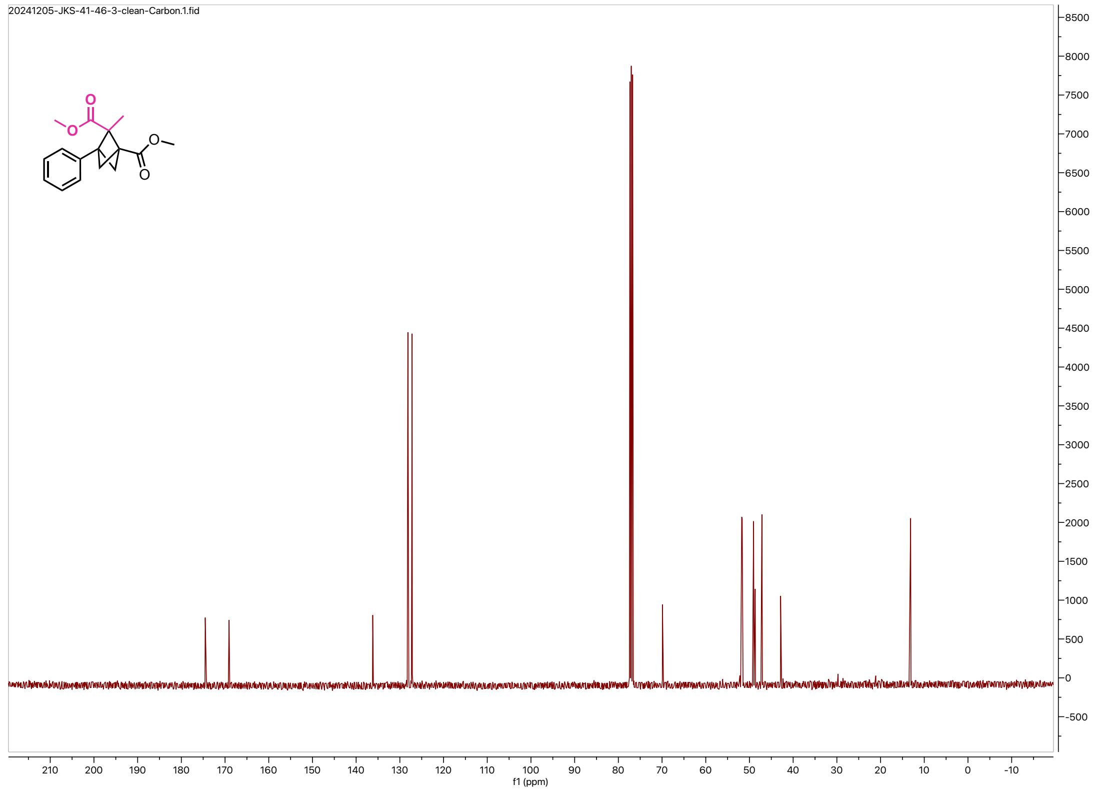

20241205-JKS-41-46-3-clean-Carbon.1.fid
f1 (ppm)

Compound **17**

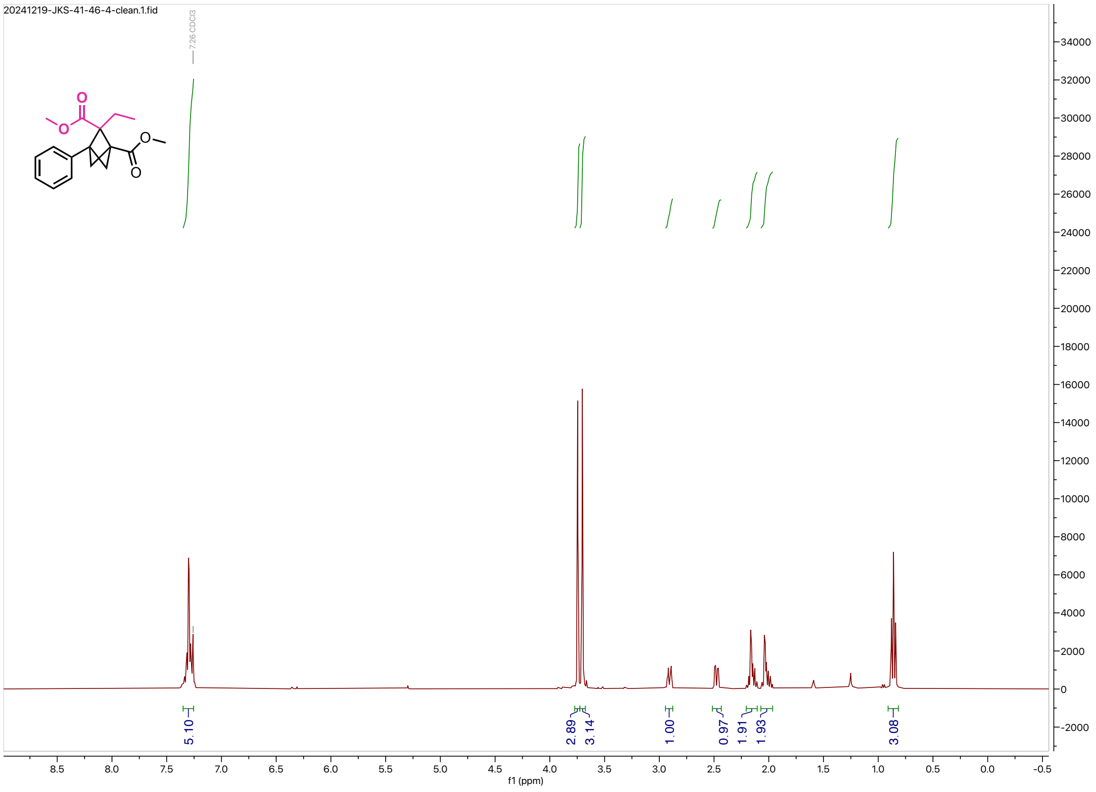
20241219-JKS-41-46-4-clean.1.fid
7.26 CDCl3
5.10
2.89
3.14
1.00
0.97
1.91
1.93
3.08
f1 (ppm)

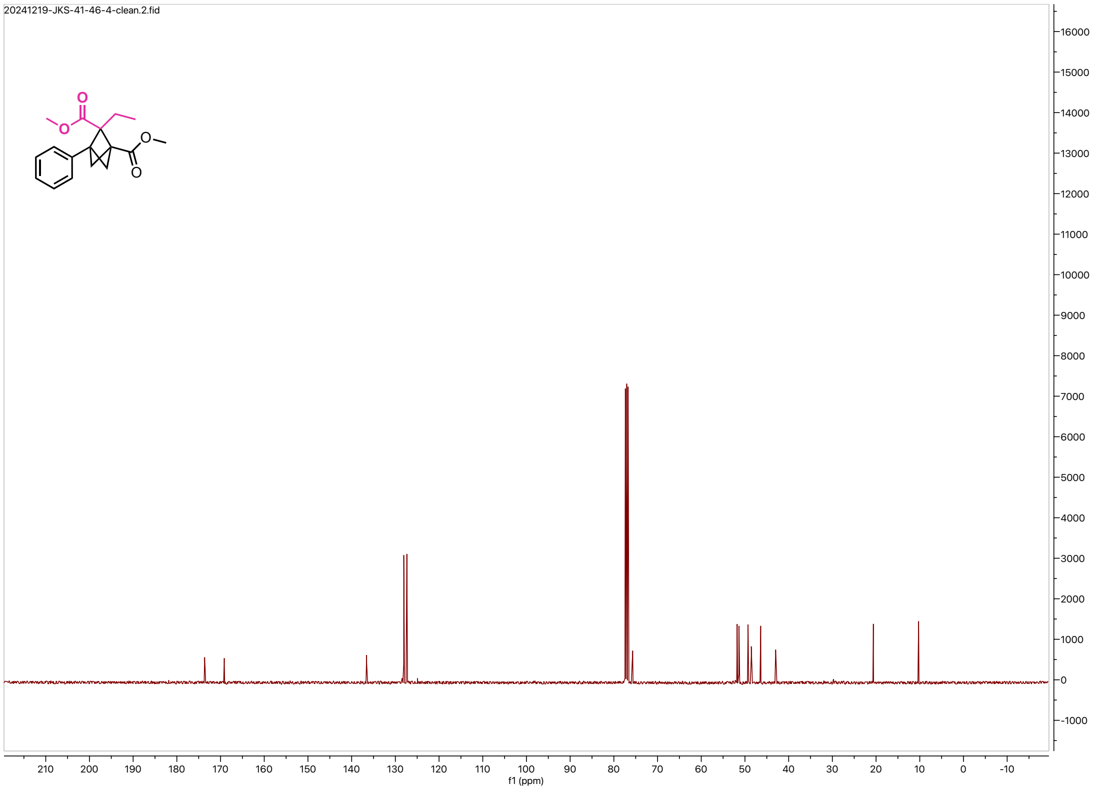
20241219-JKS-41-46-4-clean.2.fid
f1 (ppm)

Compound **18**

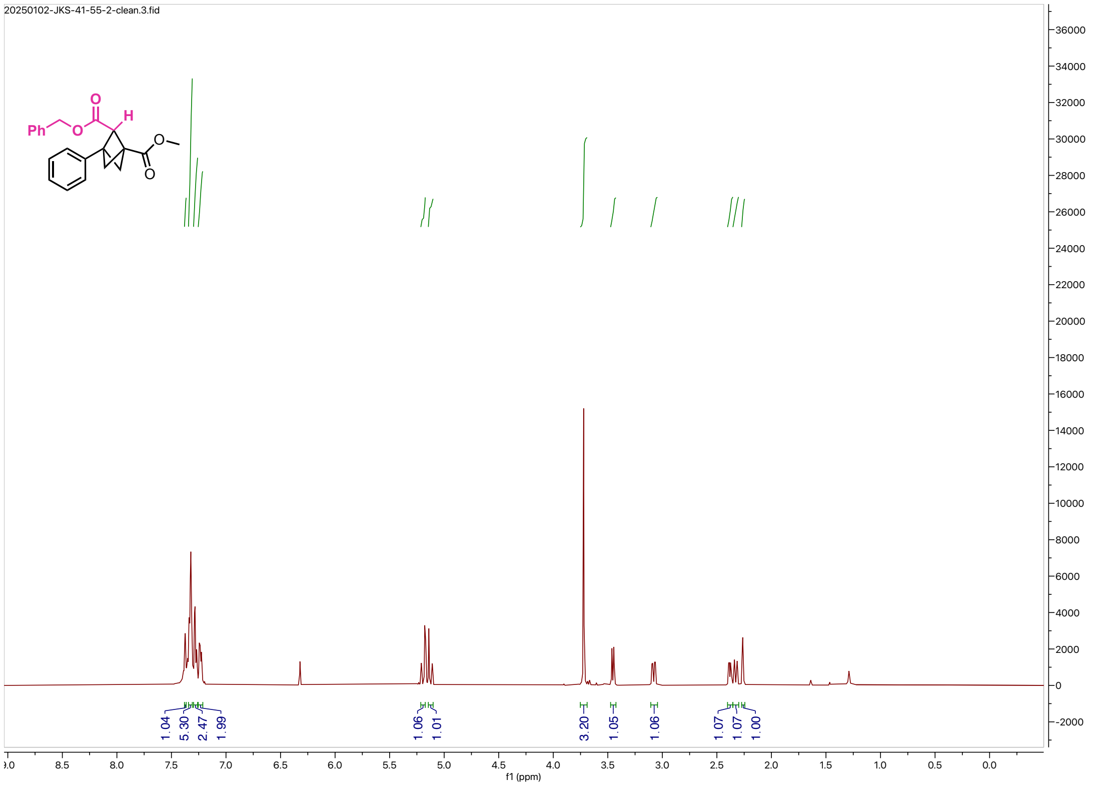

20250102-JKS-41-55-2-clean.3.fid
Ph
O
O
H
O
O
1.04
5.30
2.47
1.99
1.06
1.01
3.20
1.05
1.06
1.07
1.07
1.00
f1 (ppm)

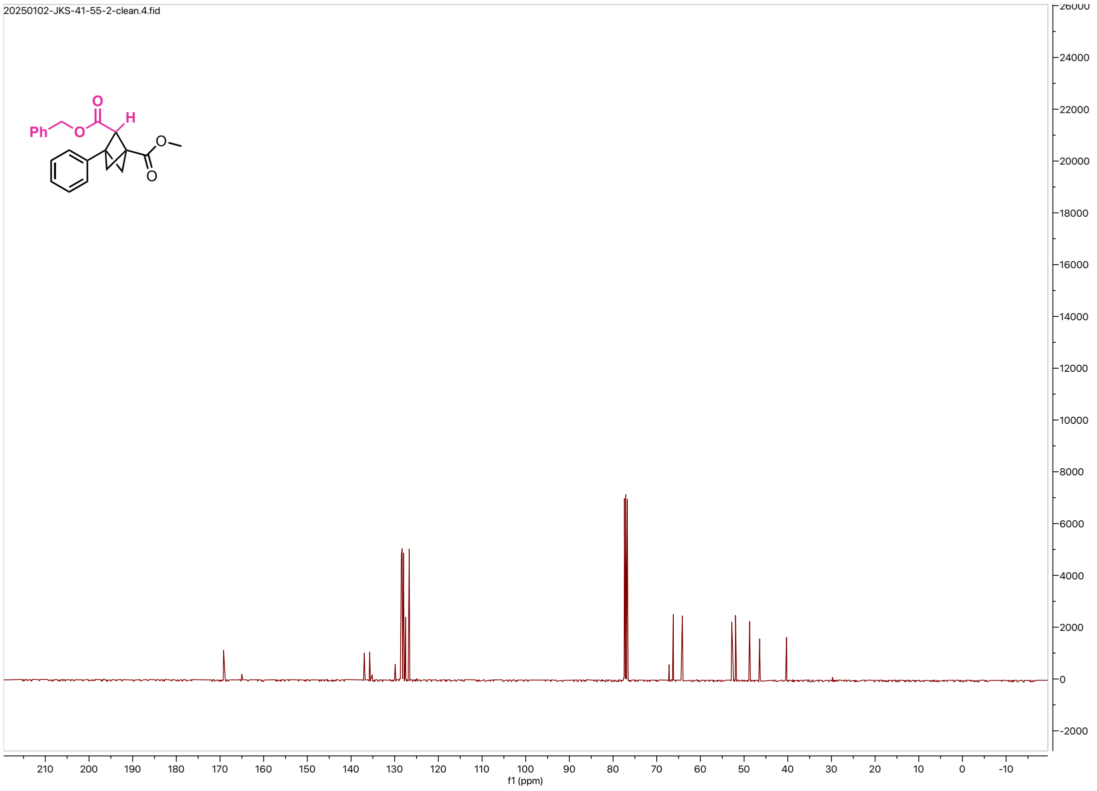

20250102-JKS-41-55-2-clean.4.fid
Ph
O
O
H
O
O
f1 (ppm)

Compound **19**

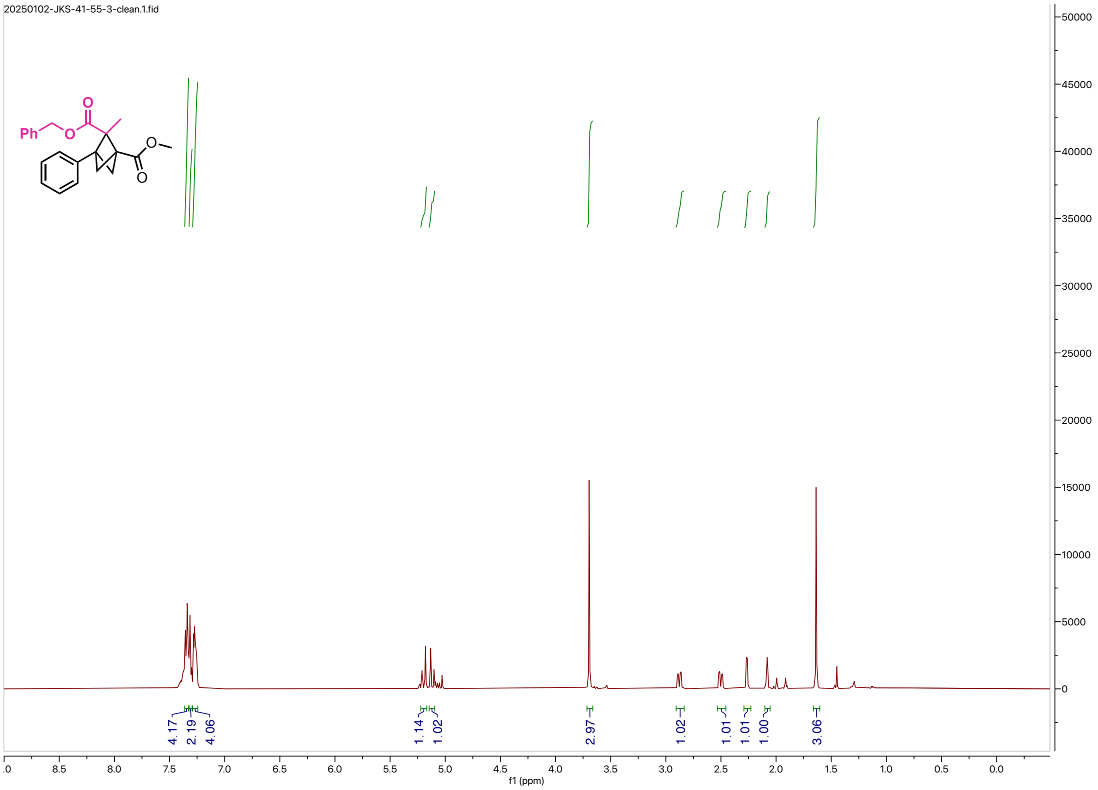

20250102-JKS-41-55-3-clean.1.fid
Ph
O
O
O
O
4.17
2.19
4.06
1.14
1.02
2.97
1.02
1.01
1.01
1.00
3.06
f1 (ppm)

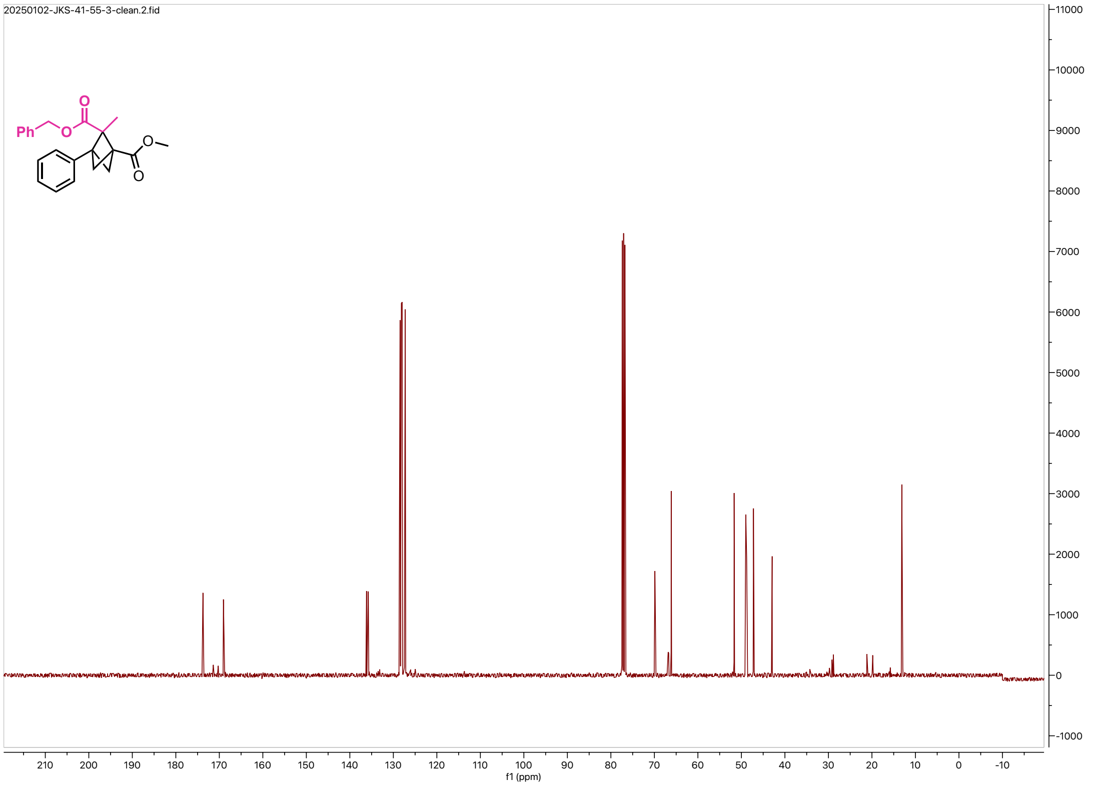

20250102-JKS-41-55-3-clean.2.fid
Ph
O
O
O
O
f1 (ppm)

Compound **20**

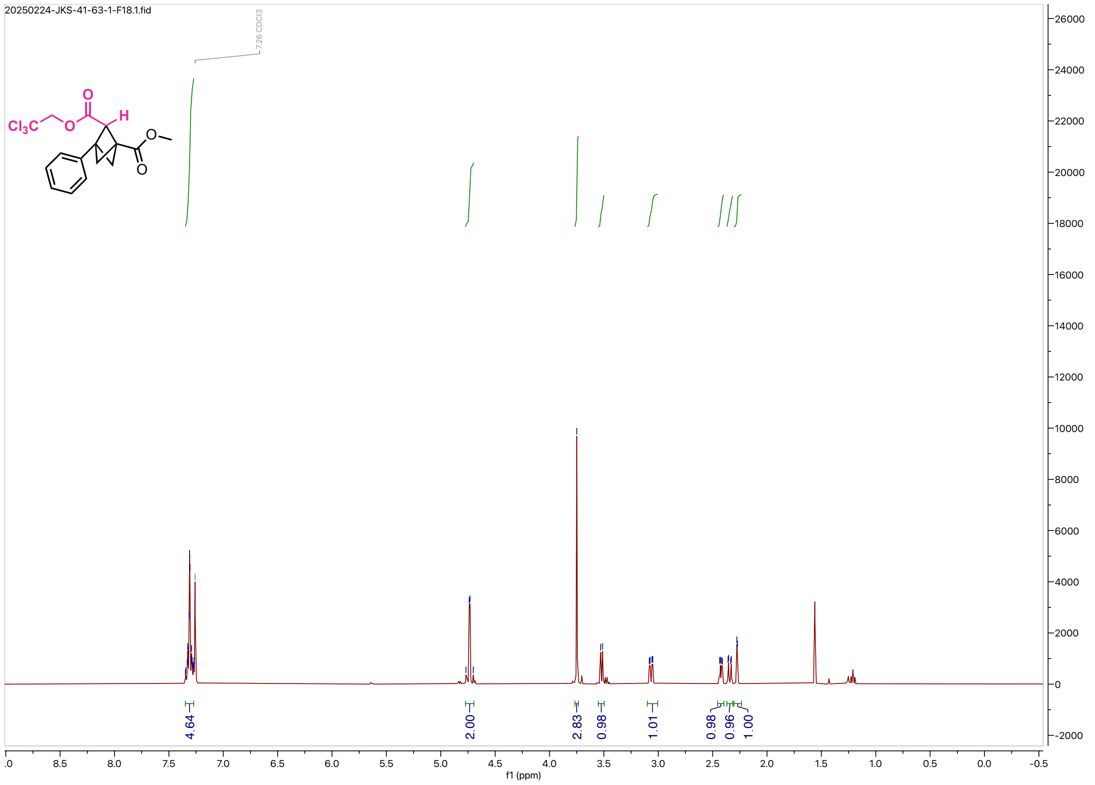
20250224-JKS-41-63-1-F18.1.fid
7.26 CDCl3
4.64
2.00
2.83
0.98
1.01
0.98
0.96
1.00
f1 (ppm)

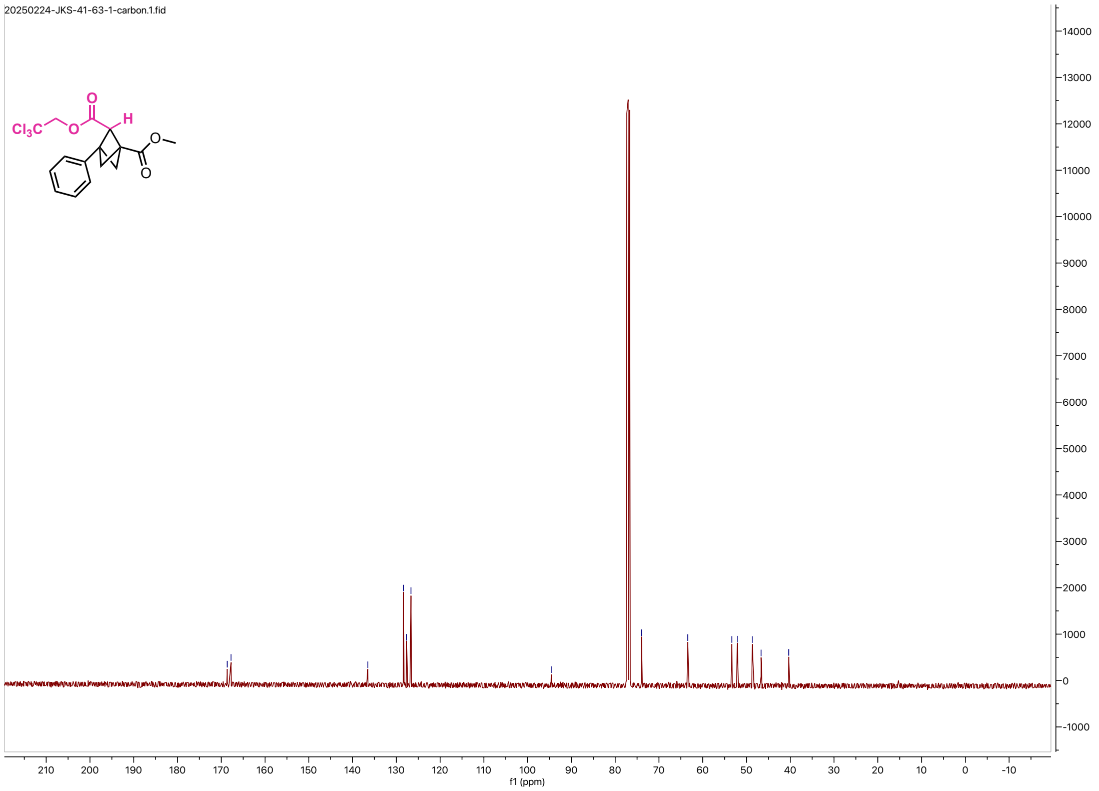
20250224-JKS-41-63-1-carbon.1.fid
f1 (ppm)

Compound **21**

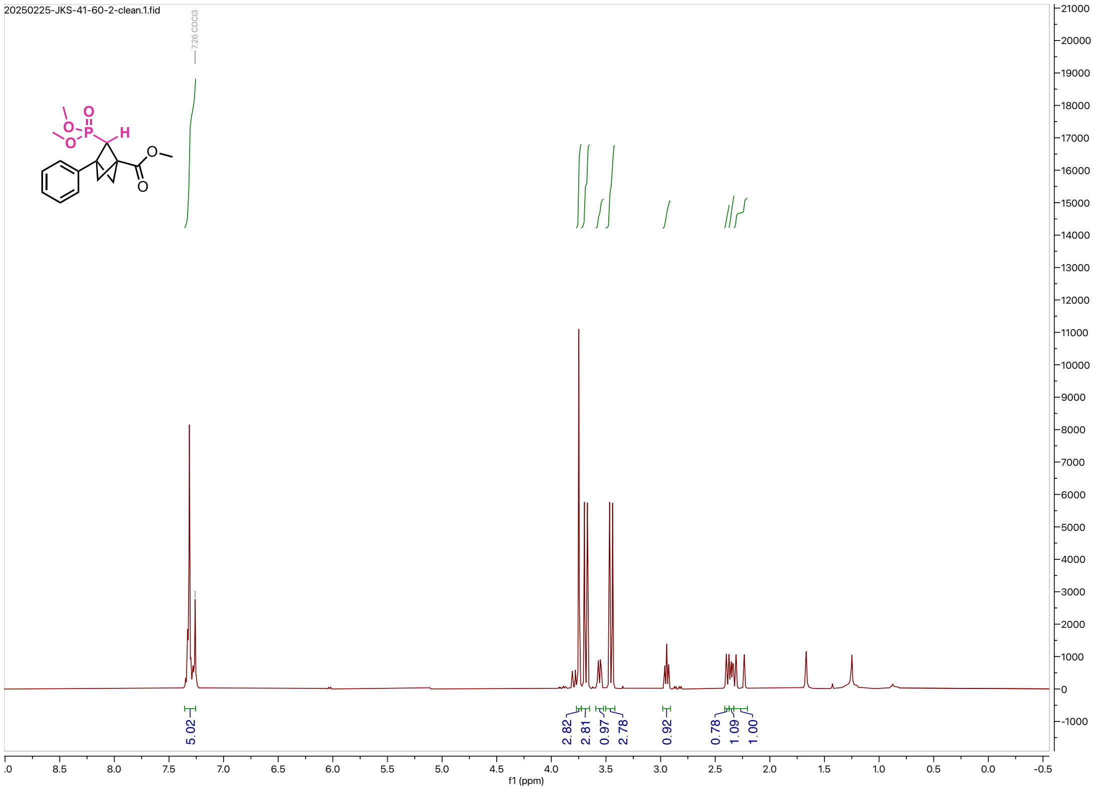
20250225-JKS-41-60-2-clean.1.fid
7.26 CDCl3
5.02
2.82
2.81
0.97
2.78
0.92
0.78
1.09
1.00
f1 (ppm)

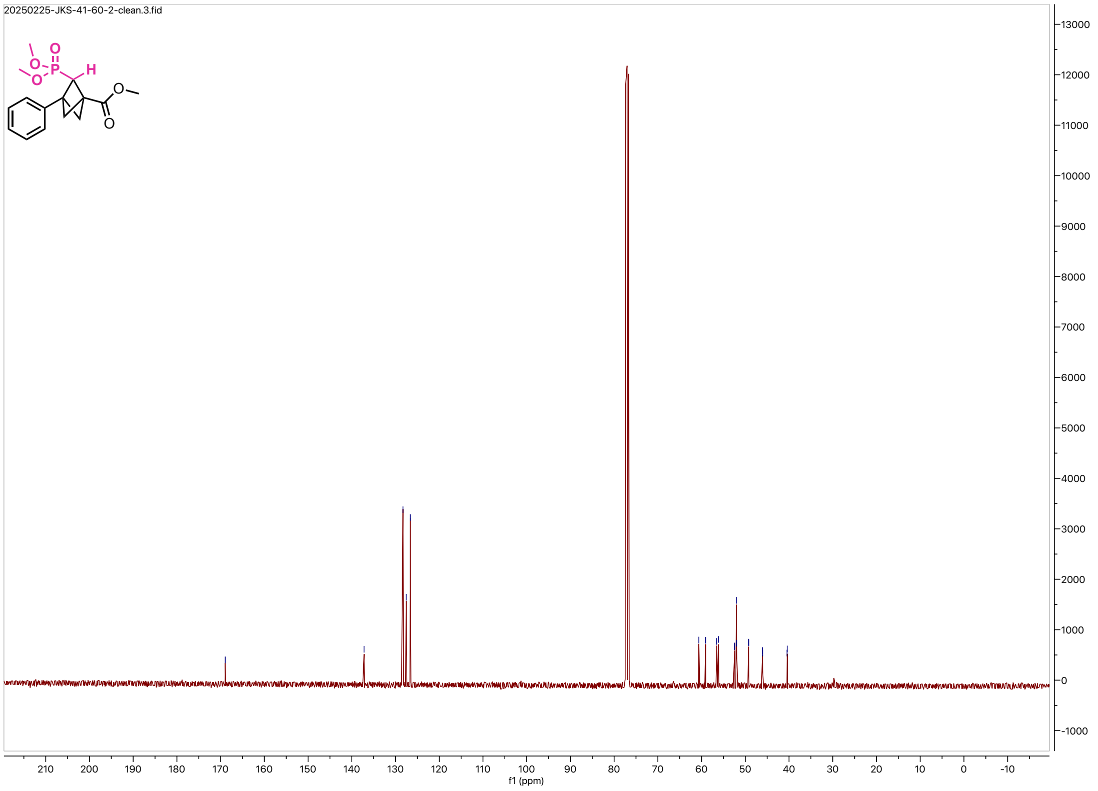
20250225-JKS-41-60-2-clean.3.fid
f1 (ppm)

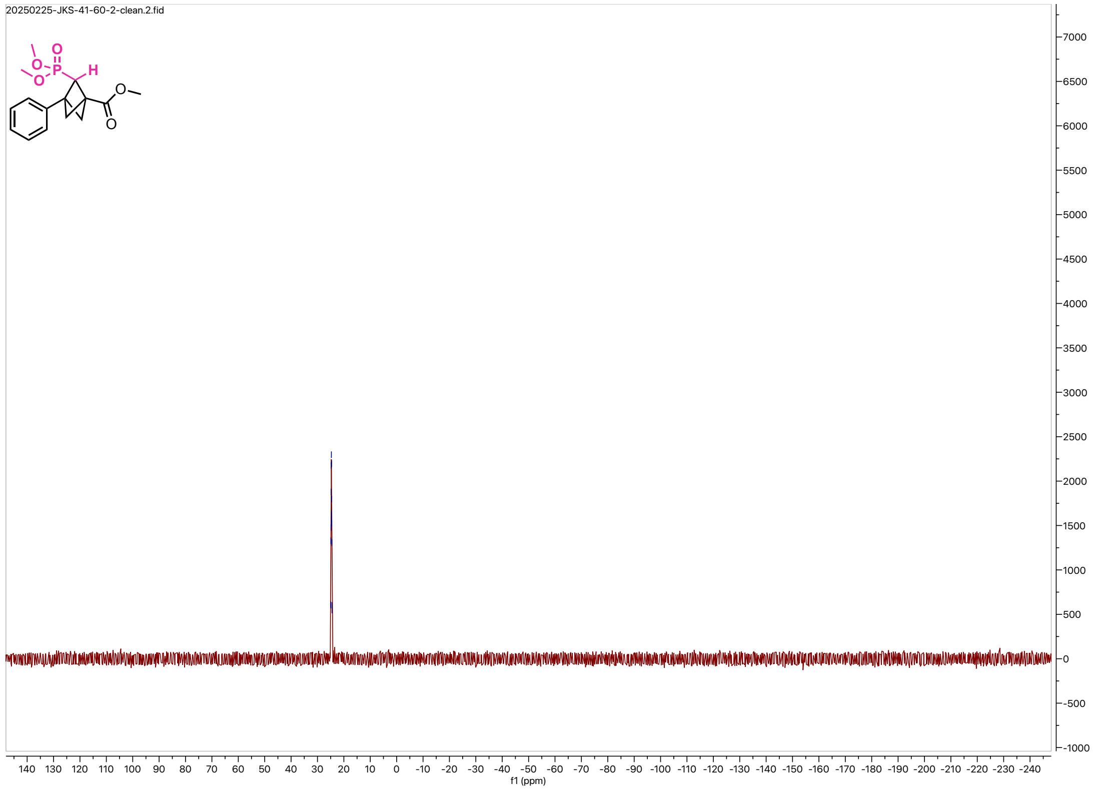

20250225-JKS-41-60-2-clean.2.fid
f1 (ppm)

Compound **22**

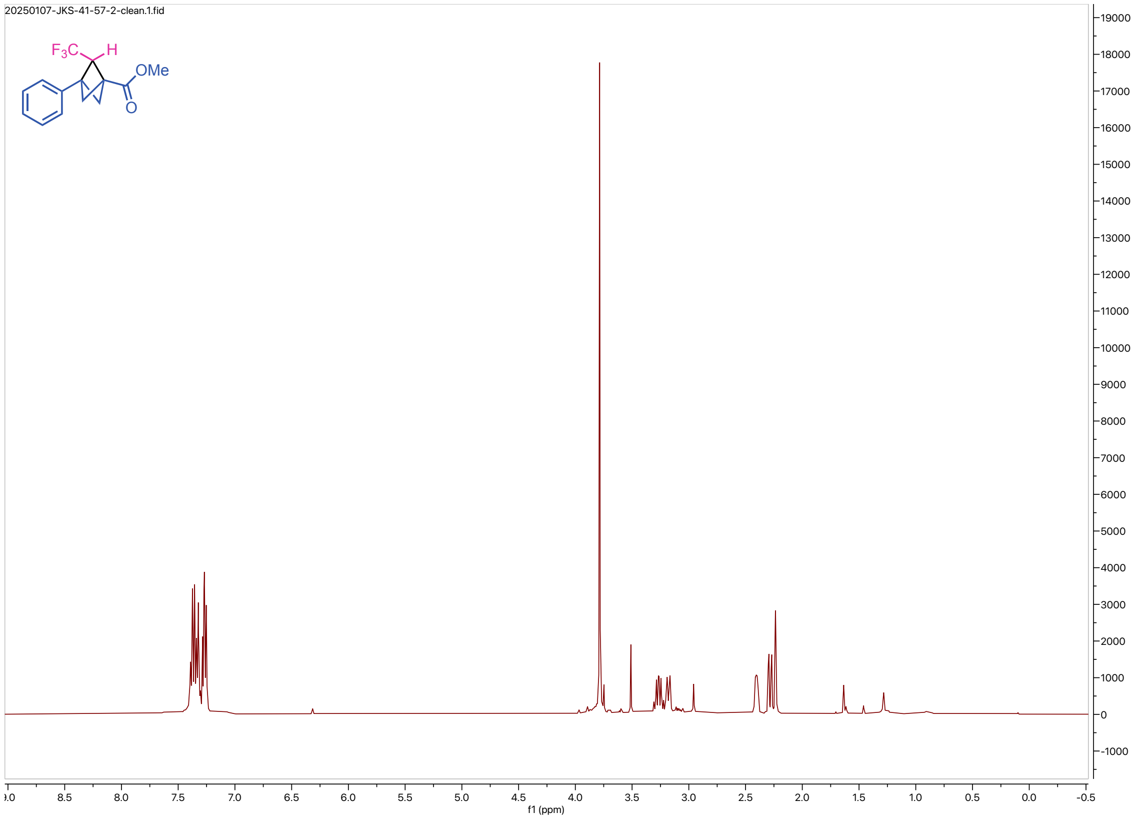

20250107-JKS-41-57-2-clean.1.fid
F3C
H
OMe
O
f1 (ppm)

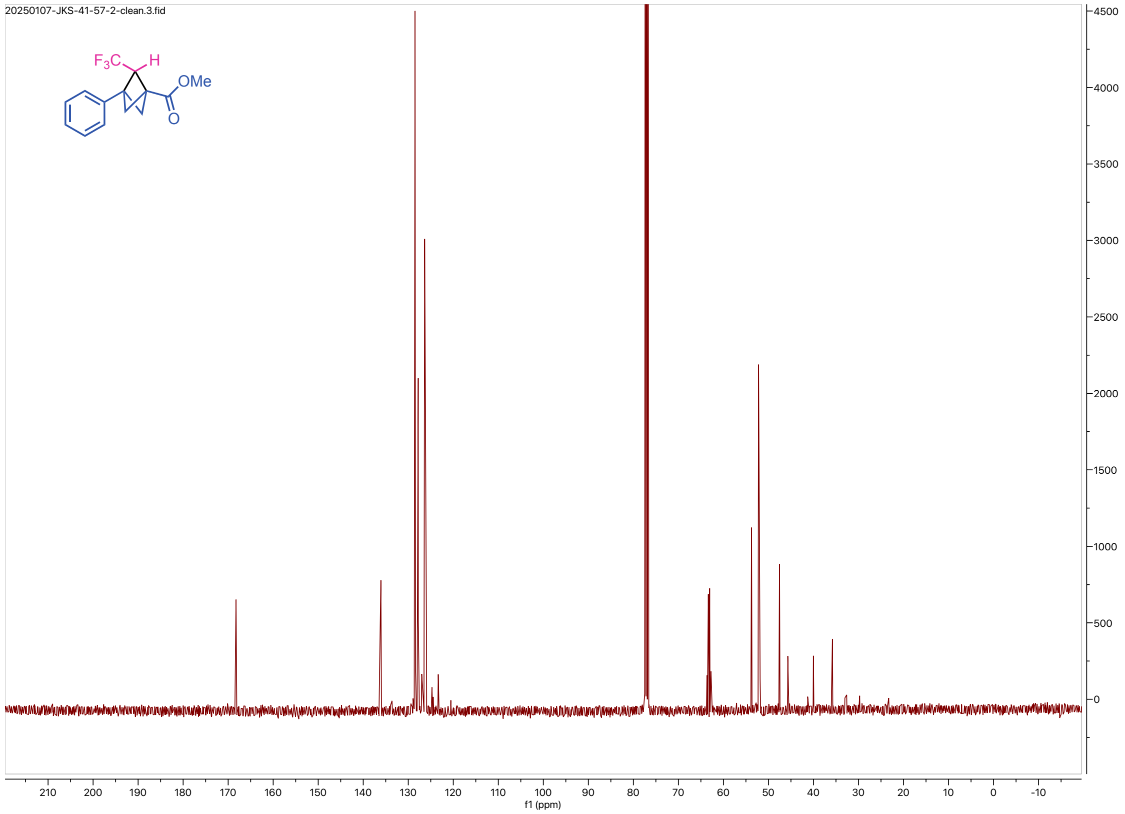

20250107-JKS-41-57-2-clean.3.fid
F3C
H
OMe
O
f1 (ppm)

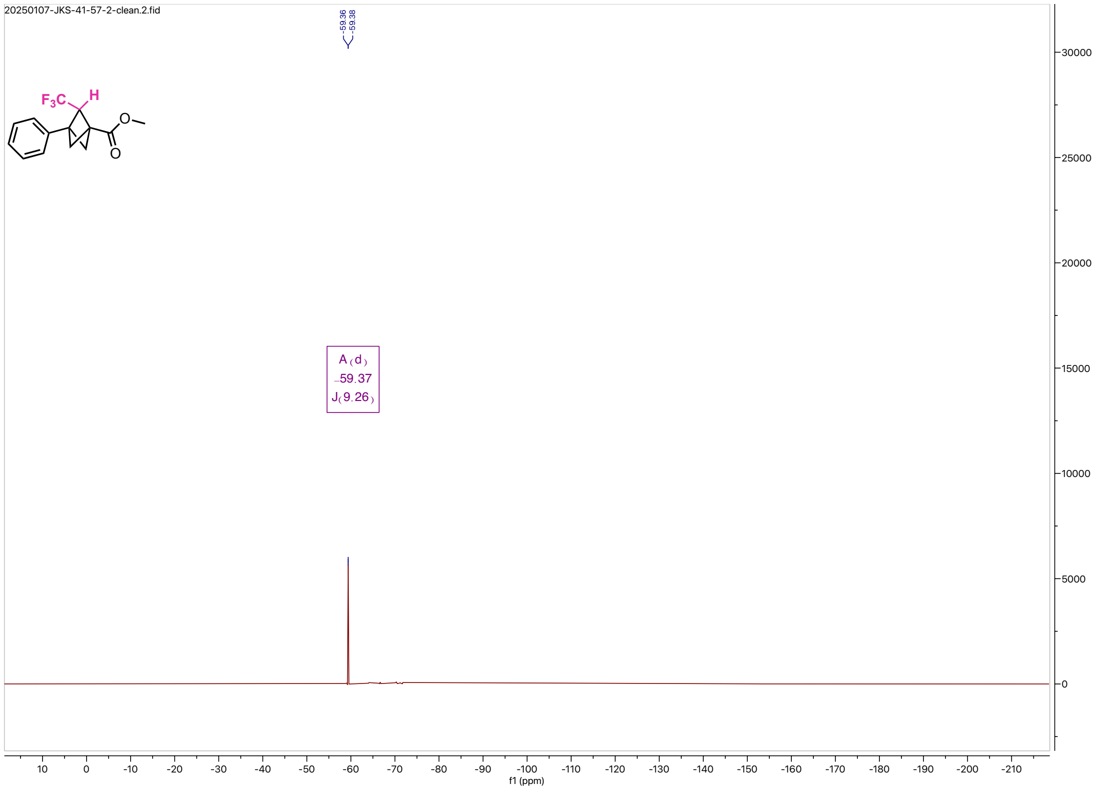
20250107-JKS-41-57-2-clean.2.fid
-59.36
-59.38
F3C
H
O
O
A (d)
-59.37
J (9.26)
f1 (ppm)

Compound **23**

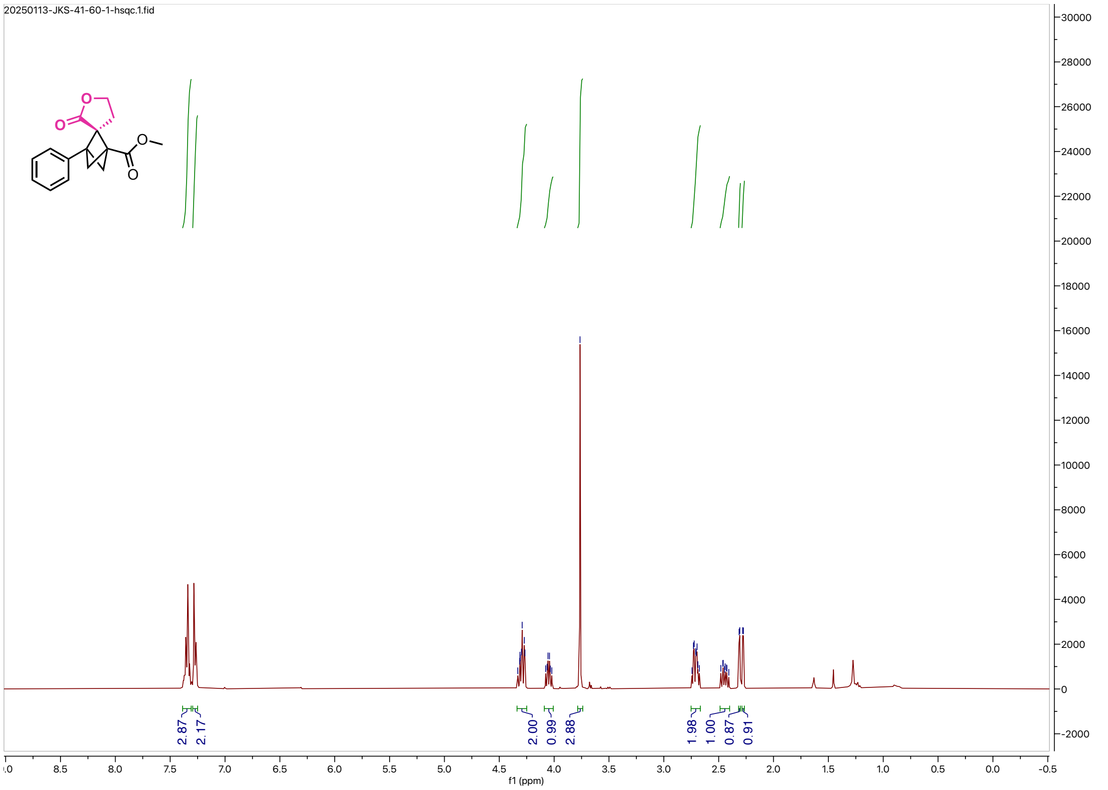
20250113-JKS-41-60-1-hsqc.1.fid
2.87
2.17
2.00
0.99
2.88
1.98
1.00
0.87
0.91
f1 (ppm)

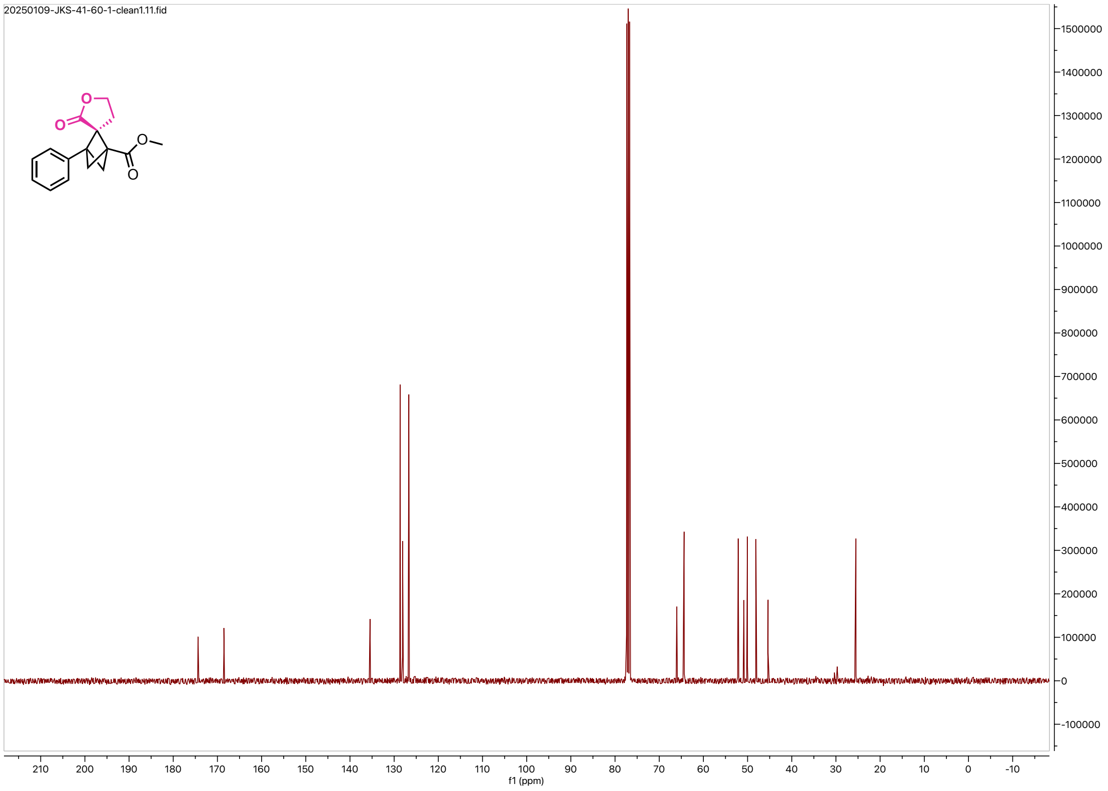
20250109-JKS-41-60-1-clean1.11.fid
f1 (ppm)

Compound **24**

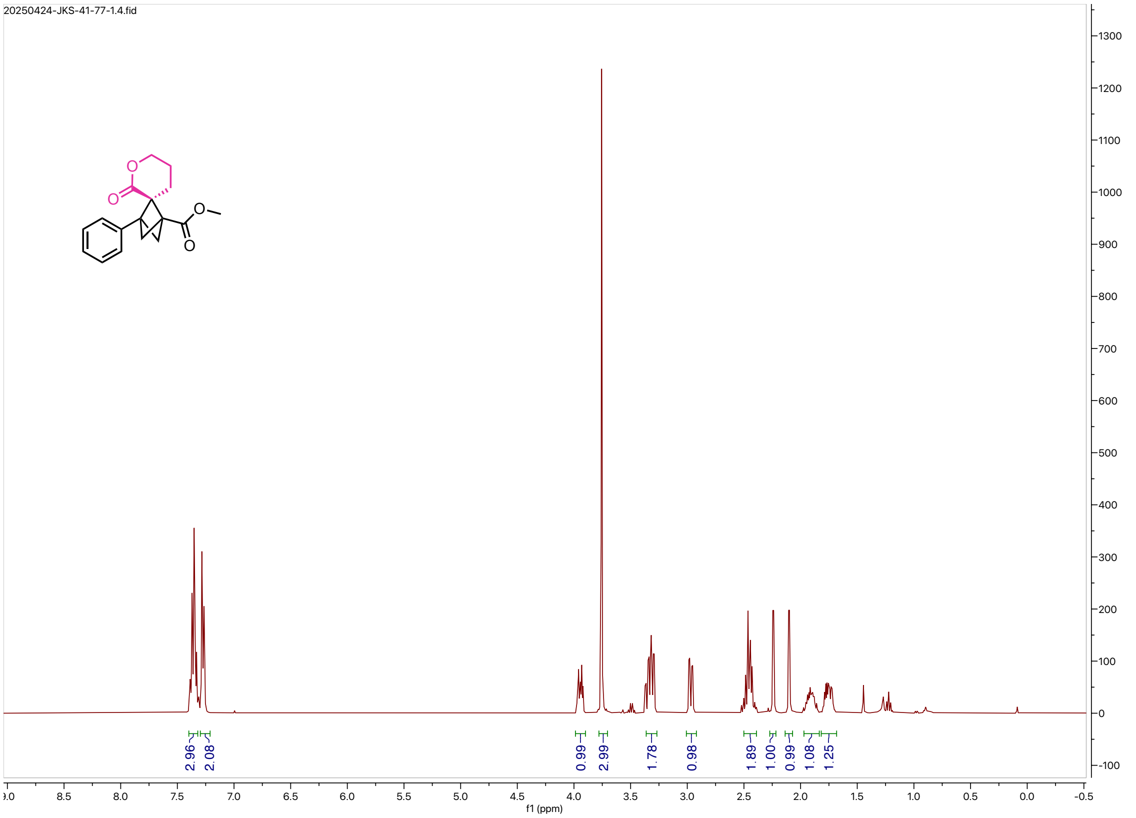

20250424-JKS-41-77-1.4.fid
f1 (ppm)

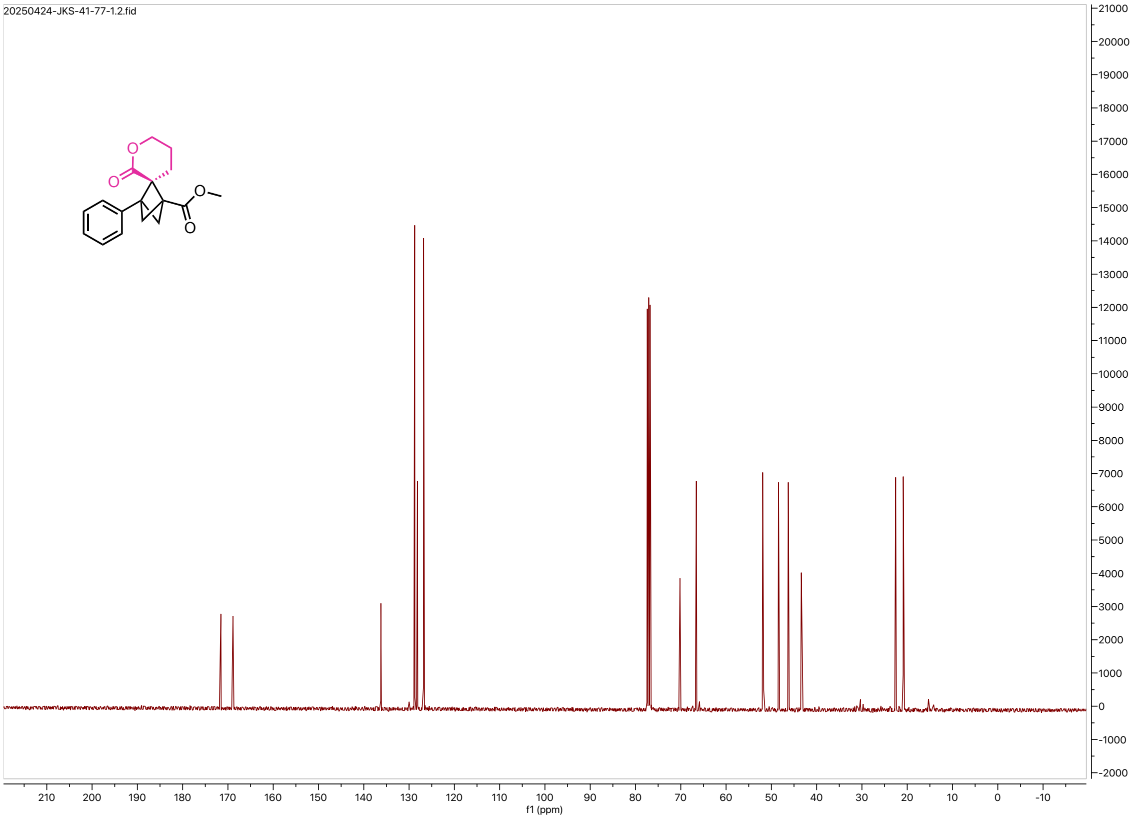

20250424-JKS-41-77-1.2.fid
f1 (ppm)

Compound **25**

20250630-DL-JKS-41-49-01-Clean.42.fid

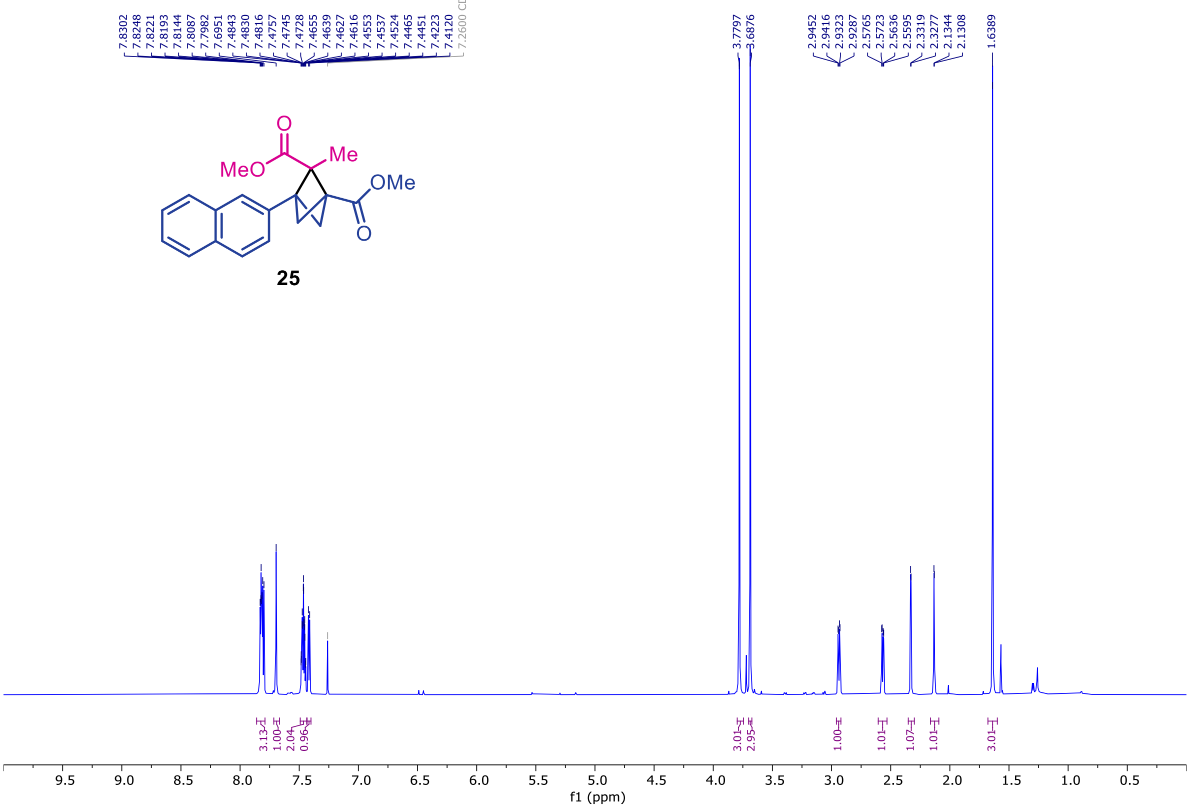

20250630-DL-JKS-41-49-01-Clean.43.fid

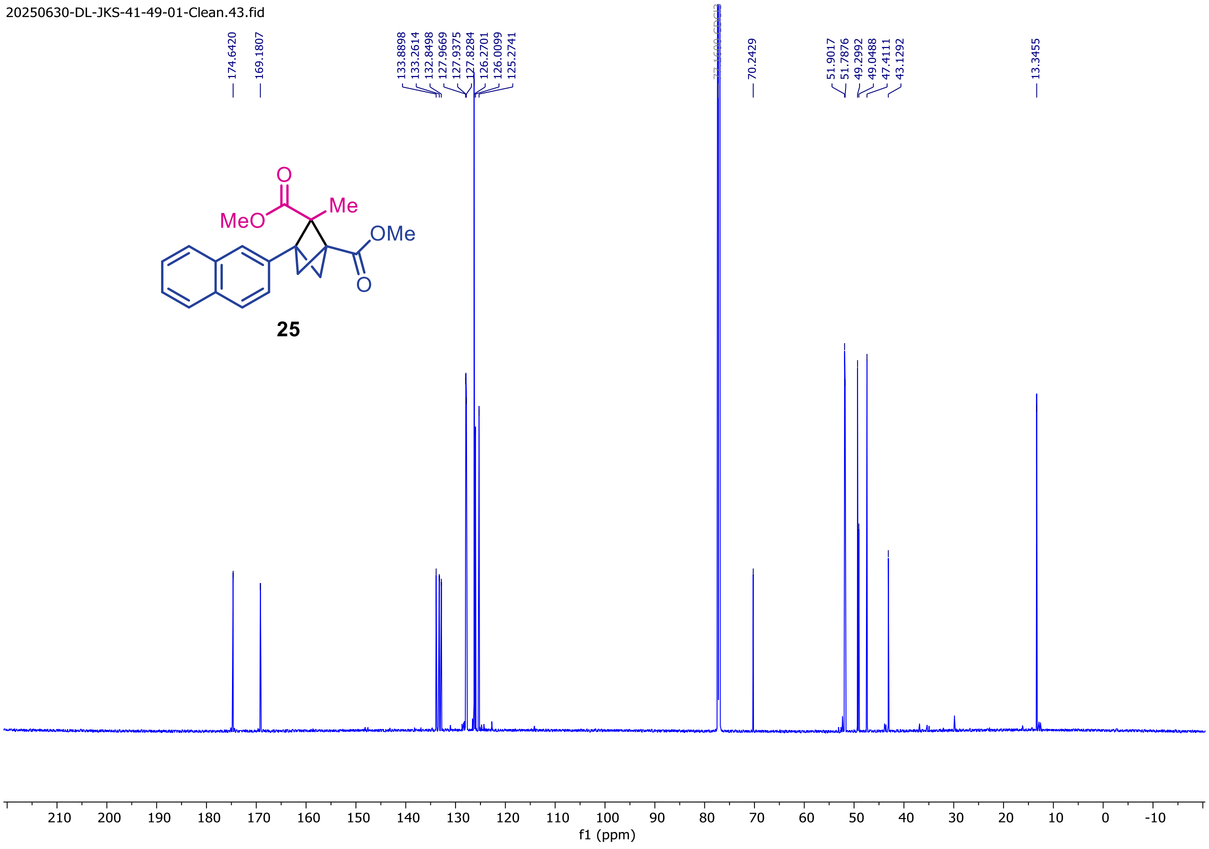

Compound **26**

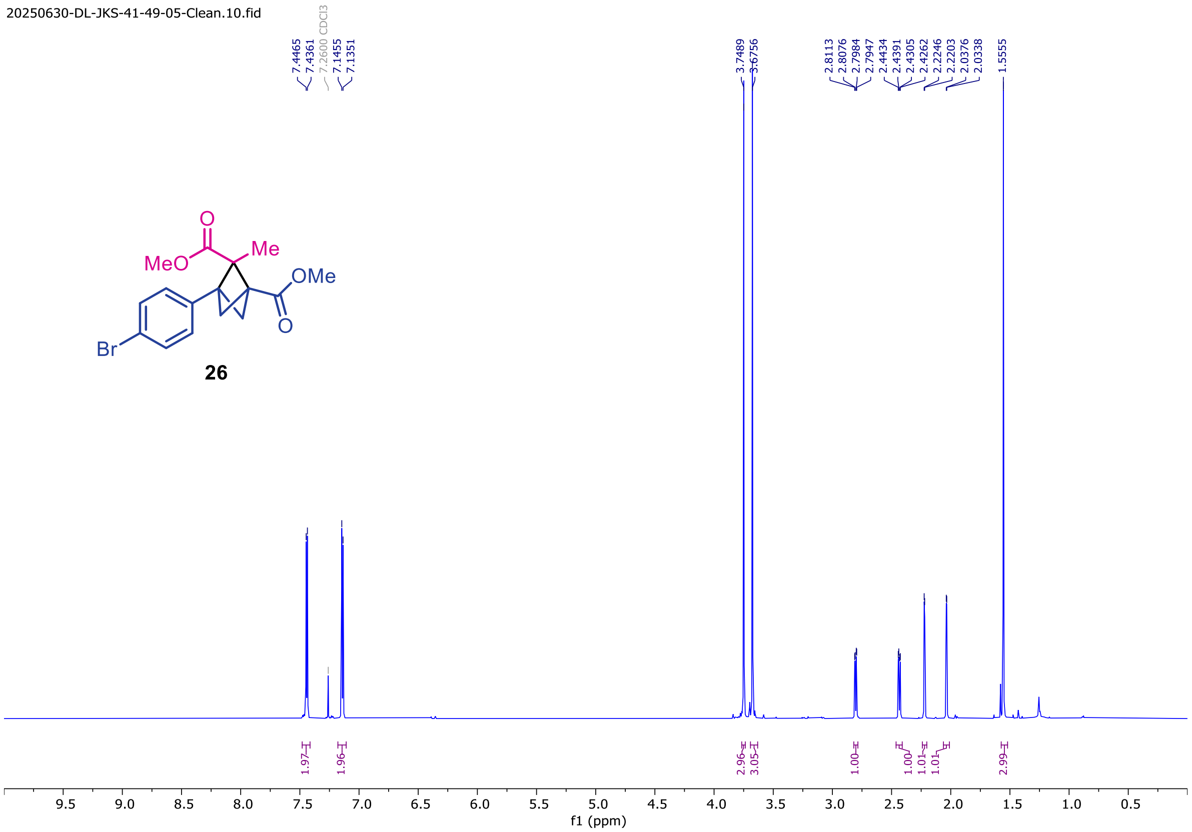

20250630-DL-JKS-41-49-05-Clean.10.fid
7.4465
7.4361
7.2600 CDCl3
7.1455
7.1351
3.7489
3.6756
2.8113
2.8076
2.7984
2.7947
2.4434
2.4391
2.4305
2.4262
2.2246
2.2203
2.0376
2.0338
1.5555
MeO
O
Me
OMe
O
Br
26
1.97
1.96
2.96
3.05
1.00
1.00
1.01
1.01
2.99
f1 (ppm)

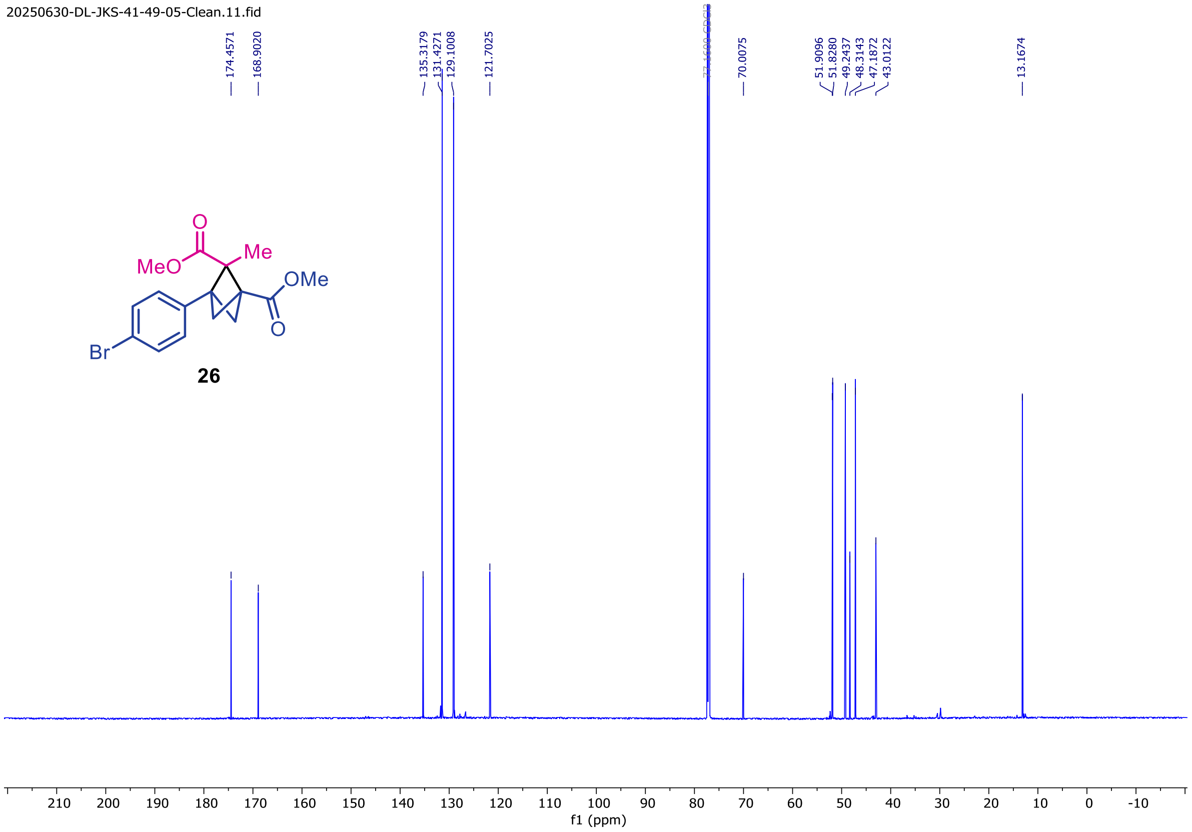

20250630-DL-JKS-41-49-05-Clean.11.fid
174.4571
168.9020
135.3179
131.4271
129.1008
121.7025
70.0075
51.9096
51.8280
49.2437
48.3143
47.1872
43.0122
13.1674
MeO
O
Me
OMe
O
Br
26
f1 (ppm)

Compound **27**

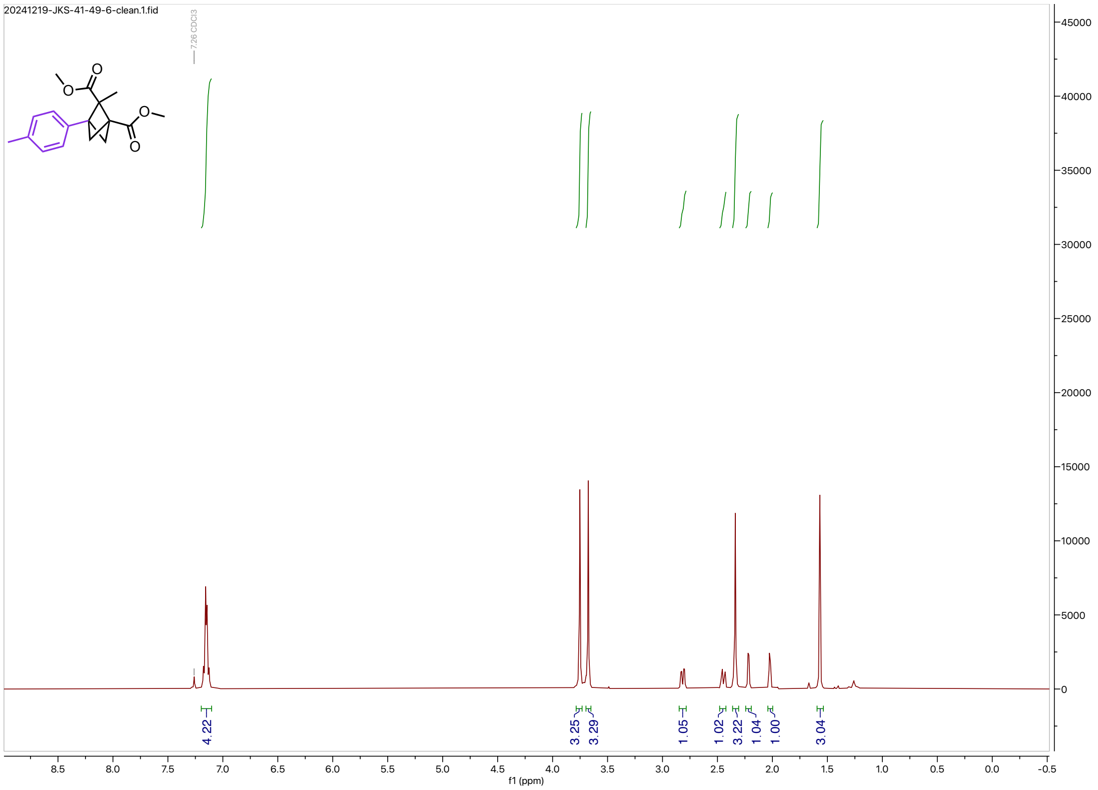
20241219-JKS-41-49-6-clean.1.fid
7.26 CDCl3
4.22
3.25
3.29
1.05
1.02
3.22
1.04
1.00
3.04
f1 (ppm)

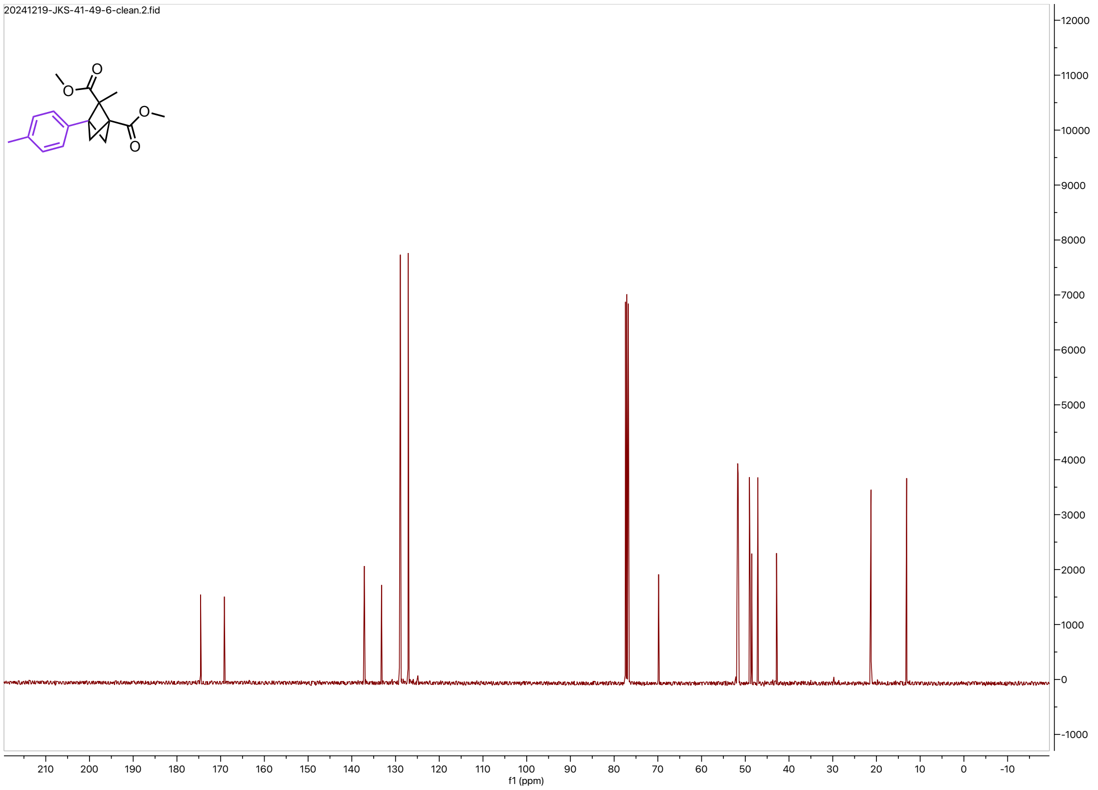
20241219-JKS-41-49-6-clean.2.fid
f1 (ppm)

Compound **28**

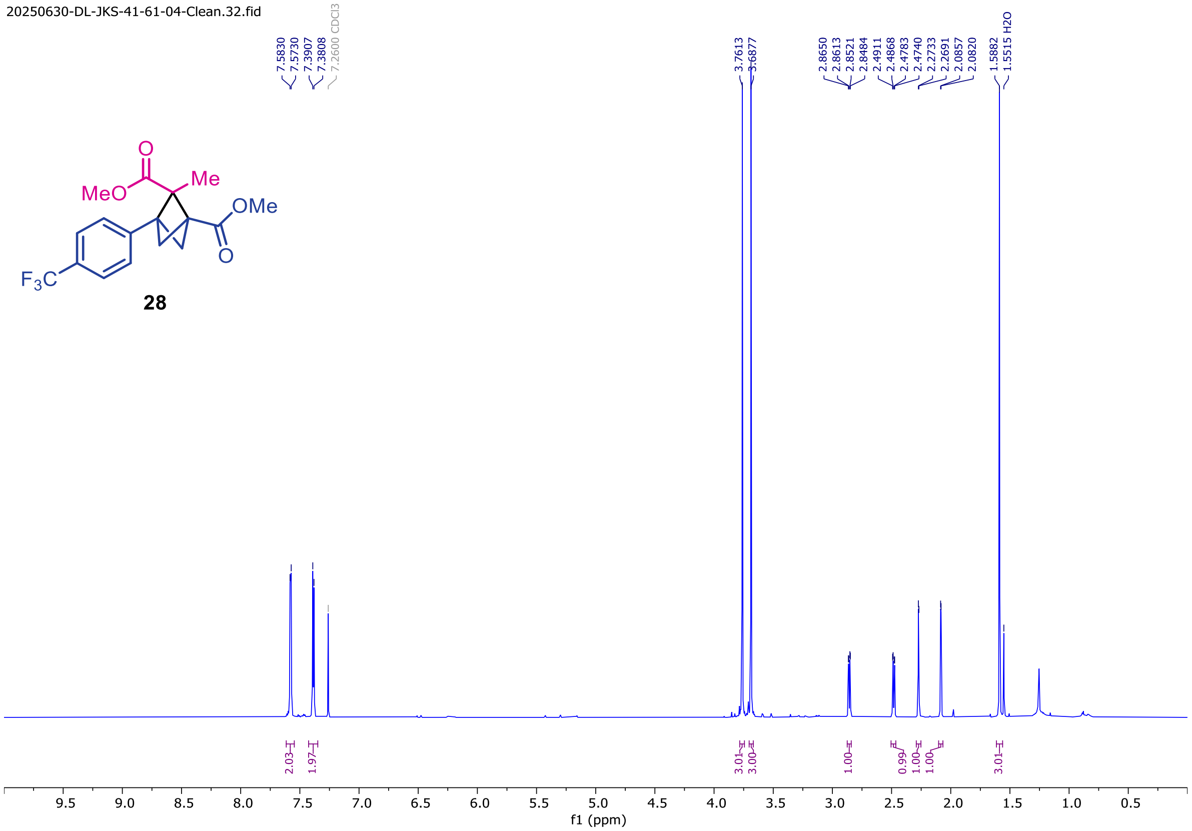
20250630-DL-JKS-41-61-04-Clean.32.fid
7.5830
7.5730
7.3907
7.3808
7.2600 CDCl3
3.7613
3.6877
2.8650
2.8613
2.8521
2.8484
2.4911
2.4868
2.4783
2.4740
2.2733
2.2691
2.0857
2.0820
1.5882
1.5515 H2O
MeO
O
Me
OMe
O
F3C
28
2.03
1.97
3.01
3.00
1.00
0.99
1.00
1.00
3.01
f1 (ppm)

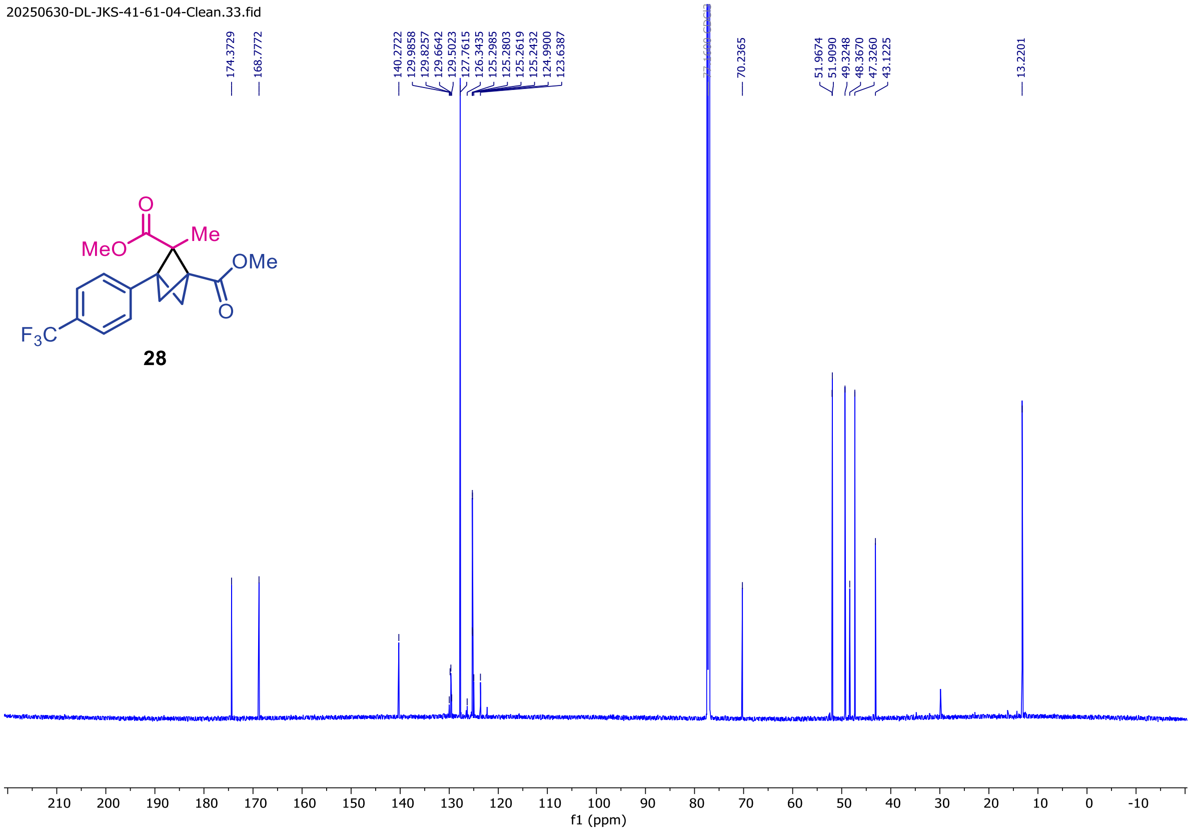
20250630-DL-JKS-41-61-04-Clean.33.fid
174.3729
168.7772
140.2722
129.9858
129.8257
129.6642
129.5023
127.7615
126.3435
125.2985
125.2803
125.2619
125.2432
124.9900
123.6387
70.2365
51.9674
51.9090
49.3248
48.3670
47.3260
43.1225
13.2201
MeO
O
Me
OMe
O
F3C
28
f1 (ppm)

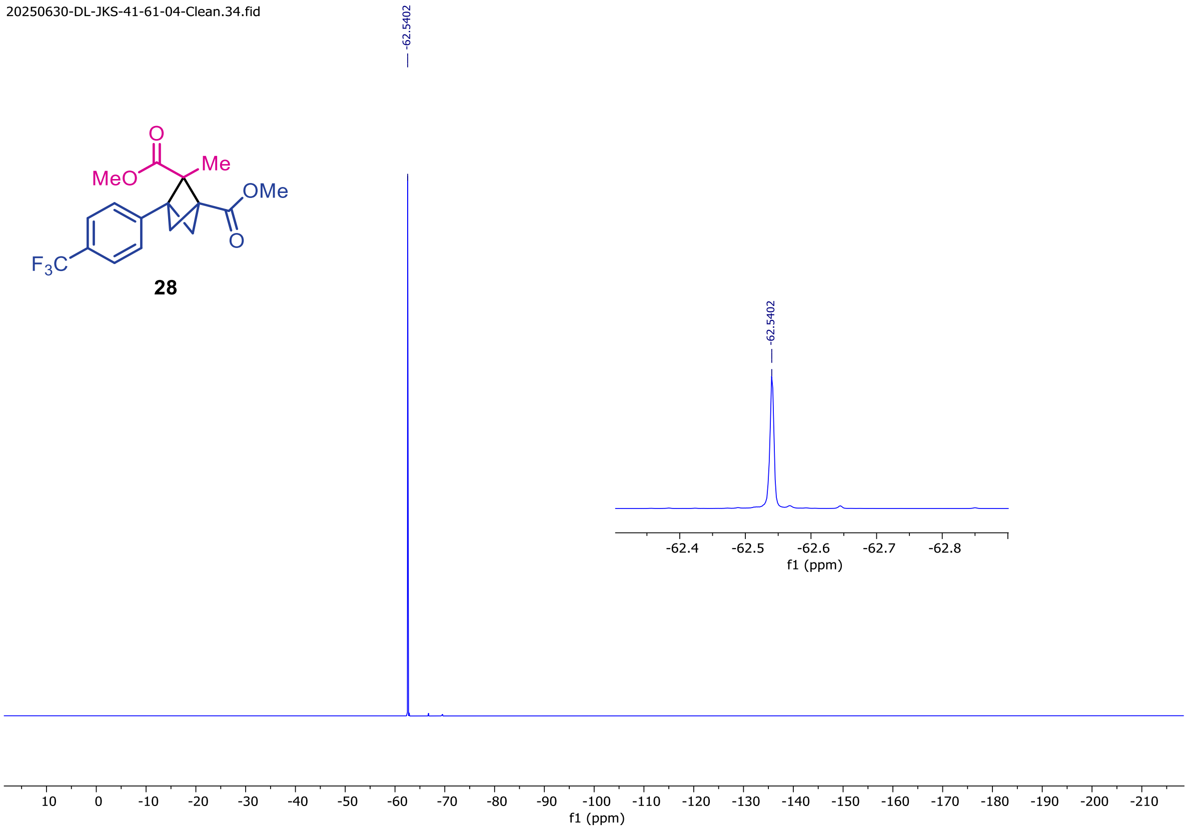

20250630-DL-JKS-41-61-04-Clean.34.fid
-62.5402
MeO
O
Me
OMe
O
F3C
28
-62.5402
-62.4
-62.5
-62.6
-62.7
-62.8
f1 (ppm)
10
0
-10
-20
-30
-40
-50
-60
-70
-80
-90
-100
-110
-120
-130
-140
-150
-160
-170
-180
-190
-200
-210
f1 (ppm)

Compound **29**

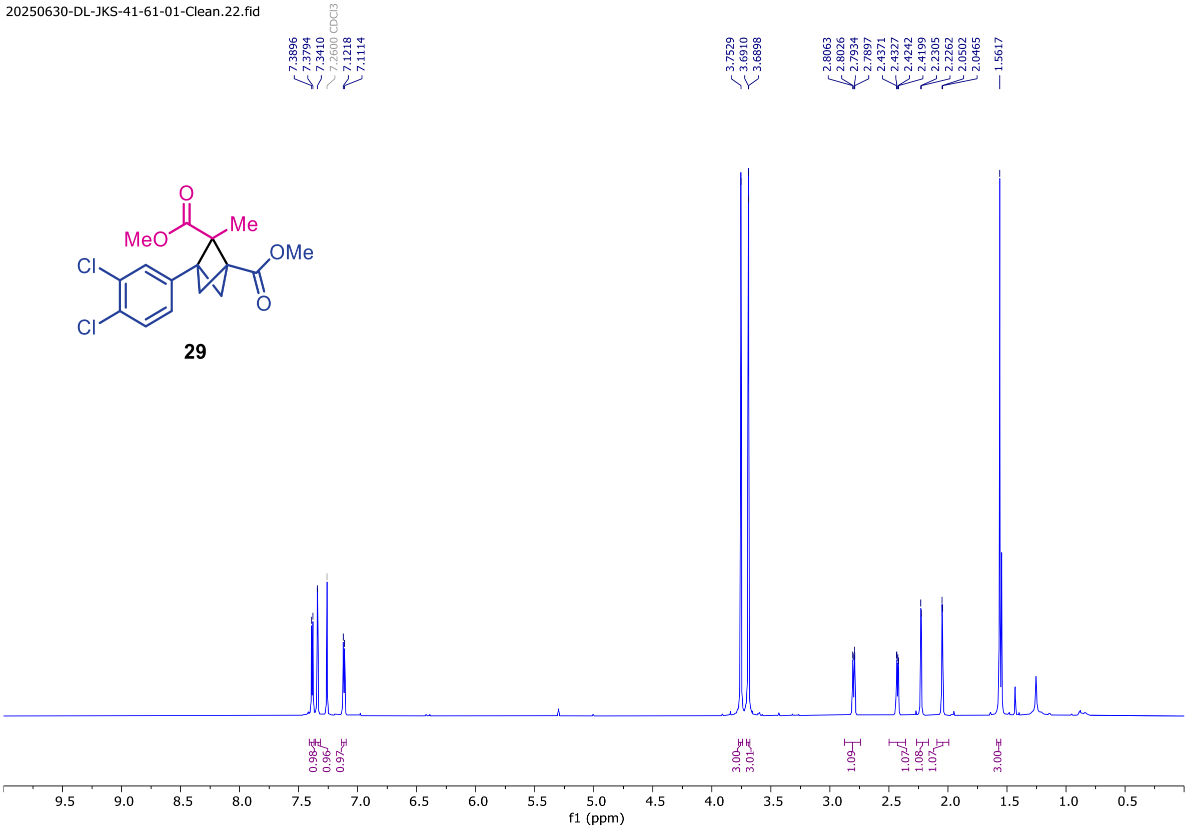
20250630-DL-JKS-41-61-01-Clean.22.fid
7.3896
7.3794
7.3410
7.2600 CDCl3
7.1218
7.1114
3.7529
3.6910
3.6898
2.8063
2.8026
2.7934
2.7897
2.4371
2.4327
2.4242
2.4199
2.2305
2.2262
2.0502
2.0465
1.5617
MeO
O
Me
OMe
O
Cl
Cl
29
0.98
0.96
0.97
3.00
3.01
1.09
1.07
1.08
1.07
3.00
f1 (ppm)

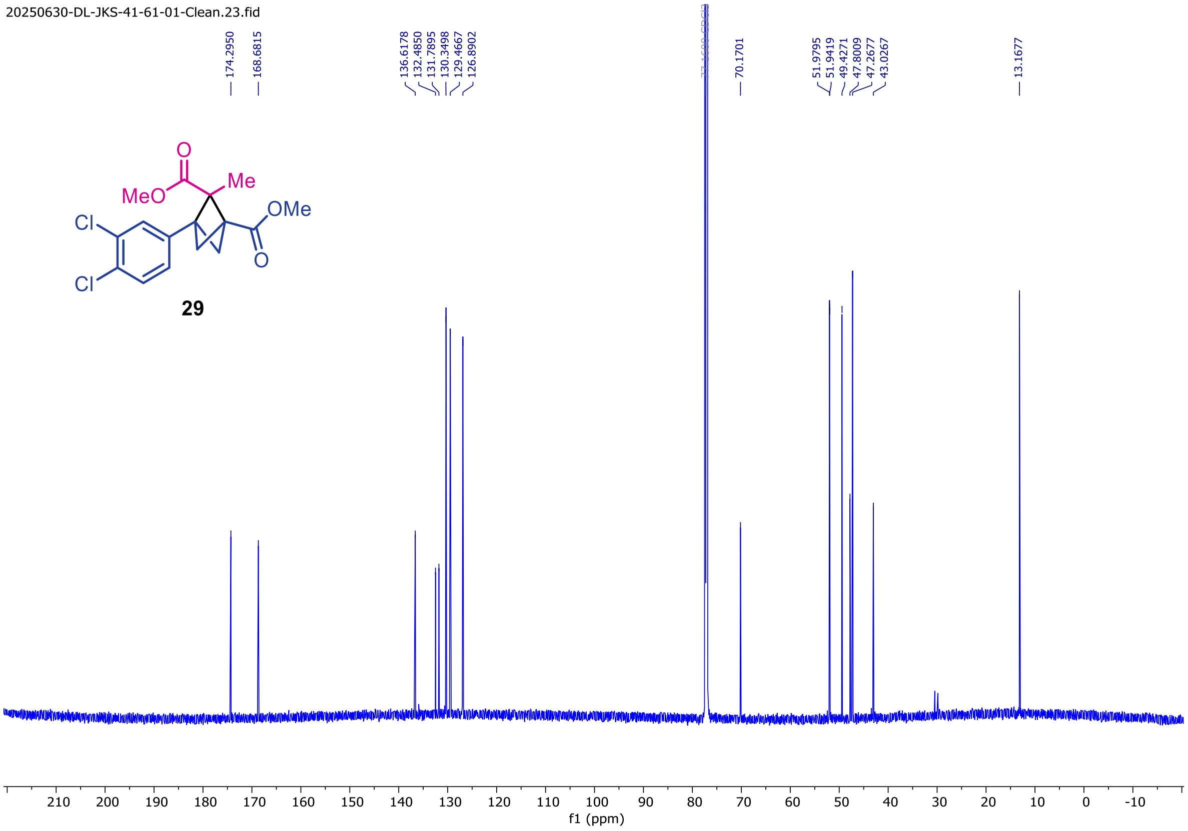
20250630-DL-JKS-41-61-01-Clean.23.fid
174.2950
168.6815
136.6178
132.4850
131.7895
130.3498
129.4667
126.8902
70.1701
51.9795
51.9419
49.4271
47.8009
47.2677
43.0267
13.1677
MeO
O
Me
OMe
O
Cl
Cl
29
f1 (ppm)

Compound **30**

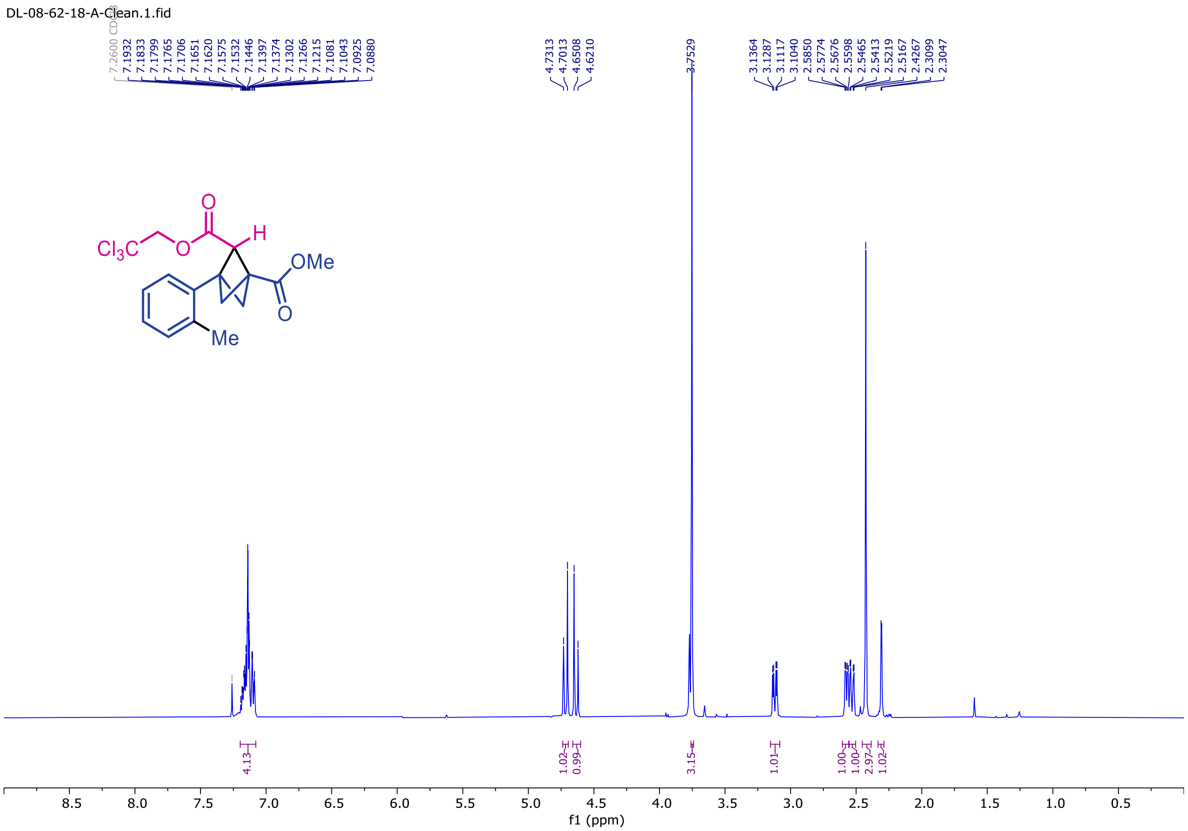
DL-08-62-18-A-Clean.1.fid
7.2600 CDCl3
7.1932
7.1833
7.1799
7.1765
7.1706
7.1651
7.1620
7.1575
7.1532
7.1446
7.1397
7.1374
7.1302
7.1266
7.1215
7.1081
7.1043
7.0925
7.0880
4.7313
4.7013
4.6508
4.6210
3.7529
3.1364
3.1287
3.1117
3.1040
2.5850
2.5774
2.5676
2.5598
2.5465
2.5413
2.5219
2.5167
2.4267
2.3099
2.3047
Cl3C
O
O
H
OMe
O
Me
4.13
1.02
0.99
3.15
1.01
1.00
1.00
2.97
1.02
8.5 8.0 7.5 7.0 6.5 6.0 5.5 5.0 4.5 4.0 3.5 3.0 2.5 2.0 1.5 1.0 0.5
f1 (ppm)

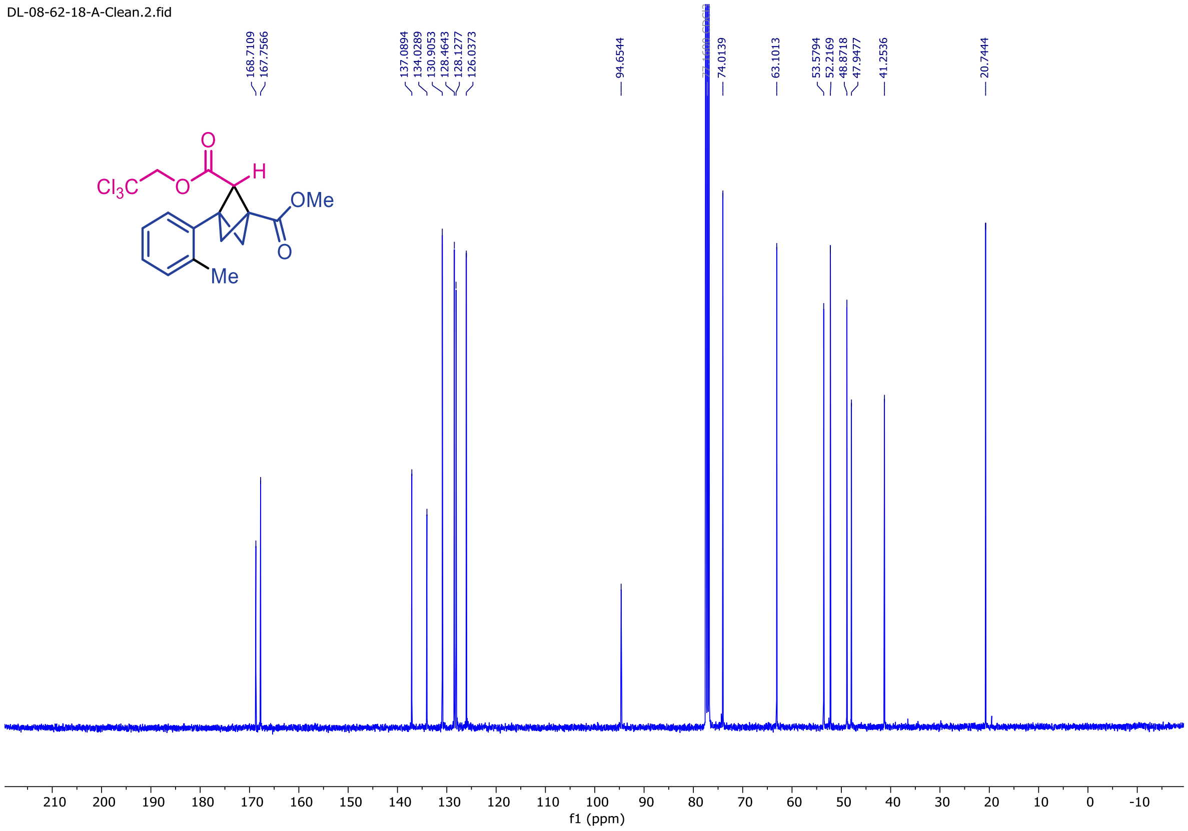
DL-08-62-18-A-Clean.2.fid
168.7109
167.7566
137.0894
134.0289
130.9053
128.4643
128.1277
126.0373
94.6544
74.0139
63.1013
53.5794
52.2169
48.8718
47.9477
41.2536
20.7444
Cl3C
O
O
H
OMe
O
Me
210 200 190 180 170 160 150 140 130 120 110 100 90 80 70 60 50 40 30 20 10 0 -10
f1 (ppm)

Compound **33**

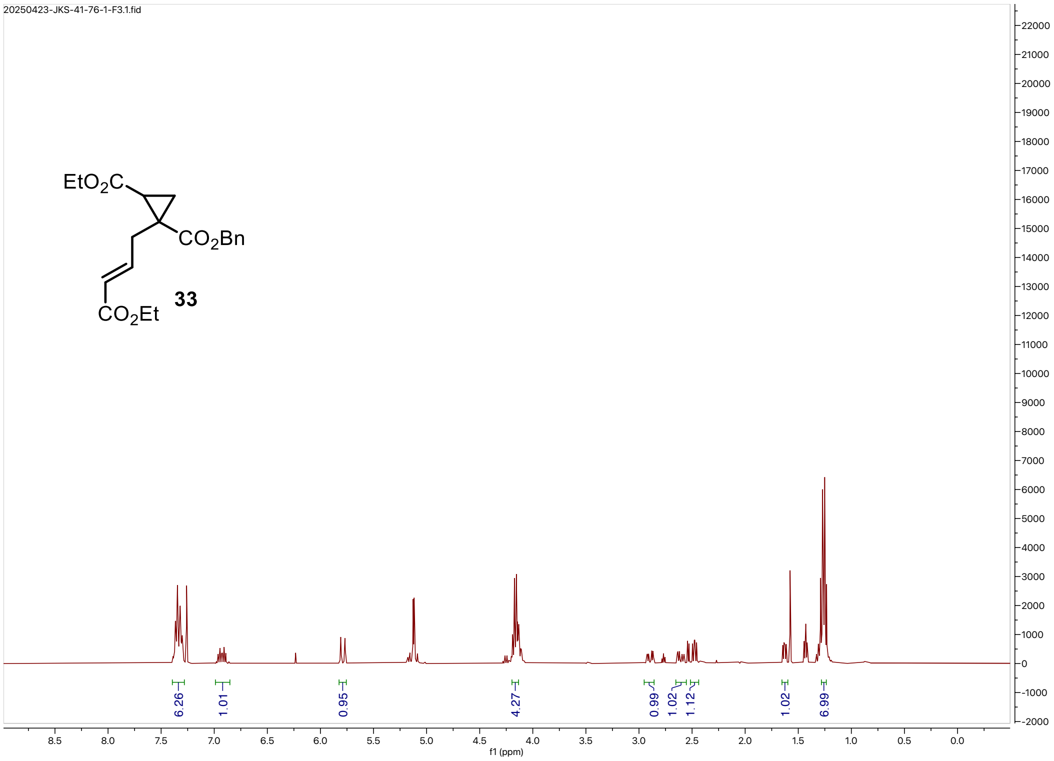
20250423-JKS-41-76-1-F3.1.fid
EtO2C
CO2Bn
CO2Et
33
6.26
1.01
0.95
4.27
0.99
1.02
1.12
1.02
6.99
f1 (ppm)

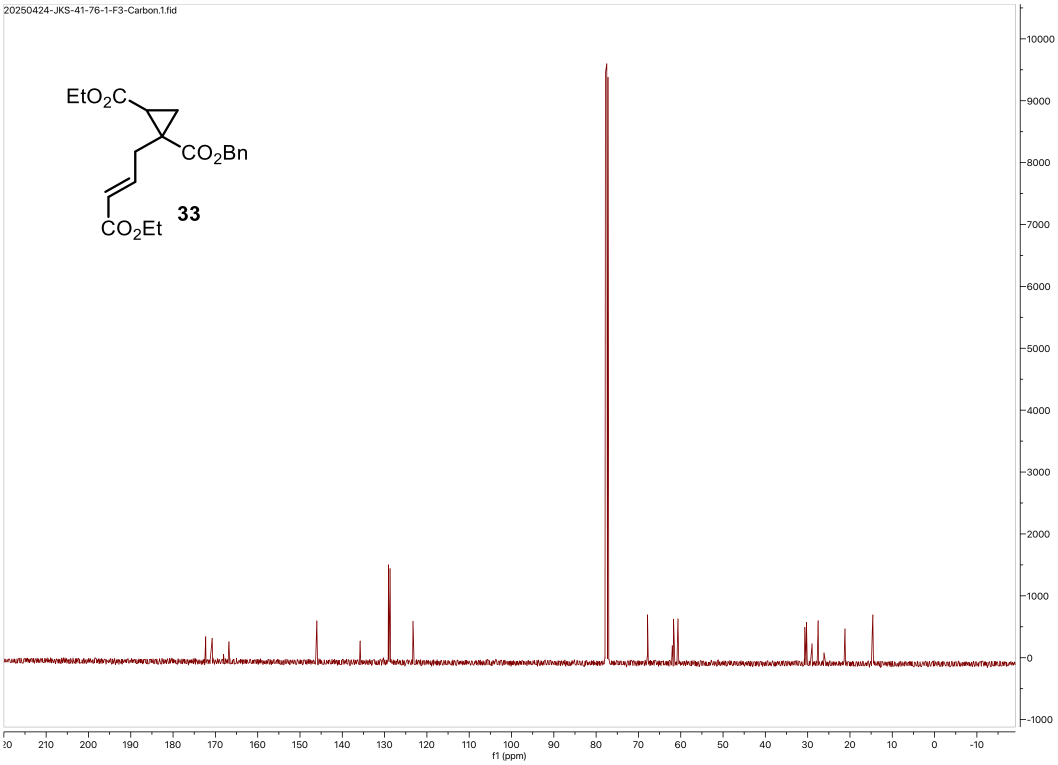
20250424-JKS-41-76-1-F3-Carbon.1.fid
EtO2C
CO2Bn
CO2Et
33
f1 (ppm)

Compound **34**

DL-08-62-07-B-Clean.1.fid

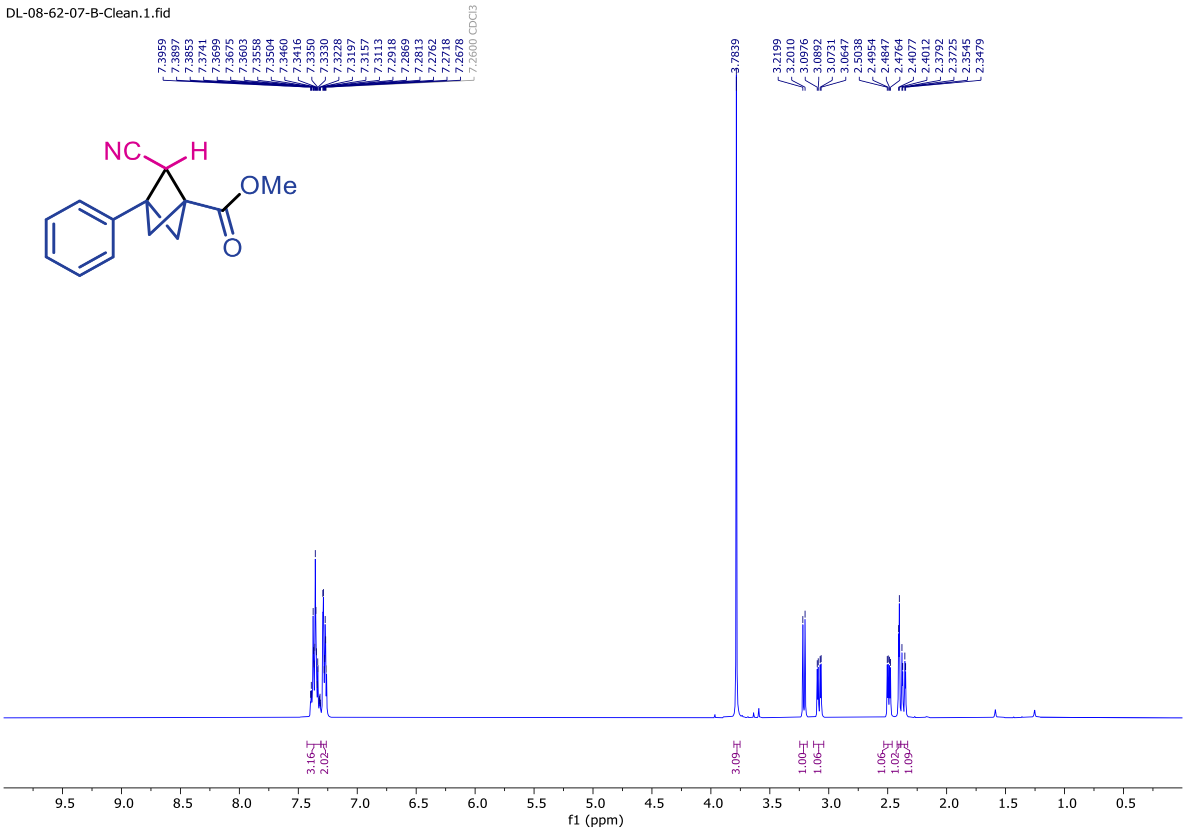

DL-08-62-07-B-Clean.2.fid

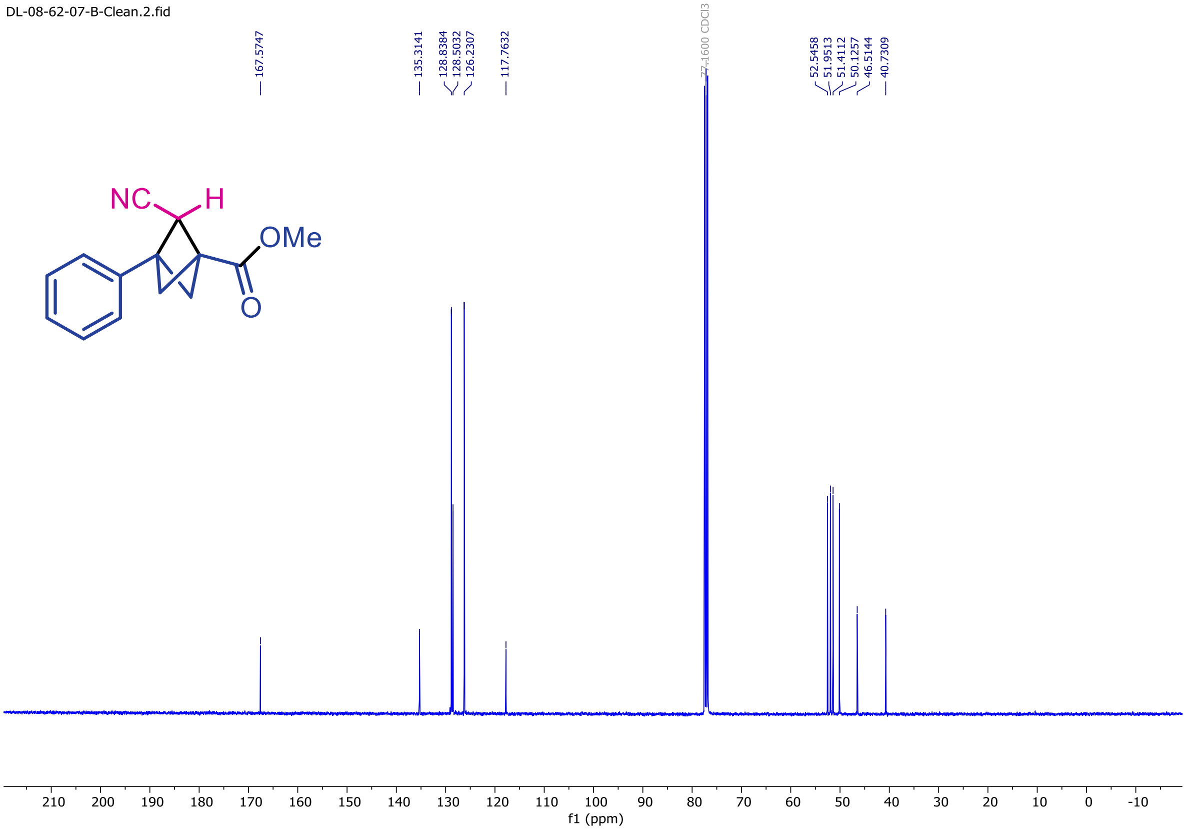

Compound **35**

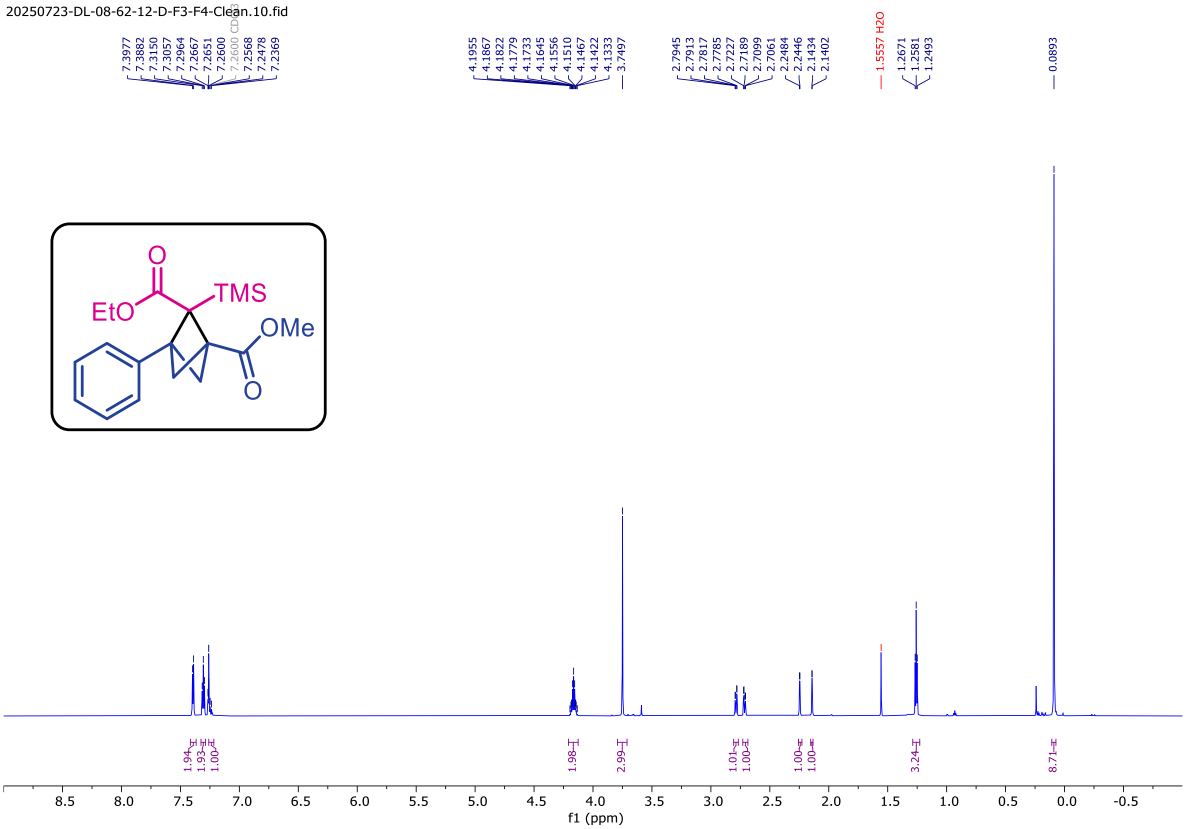

20250723-DL-08-62-12-D-F3-F4-Clean.10.fid
7.3977
7.3882
7.3150
7.3057
7.2964
7.2667
7.2651
7.2600 CDCl3
7.2568
7.2478
7.2369
4.1955
4.1867
4.1822
4.1779
4.1733
4.1645
4.1556
4.1510
4.1467
4.1422
4.1333
3.7497
2.7945
2.7913
2.7817
2.7785
2.7227
2.7189
2.7099
2.7061
2.2484
2.2446
2.1434
2.1402
1.5557 H2O
1.2671
1.2561
1.2493
0.0893
EtO
TMS
OMe
O
1.94
1.93
1.00
1.98
2.99
1.01
1.00
1.00
1.00
3.24
8.71
f1 (ppm)

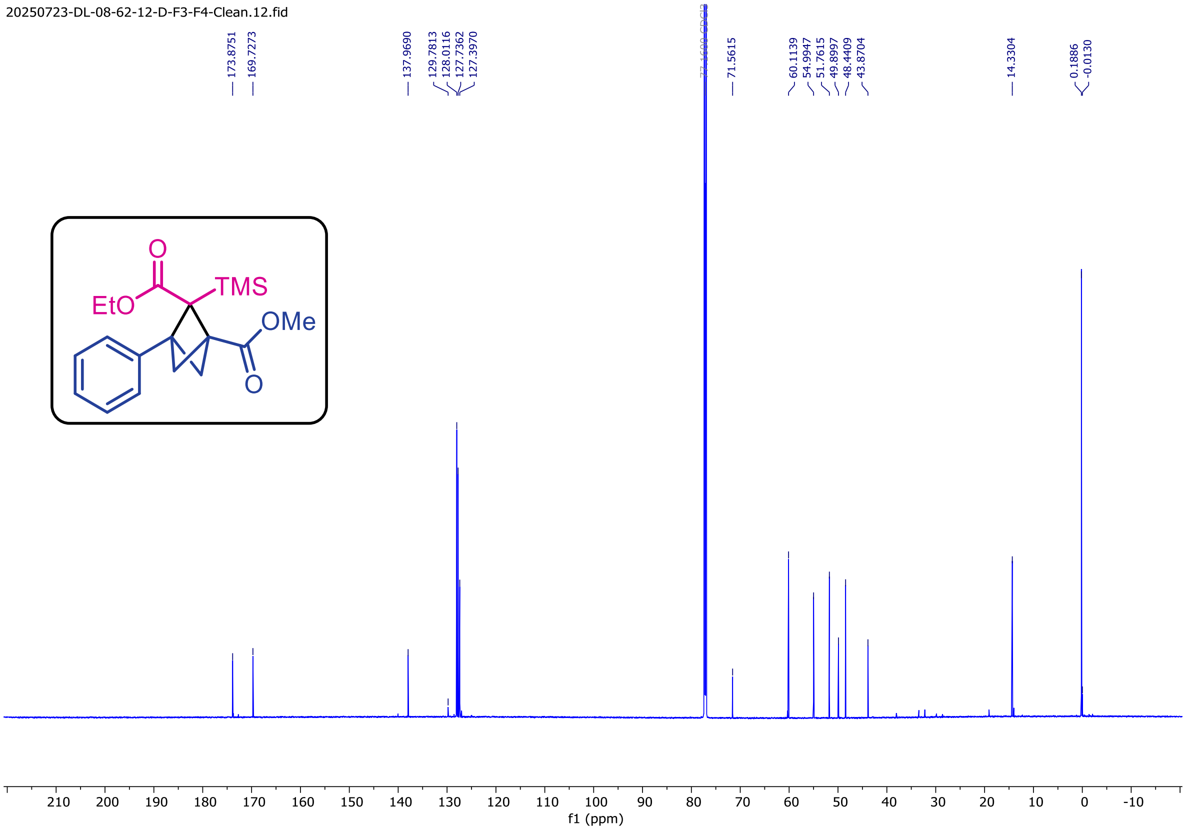

20250723-DL-08-62-12-D-F3-F4-Clean.12.fid
173.8751
169.7273
137.9690
129.7813
128.0116
127.7362
127.3970
71.5615
60.1139
54.9947
51.7615
49.8997
48.4409
43.8704
14.3304
0.1886
-0.0130
EtO
TMS
OMe
O
f1 (ppm)

8. DFT computational study

All calculations were performed using Gaussian-16 suite of programs.[1] Images of 3D structures were rendered using VMD[2] and CYLView20[3]. Geometry and vibrational frequencies of the presented structures were calculated at the (U)ωB97X-D[4] density functional with def2SVP[5] basis sets in conjunction with solvent effect using CPCM[6] solvation model and CH_2Cl_2 as a solvent. To obtain more accurate electronic energy, a single point (i.e. at the [(U)ωB97X-D]/def2SVP optimized geometries) energy was calculated with (U)ωB97X-D density functional, def2TZVPP[5] basis sets, and CPCM(CH_2Cl_2) solvation model. The nature of the located intermediates and transition states were confirmed by vibrational frequency analysis at the same level of the employed approaches for geometry optimization, showing zero and one imaginary frequencies respectively. To confirm that all located saddle points correspond to relevant transformations, Intrinsic Reaction Coordinate (IRC) calculations[7, 8] were performed with the previously mentioned geometry optimization method. Gibbs free energy and Zero-point energy corrections were calculated at a temperature and pressure corresponding to standard reaction conditions (i.e. at the 208.15K (-65 °C) and 1 atm, respectively). For broken symmetry calculations (open-shell singlets), the Stable=opt and guess=mix keywords were added to all DFT calculations.

To validate above selected computational approaches, we conducted a benchmark study of the used computational methods by performing single point energy calculations (i.e. at the [(U)ωB97X-D]/def2TVP calculated geometries) of all reported important intermediates and transition states. (**Figure S1**) The presented benchmark studies include the M06L[9], PBE0[10]-D3BJ[11-13], TPSSh[14], B3LYP[15, 16]-D3BJ, M06[17], M06-2X[17], ωB97X-D, and MN15[18] density functionals with the def2tzvpp basis sets and the CPCM(CH_2Cl_2) solvation model. We also conducted the evaluation of basis sets selection by performing calculations with the 6-311g(d,p) split-level basis sets. All the used computational methods: (a) predicted that **^{1}TS2** and **^{3}TS1a** are energetically lower than **^{1}TS3** and **^{3}TS1b**, respectively, (**Table S1**, entries 1-8) and (b) successfully described the diradical nature of **^{1}TS2** and **^{1}TS3**. Furthermore, it is shown that the selected basis sets have a minimal impact (about a 1-3 kcal/mol) on the calculated relative energies of transition states **^{3}TS1a**, **^{3}TS2a**, **^{1}TS2**, and **^{1}TS3.**

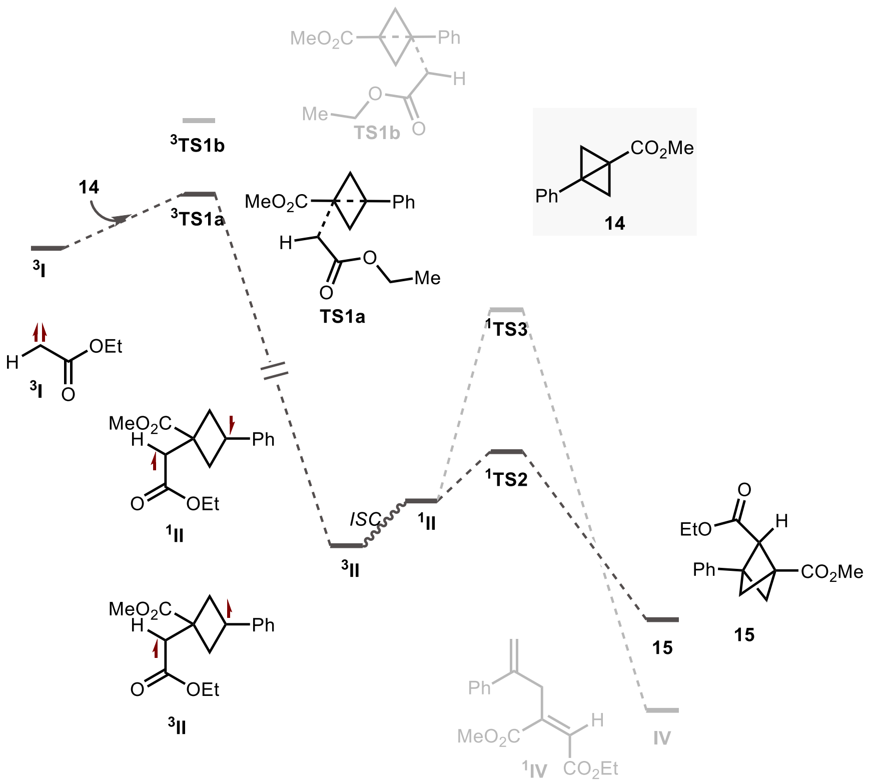

Figure S1. Important intermediates used for Benchmark study

Table S1. Benchmark study for computational method. All reported energies are relative energies to BCB **14** and triplet carbene **³I** (kcal/mol). The <S²> values from DFT calculations for open-shell singlet species

Entry	Theory/basic set	³TS1a	³TS1b	¹II(<S²>)	³II	¹TS2 (<S²>)	¹TS3 (<S²>)
**1**	**[ωB97X-D]/def2TZVPP**	-2.8	-1.9	-67.1 (1.05)	-67.0	-61.1 (0.11)	-54.0 (0.14)
2	M06L/def2TZVPP	-2.4	-0.4	-55.4 (1.03)	-55.4	-53.0 (0.01)	-48.8 (0.01)
3	M06/def2TZVPP	-1.5	-0.1	-58.3 (1.04)	-58.3	-54.0 (0.05)	-49.6 (0.04)
4	M062x/def2TZVPP	-1.7	-0.4	-57.1 (1.03)	-57.1	-51.3 (0.03)	-47.7 (0.01)
5	[PBE0-D3BJ]/def2TZVPP	-4.7	-3.1	-66.9 (0.72)	-66.9	-62.4 (0.06)	-56.4 (0.07)
6	TPSSh/def2TZVPP	1.3	4.0	-55.8 (1.03)	-55.7	-53.8 (0.02)	-48.6 (0.03)
7	[B3LYP-D3BJ]/def2TZVPP	-5.9	-4.4	-68.1 (1.03)	-68.1	-63.8 (0.02)	-61.1 (0.02)
8	MN15/def2TZVPP	-3.5	-2.1	-64.2 (1.04)	-64.1	-61.4 (0.04)	-54.8 (0.02)
9	[ωB97X-D]/[6-311g(d,p)]	-5.8	-4.9	-70.4 (1.05)	-70.3	-64.0 (0.11)	-57.0 (0.15)

Reference

(1) Frisch, M. J.; Trucks, G. W.; Schlegel, H. B.; Scuseria, G. E.; Robb, M. A.; Cheeseman, J. R.; Scalmani, G.; Barone, V.; Petersson, G. A.; Nakatsuji, H.; et al. Gaussian 16 Rev. C.01. **2016**.

(2) Humphrey, W.; Dalke, A.; Schulten, K. VMD: Visual molecular dynamics. *J. Mol. Graph.* **1996**, *14*, 33-38.

(3) Legault, C. Y. CYLview20. **2020**.

(4) Chai, J.-D.; Head-Gordon, M. Long-range corrected hybrid density functionals with damped atom–atom dispersion corrections. *Phys. Chem. Chem. Phys.* **2008**, *10*, 6615-6620, 10.1039/B810189B.

(5) Weigend, F.; Ahlrichs, R. Balanced basis sets of split valence, triple zeta valence and quadruple zeta valence quality for H to Rn: Design and assessment of accuracy. *Phys. Chem. Chem. Phys.* **2005**, *7*, 3297-3305, 10.1039/B508541A.
(6) Cossi, M.; Rega, N.; Scalmani, G.; Barone, V. Energies, structures, and electronic properties of molecules in solution with the C-PCM solvation model. *J. Comput. Chem.* **2003**, *24*, 669-681.
(7) Fukui, K. The path of chemical reactions - the IRC approach. *Acc. Chem. Res.* **1981**, *14*, 363-368.
(8) Fukui, K. Formulation of the reaction coordinate. *J. Phys. Chem.* **1970**, *74*, 4161-4163.
(9) Zhao, Y.; Truhlar, D. G. A new local density functional for main-group thermochemistry, transition metal bonding, thermochemical kinetics, and noncovalent interactions. *J. Chem. Phys.* **2006**, *125*, 194101.
(10) Adamo, C.; Barone, V. Toward reliable density functional methods without adjustable parameters: The PBE0 model. *J. Chem. Phys.* **1999**, *110*, 6158-6170.
(11) Grimme, S. Semiempirical GGA-type density functional constructed with a long-range dispersion correction. *J. Comput. Chem.* **2006**, *27*, 1787-1799.
(12) Becke, A. D.; Johnson, E. R. A density-functional model of the dispersion interaction. *J. Chem. Phys.* **2005**, *123*, 154101.
(13) Becke, A. D.; Johnson, E. R. Exchange-hole dipole moment and the dispersion interaction: High-order dispersion coefficients. *J. Chem. Phys.* **2006**, *124*, 014104.
(14) Tao, J.; Perdew, J. P.; Staroverov, V. N.; Scuseria, G. E. Climbing the Density Functional Ladder: Nonempirical Meta--Generalized Gradient Approximation Designed for Molecules and Solids. *Phys. Rev. Lett.* **2003**, *91*, 146401.
(15) Lee, C.; Yang, W.; Parr, R. G. Development of the Colle-Salvetti correlation-energy formula into a functional of the electron density. *Physical Review B* **1988**, *37*, 785-789.
(16) Becke, A. D. Density-functional exchange-energy approximation with correct asymptotic behavior. *Physical Review A* **1988**, *38*, 3098-3100.
(17) Zhao, Y.; Truhlar, D. G. The M06 suite of density functionals for main group thermochemistry, thermochemical kinetics, noncovalent interactions, excited states, and transition elements: two new functionals and systematic testing of four M06-class functionals and 12 other functionals. *Theor. Chem. Acc.* **2008**, *120*, 215-241.
(18) Yu, H. S.; He, X.; Li, S. L.; Truhlar, D. G. MN15: A Kohn–Sham global-hybrid exchange–correlation density functional with broad accuracy for multi-reference and single-reference systems and noncovalent interactions. *Chem. Sci.* **2016**, *7*, 5032-5051, 10.1039/C6SC00705H.
(19) Wang, Y.; Yuzawa, T.; Hamaguchi, H.-o.; Toscano, J. P. Time-Resolved IR Studies of 2-Naphthyl(carbomethoxy)carbene: Reactivity and Direct Experimental Estimate of the Singlet/Triplet Energy Gap. *J. Am. Chem. Soc.* **1999**, *121*, 2875-2882.
(20) Wang, J.; Burdzinski, G.; Gustafson, T. L.; Platz, M. S. Ultrafast Study of p-Biphenylyldiazoethane. The Chemistry of the Diazo Excited State and the Relaxed Carbene. *J. Am. Chem. Soc.* **2007**, *129*, 2597-2606.
(21) Zhang, Y.; Kubicki, J.; Platz, M. S. Ultrafast UV−Visible and Infrared Spectroscopic Observation of a Singlet Vinylcarbene and the Intramolecular Cyclopropenation Reaction. *J. Am. Chem. Soc.* **2009**, *131*, 13602-13603.
(22) Zhang, Z.; Gevorgyan, V. Visible Light-Induced Reactions of Diazo Compounds and Their Precursors. *Chem. Rev.* **2024**, *124*, 7214-7261.
(23) Kadam, G. A.; Singha, T.; Rawat, S.; Hari, D. P. Rhodium(II)-Catalyzed Strain-Enabled Stereoselective Synthesis of Skipped Dienes. *ACS Catal.* **2024**, *14*, 12225-12233.
(24) Zhang, X.; Tian, T.; Liao, P.; Liu, Z.; Murali, K.; Bi, X. Copper-Catalyzed Cross-Coupling of Bicyclobutanes with Triftosylhydrazone Leading to Skipped Dienes. *Org. Lett.* **2025**, *27*, 2300-2304.

8.1. Decomposition of diazoacetates

The decomposition of diazoacetates in the presence of photocatalyst (**PC**) and under light irradiation is a complicated process.[19-22] Here, at first, we are proposing that it starts with the **S0** to **S1** excitation of **PC** by blue LED and proceeds via the energy transfer from the excited **S1 (PC)** state of **PC** to the ground-state of ethyl diazoacetate to generate an excited singlet diazoacetate **S1 (diazo)** (below, the used diazoacetate is labeled as [**SUB-**

1]) see **Figure S1**). The excited **S1 (diazo)** of diazoacetate quenches to the first excited triplet state **T1** (**diazo**), via the interstate crossing (ISC) mechanism, before the ethyl diazoacetate decomposition (with a barrier of 11.3 kcal/mol) to generate the triplet carbene intermediate **^{3}I** (below, we called this mechanism as the "ISC-then-decomposition" pathway). (**Figure S2**) Alternatively, the triplet carbene intermediate **^{3}I** can also be formed via the ethyl diazoacetate decomposition from its **S1 (diazo)** excited state followed by transformation of the generated singlet carbene to the triplet one via the interstate crossing mechanism (here, we called this the "decomposition-then-ISC" pathway). While the employed DFT approach falls short of exploring the "decomposition-then-ISC" mechanistic scenario, herein, we cautiously discount this pathway because it is expected to require more energy than the previous process for the required N2-exclusion. Regardless of the above-mentioned triplet carbene formation scenarios in the presence of PC and under light irradiation, in the next stage, the formed triplet carbene is involved in the C-C bond activation and drives the completion of the reaction. Another possible mechanism is that the **T1 (PC)** formed via an interstate-crossing from **S1 (PC)** undergoes an dexter energy transfer mechanism with ground state diazo compound **S1 (diazo)** to generate triplet diazo intermediate **T1 (diazo)** which then undergoes N_2 extrusion as shown in **Figure S2**.

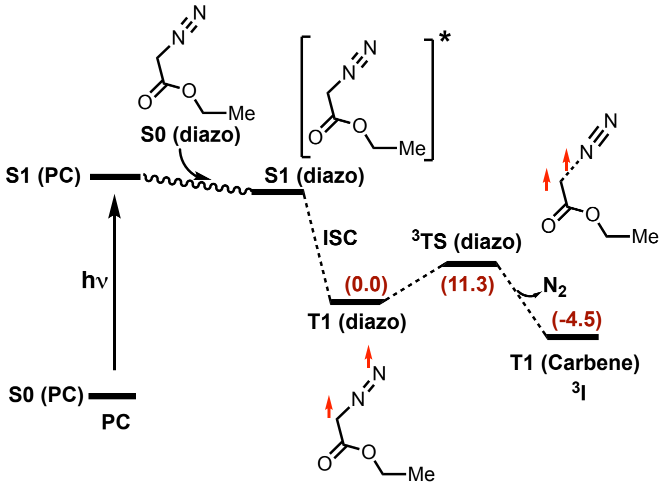

Figure S2. The proposed mechanism of ethyl diazoacetate decomposition in the presence of photo catalyst (**PC**) and under irradiation. Reported energies are Gibbs free energy (in kcal/mol) calculated at the {[(U)ωB97X-D] + CPCM(CH_2Cl_2)}/def2TZVPP// {[(U)ωB97X-D] + CPCM(CH_2Cl_2)}/def2SVP level of theory. For more details and for possible alternative mechanisms see text above.

8.2. Reaction of bicyclobutane with singlet carbene

In order to support our hypothesis that the formed triplet carbene is involved in the C-C bond activation and drives the completion of the reaction we also explored the reactivity of the close-shell singlet (i.e. diamagnetic) carbene **^{1}I**. (**Figure S3A**) We found that the reaction of BCB **14** with a close-shell singlet carbene is a concerted process and leads to an alkene fragmentation product **IV**. (**Figure S3B**) All of our attempts to locate the process leading to the desired BCP product **15** were unsuccessful. These computational findings are consistent with the previous studies shown that reaction of singlet rhodium-carbene[23]

and copper-carbene[24] with BCB leads to an alkene fragmentation, i.e. the product **IV**. Thus, the presented computational results along with literature precedence support hypothesis that only the reaction of triplet carbene with BCB leads to the BCP product.

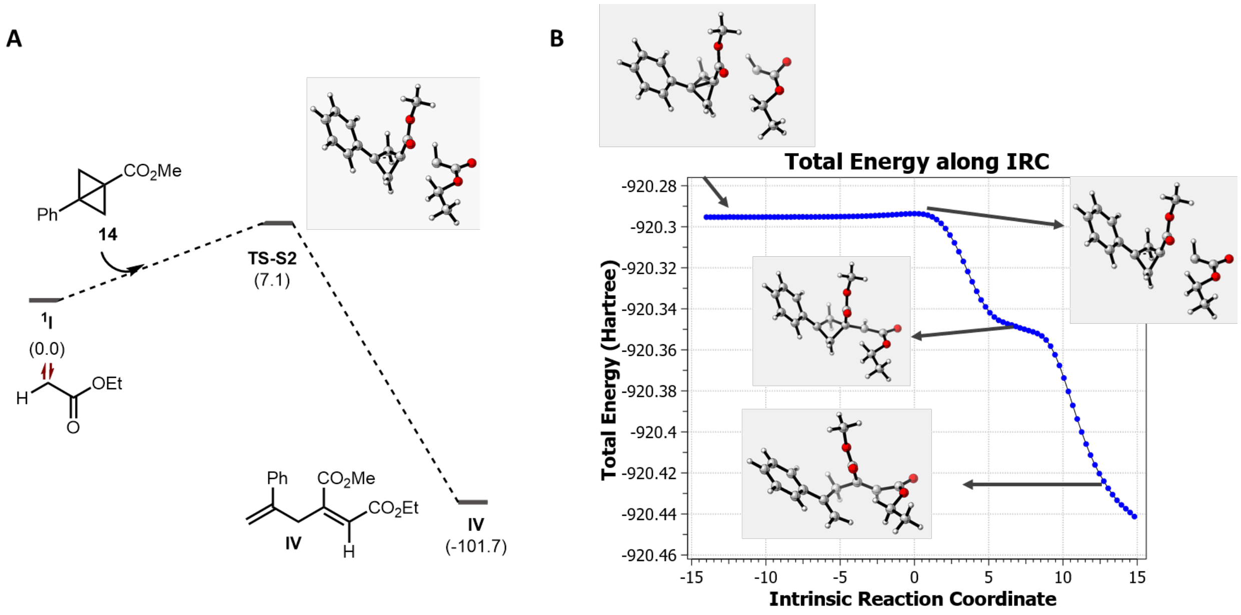

Figure S3. Reaction of **BCB** with close-shell singlet carbene. **A.** Free energy surface of the reaction (reported energies are Gibbs free energy in kcal/mol that were calculated at the {[(U)ωB97X-D] + CPCM(CH_2Cl_2)}/def2TZVPP//{[(U)ωB97X-D] + CPCM(CH_2Cl_2)}/def2SVP level of theory). **B.** IRC path initiated from **TS-S2**.

8.3. Unpaired spin-density distributions (in |e|) in the calculated important singlet diradical intermediates and transition states

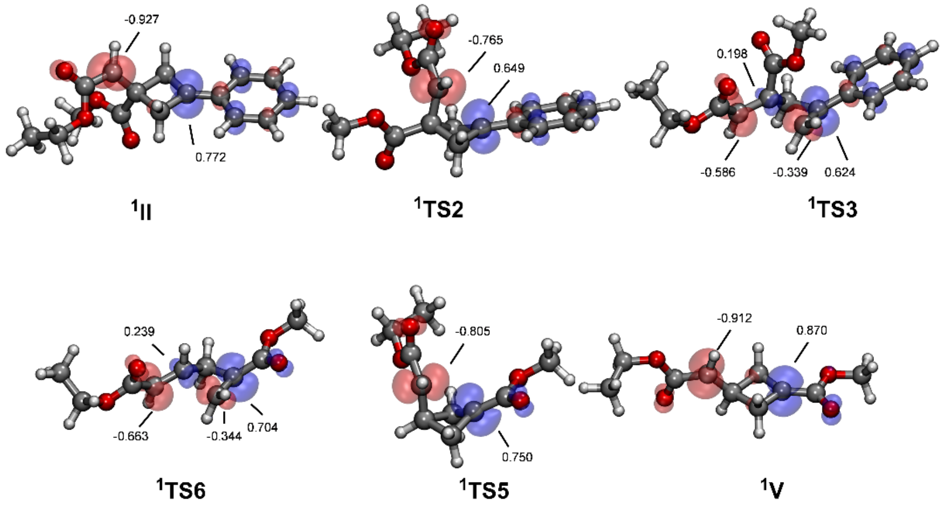

Figure S4. Unpaired spin density distributions (in |e|) in the calculated important singlet diradical intermediates and transition states

8.4. The total energies and cartesian coordinates of all calculated intermediates and transition states

Table S2. Singlet point energies (E) at def2TZVPP basic set with structure from def2SVP, energies (E), Zero-point correction (ZPE), thermal correction to enthalpy (TCH), thermal correction to Gibbs free energy (TCG), , enthalpies (H), and Gibbs free energies (G) (in Hartree) of the structures calculated at the [(U)ωB97X-D]+ CPCM(CH_2Cl_2)/def2TZVPP//[(U)ωB97X-D]+ CPCM(CH_2Cl_2)/def2SVP level of theory

Structure	E (def2TZVPP)	E	ZPE	TCH	TCG	H	G	Imaginary Frequency
**S0-[SUB-1]**	-415.977051	-415.502642	0.106125	0.111479	0.084757	-415.865572	-415.892294	-
**T1-[SUB-1]**	-415.914804	-415.442025	0.103814	0.109386	0.081388	-415.805418	-415.833416	-
**N_2**	-109.532669	-109.399606	0.005775	0.008082	-0.006266	-109.524587	-109.538935	-
**^{3}TS-[SUB-1]**	-415.89253	-415.415056	0.100538	0.106543	0.077039	-415.785987	-415.815491	544.55i
**^{3}I**	-306.382058	-306.03294	0.093172	0.097704	0.072613	-306.284354	-306.309445	-
**^{1}I**	-306.37721	-306.023574	0.093215	0.097909	0.073217	-306.279301	-306.303993	-
**14**	-614.927463	-614.261358	0.21328	0.220595	0.187456	-614.706868	-614.740007	-
**^{3}TS1a**	-921.314005	-920.303087	0.307114	0.318789	0.275115	-920.995216	-921.03889	459.87i
**^{3}TS1b**	-921.312557	-920.301205	0.306982	0.318634	0.274875	-920.993923	-921.037682	549.57i
**^{3}II**	-921.416357	-920.407899	0.309461	0.32097	0.277177	-921.095387	-921.13918	-
**^{1}II**	-921.416418	-920.407968	0.309372	0.320896	0.277829	-921.095522	-921.138589	-
**^{1}TS2**	-921.406874	-920.39675	0.310535	0.321563	0.27967	-921.085311	-921.127204	333.79i
**^{1}TS3**	-921.395655	-920.384451	0.30922	0.320378	0.278694	-921.075277	-921.116961	914.48i
**15**	-921.461474	-920.454769	0.315153	0.325928	0.284883	-921.135546	-921.176591	-
**IV**	-921.48723	-920.47331	0.312702	0.324285	0.281112	-921.162945	-921.206118	-
**36**	-383.865836	-383.439619	0.13095	0.136027	0.110084	-383.729809	-383.755752	-
**^{3}TS4a**	-690.247829	-689.476599	0.224782	0.234068	0.196885	-690.013761	-690.050944	580i
**^{3}TS4b**	-690.250454	-689.477561	0.224705	0.234255	0.194726	-690.016199	-690.055728	450.33i
**^{3}V**	-690.355569	-689.584328	0.228321	0.237591	0.19945	-690.117978	-690.156119	-

**[1]V**	-690.355612	-689.584382	0.22825	0.237525	0.200094	-690.118087	-690.155518	-
**[1]TS5**	-690.338905	-689.567828	0.229273	0.23783	0.202544	-690.101075	-690.136361	419.96i
**[1]TS6**	-690.340772	-689.567471	0.227385	0.236373	0.200191	-690.104399	-690.140581	881.43i
**VI**	-690.401658	-689.63368	0.23327	0.241717	0.206547	-690.159941	-690.195111	-
**VII**	-690.437487	-689.663286	0.230465	0.239863	0.202304	-690.197624	-690.235183	-

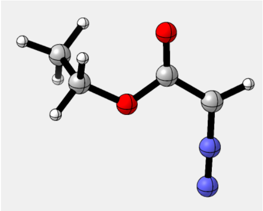

S0 [SUB-1]

C	-0.38236500	-0.00481800	-0.19358900
O	0.64428100	-0.58514000	-0.46377500
O	-1.54429400	-0.60918300	0.08564200
C	-1.56671700	-2.04076900	0.09283600
H	-2.61283100	-2.30465600	-0.10894500
H	-0.94350800	-2.41307200	-0.73249100
C	-0.49522600	1.44708200	-0.14055900
H	0.34420000	2.10939500	-0.34085800
C	-1.10515900	-2.60017500	1.42300200
H	-1.20388900	-3.69539400	1.41804600
H	-0.05084600	-2.35044100	1.60728100
H	-1.71440400	-2.20167400	2.24714700
N	-1.63858500	2.00232600	0.15771300
N	-2.62906600	2.47044000	0.41675800

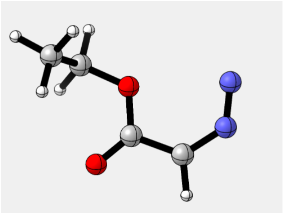

T1 [SUB-1] (Triplet State)

C	-0.40439800	0.01773300	-0.18169200
O	0.63217400	-0.54734600	-0.44663600
O	-1.55490600	-0.57471600	0.08613600
C	-1.58865800	-2.01154800	0.08994500
H	-2.63795600	-2.26140600	-0.10828700
H	-0.97121800	-2.38235500	-0.74011000
C	-0.46578900	1.49984300	-0.14280300
H	0.45869300	2.02840900	-0.37961400
C	-1.12656900	-2.57186600	1.41869400
H	-1.23639000	-3.66568800	1.41059500
H	-0.06943800	-2.33386900	1.60175900
H	-1.73133700	-2.16913800	2.24364600
N	-1.54416400	2.27567400	0.16047500
N	-2.65845200	1.92019300	0.45610100

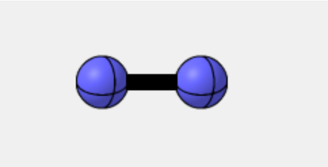

N_2

N	2.25131000	0.28930100	0.00000000
N	1.15450700	0.28930100	0.00000000

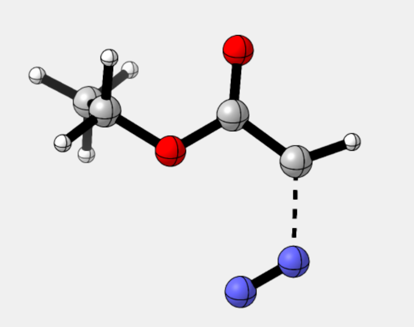

^{3}TS-[SUB-1] (Triplet state)

C	-0.30804100	-0.03772400	-0.24795600
O	0.72419200	-0.64917100	-0.43533100

O	-1.49124700	-0.59829200	-0.01637700
C	-1.55416700	-2.02940600	0.05453500
H	-2.59798200	-2.27183900	-0.18069300
H	-0.90683000	-2.45467000	-0.72550900
C	-0.35572000	1.41688300	-0.25933000
H	0.49320100	2.05928400	-0.49435000
C	-1.16627900	-2.53568600	1.42867100
H	-1.29725200	-3.62660800	1.46890300
H	-0.11403200	-2.30764200	1.64901700
H	-1.79957300	-2.08188900	2.20442300
N	-1.78417000	2.41562900	0.34241600
N	-2.74050900	1.92505300	0.67979200

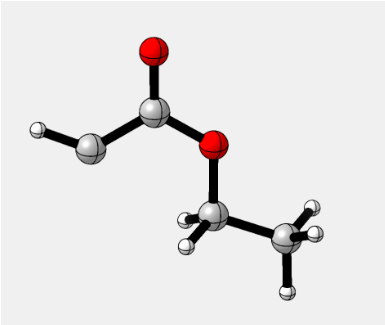

^{3}I (Triplet state)

C	0.02603300	0.69042400	-0.52976000
O	1.23381900	0.54864100	-0.53140200
O	-0.77694500	-0.37068700	-0.52989800
C	-2.19658400	-0.18557900	-0.52732700
H	-2.48790600	0.38968800	0.36654700
H	-2.49070600	0.39305400	-1.41810500
C	-2.84547300	-1.54978600	-0.52889100
H	-2.55242500	-2.12190800	0.36279400
H	-3.93863300	-1.43845400	-0.52698000
H	-2.55519900	-2.11853300	-1.42363700
C	-0.55033500	2.01584900	-0.52642000

H	-0.04336500	2.98353100	-0.52539500

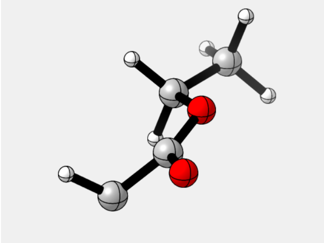

[1]I (Singlet state)

C	0.05921700	0.56272900	-0.51458200
O	1.29430800	0.53425200	-0.55461000
O	-0.73171500	-0.44365400	-0.71439400
C	-2.15294700	-0.18418400	-0.65110500
H	-2.34976900	0.48252200	0.20325100
H	-2.44493400	0.33285500	-1.57684200
C	-2.86764700	-1.50305000	-0.50069300
H	-2.56598700	-2.00804000	0.42749500
H	-3.95143700	-1.32656900	-0.46502800
H	-2.65197600	-2.16399900	-1.35178100
C	-0.05595500	1.89124600	-0.07505500
H	-0.02774500	2.58985300	-0.92997900

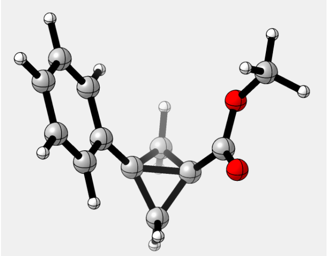

BCB 14

C	1.35617700	0.24045400	0.42987500
C	2.65687300	-0.18994500	1.02724400
C	2.51717600	1.21820000	0.53652900
C	1.19165400	1.54181600	1.14796600
H	3.28651100	-0.85604500	0.43126300
H	2.71647200	-0.34935300	2.11122800

H	0.60560200	2.31922700	0.64937900
H	1.11414200	1.53834300	2.24239200
C	0.69740000	-0.23698700	-0.79980400
C	-0.37546300	0.47186300	-1.36044700
C	1.14864300	-1.39637800	-1.44799500
C	-0.96520200	0.04433700	-2.54823800
H	-0.75969700	1.36335800	-0.86126500
C	0.55728200	-1.82164600	-2.63576800
H	1.96578100	-1.97757200	-1.01644800
C	-0.49959700	-1.10166200	-3.19319600
H	-1.79862300	0.61012700	-2.97032900
H	0.92300600	-2.72637200	-3.12632100
H	-0.96464900	-1.43759100	-4.12239000
C	3.09723800	1.85702200	-0.64952100
O	4.07641800	1.12228900	-1.18129400
O	2.70972100	2.90140100	-1.12084600
C	4.65179900	1.60556100	-2.38767300
H	3.89387900	1.67256300	-3.18089500
H	5.09872400	2.59803400	-2.23626100
H	5.42592900	0.88370400	-2.66801300

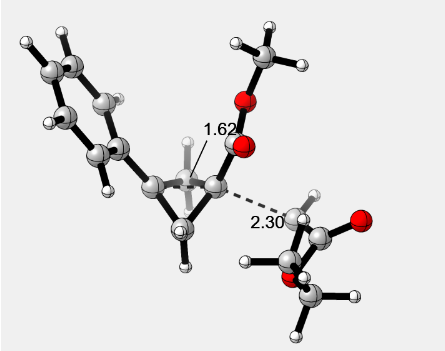

^{3}**TS1a** (Triplet state)

C	2.77255800	1.49956500	0.69436000
C	3.77061100	0.69995800	1.45651200
C	4.35862700	1.54843100	0.35882600

C	3.52926100	2.77884800	0.59864000
H	3.78086300	-0.38370800	1.31285000
H	4.01516300	1.01893900	2.47777000
H	3.34356900	3.42231700	-0.26546000
H	3.74949200	3.32480500	1.52393500
C	1.73172000	1.00306800	-0.21103000
C	1.10350500	1.86753300	-1.12354800
C	1.35590900	-0.35118300	-0.20012100
C	0.14343000	1.38694500	-2.01000000
H	1.36282800	2.92754900	-1.13588200
C	0.39608800	-0.82774900	-1.08870700
H	1.81275700	-1.03783400	0.51469300
C	-0.21157600	0.03745000	-1.99985000
H	-0.33343500	2.07354800	-2.71268900
H	0.11726500	-1.88340700	-1.06639300
H	-0.96588200	-0.33761900	-2.69465100
C	4.68249600	1.08097500	-1.00209400
O	4.92679600	-0.22799600	-1.03540200
O	4.71393800	1.79816500	-1.97585300
C	5.21605200	-0.78898900	-2.30992900
H	4.36723300	-0.65304200	-2.99448300
H	6.10823600	-0.31857200	-2.74581900
H	5.39610500	-1.85544700	-2.14039700
C	6.54778900	2.01821300	0.87793100
C	7.01454800	3.08645900	0.02855100
O	7.93946000	2.95717000	-0.75358700
O	6.30275300	4.21174500	0.16278200
C	6.59264200	5.29332800	-0.72769800
H	6.29457000	6.19807900	-0.18183100
H	7.67611100	5.33229200	-0.90801400

C	5.82675100	5.15720600	-2.02822600
H	4.74279400	5.14490900	-1.84504000
H	6.05792200	6.00841100	-2.68523400
H	6.10015100	4.22809100	-2.54644000
H	7.09234000	1.09482900	1.09007900

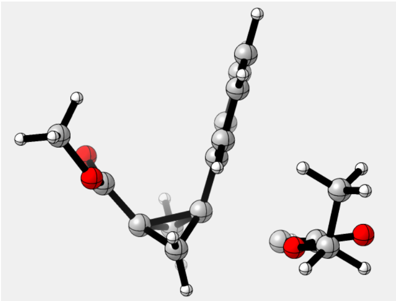

[3]**TS1b** (Triplet state)

C	2.75999600	1.50923900	1.06375800
C	3.84251400	0.62296700	1.56279100
C	4.27261300	1.56848000	0.46489600
C	3.51859800	2.78335800	0.94770800
H	3.75710700	-0.43983200	1.31785600
H	4.24497600	0.82424700	2.56314900
H	3.17638200	3.50677800	0.20441100
H	3.88671200	3.23839500	1.87525100
C	1.68743000	1.04314200	0.18028100
C	6.43411700	2.00633300	0.91883500
C	7.06685700	3.08437100	0.19473800
O	8.24904200	3.08719400	-0.09754000
O	6.22157800	4.07178900	-0.12521000
C	6.74320400	5.17722700	-0.86907900
H	6.05301900	6.00468500	-0.65986500
H	7.73575200	5.43861700	-0.47459500
C	6.81108700	4.88297600	-2.35441600
H	5.81847000	4.63235400	-2.75414800
H	7.18409100	5.76953000	-2.88769800

H	7.49464300	4.04667700	-2.55687800
H	6.96599800	1.14713600	1.33541600
O	1.13711600	2.04572300	-0.50955300
O	1.35906200	-0.11618300	0.05864200
C	0.15131600	1.69116900	-1.46909900
H	-0.18455300	2.62887200	-1.92389300
H	0.57878900	1.03154100	-2.23765000
H	-0.69480400	1.17981600	-0.98932400
C	4.48686400	1.18765400	-0.95322500
C	4.30986900	2.12181600	-1.98323400
C	4.87690800	-0.11703200	-1.28585800
C	4.49774300	1.75307600	-3.31307300
H	4.03552900	3.14935200	-1.74256200
C	5.06526100	-0.48317900	-2.61708200
H	5.04645200	-0.85190600	-0.49648500
C	4.87346700	0.44894500	-3.63670600
H	4.35473000	2.49405500	-4.10273200
H	5.36890500	-1.50427900	-2.85762100
H	5.02406100	0.16253800	-4.67964200

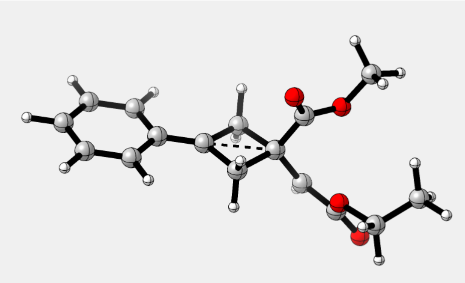

³II (triplet state)

C	-1.62462300	-0.67876300	0.40640900
C	-0.38462600	-1.47310000	0.68967600
C	0.46024200	-0.22488700	0.25259400
C	-0.87675400	0.57350300	0.05810200
H	-0.23568800	-2.38208300	0.08257500

H	-0.23371900	-1.74876600	1.74751300
H	-1.01602800	0.95746400	-0.96473600
H	-0.99905400	1.40910500	0.76784700
C	-2.99633300	-1.00280400	0.42833600
C	-3.98248000	-0.03618100	0.08888200
C	-3.43842300	-2.30520500	0.78945700
C	-5.33123400	-0.36057300	0.11079700
H	-3.66750300	0.97172900	-0.19250800
C	-4.79051200	-2.61596000	0.80724800
H	-2.70018300	-3.06594600	1.05511100
C	-5.74658200	-1.64961600	0.46900200
H	-6.07375200	0.39594300	-0.15366300
H	-5.11035700	-3.62238000	1.08734100
H	-6.80940500	-1.89968300	0.48453200
C	1.15517600	-0.49601900	-1.07697700
O	2.41048500	-0.89781400	-0.89510400
O	0.62861500	-0.42178800	-2.15744000
C	3.15770000	-1.22139600	-2.06127900
H	2.70960300	-2.07879900	-2.58254900
H	3.19352400	-0.36390600	-2.74737700
H	4.16623400	-1.47330400	-1.71800000
C	1.38535400	0.32493200	1.27519400
C	2.18671000	1.51262400	1.03334700
O	2.99520000	1.96859900	1.81761700
O	1.93287800	2.04959600	-0.17183600
C	2.72722600	3.15909300	-0.59664600
H	2.10696800	3.68587900	-1.33300600
H	2.90152200	3.82454900	0.26060500
C	4.03478500	2.69660900	-1.20688300
H	3.85252800	2.02179900	-2.05558100

H	4.60295900	3.56444400	-1.57174100
H	4.64943300	2.17178300	-0.46187400
H	1.48767800	-0.14085700	2.25696500

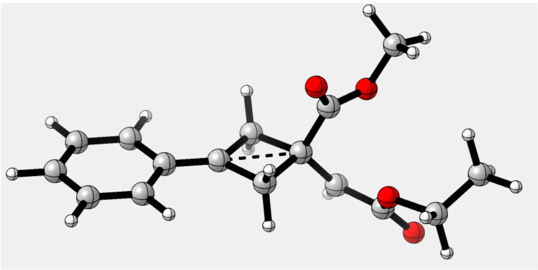

[1]II

C	-1.62792000	-0.68781800	0.37505800
C	-0.38799900	-1.48388000	0.65445900
C	0.45738900	-0.23183700	0.22953900
C	-0.87967100	0.56428600	0.02912900
H	-0.23655900	-2.38821400	0.04103100
H	-0.24057000	-1.76744100	1.71074200
H	-1.01129200	0.94538200	-0.99573600
H	-1.00791100	1.40186600	0.73599200
C	-3.00086700	-1.00467100	0.42009100
C	-3.98745000	-0.03339700	0.09567700
C	-3.44352800	-2.30450100	0.78948000
C	-5.33729200	-0.35096000	0.13974400
H	-3.67194300	0.97257900	-0.19198000
C	-4.79669300	-2.60856300	0.82908000
H	-2.70486700	-3.06865400	1.04395600
C	-5.75323300	-1.63766900	0.50556200
H	-6.08020000	0.40908600	-0.11325400
H	-5.11701300	-3.61316100	1.11510600
H	-6.81690200	-1.88242400	0.53831100
C	1.16480700	-0.49327500	-1.09554100
O	2.41572400	-0.90407100	-0.90370700

O	0.65095000	-0.40503400	-2.18101400
C	3.17442500	-1.21938200	-2.06474400
H	2.72727800	-2.06822400	-2.60061400
H	3.22332400	-0.35463200	-2.74084000
H	4.17739300	-1.48123700	-1.71271200
C	1.37314100	0.31493000	1.26230000
C	2.17456300	1.50499300	1.03258500
O	2.97356800	1.96041400	1.82683200
O	1.93242200	2.04516800	-0.17359200
C	2.72758900	3.15889100	-0.58565400
H	2.11413700	3.68541800	-1.32787100
H	2.88894500	3.82249100	0.27556100
C	4.04423400	2.70340400	-1.18146100
H	3.87494900	2.03047800	-2.03433300
H	4.61327100	3.57460200	-1.53683700
H	4.65196600	2.17880700	-0.43064400
H	1.46939500	-0.15592800	2.24224600

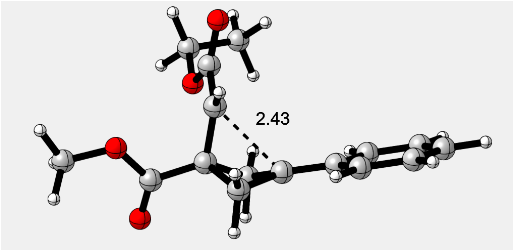

[1]TS2

C	2.12559900	0.89559000	-0.51028600
C	3.33257800	0.06138500	-0.86506500
C	4.01758700	1.45141300	-0.90733000
C	2.64257000	2.04518000	-1.33371200
H	3.23214100	-0.40188700	-1.86274900
H	3.73525500	-0.66679300	-0.15140100
H	2.43222700	1.90508800	-2.40833500
H	2.43169100	3.08285600	-1.03854600

C	0.87584300	0.60737200	0.11120100
C	-0.15003100	1.57981300	0.16702900
C	0.64361400	-0.65591300	0.70429500
C	-1.35783300	1.29485200	0.79071300
H	0.01411900	2.55999600	-0.28622700
C	-0.56781400	-0.93132200	1.32500400
H	1.42830000	-1.41510300	0.67155800
C	-1.57273800	0.04078500	1.37239000
H	-2.14254100	2.05361700	0.82562800
H	-0.73499600	-1.91101000	1.77772000
H	-2.52356500	-0.17899900	1.86236800
C	5.23295400	1.65406000	-1.78180500
O	6.03601900	2.60167300	-1.30683800
O	5.44152700	1.04677700	-2.80285300
C	7.20855300	2.88947900	-2.05873400
H	7.84795000	1.99879000	-2.13233700
H	6.94681700	3.22670300	-3.07136200
H	7.73146900	3.68548700	-1.51883500
C	4.08547200	1.87867000	0.53183100
C	4.89917300	1.22025200	1.54134900
O	4.93172600	1.54487200	2.71304500
O	5.62599600	0.20634500	1.03859000
C	6.41546200	-0.56474500	1.94583100
H	7.20697600	-1.00645900	1.32666000
H	6.87652400	0.10815800	2.68277800
C	5.58904900	-1.63645900	2.62865500
H	5.11790800	-2.29898500	1.88776700
H	6.23361700	-2.24731200	3.27735000
H	4.80388400	-1.18483100	3.25122300
H	3.67308300	2.84286200	0.83515600

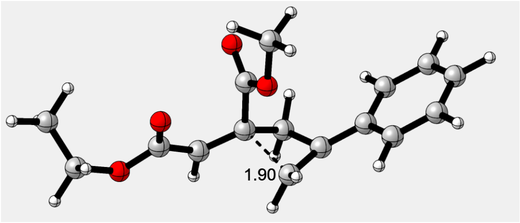

[1]TS3

C	2.41670200	0.39251600	0.55080700
C	3.78912400	-0.02774200	0.49400900
C	3.99192200	1.78058000	1.02919400
C	2.56305400	1.52955600	1.53285400
H	4.21272200	-0.46555200	1.40092500
H	4.23135700	-0.41592400	-0.43054300
H	1.88768100	2.38656500	1.40727600
H	2.54704000	1.22134400	2.58998400
C	1.43276500	0.27009800	-0.48510800
C	0.25289600	1.05156100	-0.47160200
C	1.60741400	-0.64753200	-1.55006000
C	-0.69781900	0.92504100	-1.47735700
H	0.08377000	1.76424700	0.33768900
C	0.65857300	-0.75906400	-2.55701000
H	2.49522000	-1.28239800	-1.57582600
C	-0.49983700	0.02489200	-2.52781600
H	-1.60133100	1.53780400	-1.44631900
H	0.81584300	-1.46984300	-3.37122200
H	-1.24697300	-0.06983000	-3.31846400
C	3.99313000	2.65490600	-0.21731600
O	4.20132800	1.99929500	-1.34963100
O	3.74694100	3.83394200	-0.15919100
C	4.18626500	2.77233100	-2.54203600
H	3.22413000	3.29043100	-2.65669000
H	4.99691600	3.51411400	-2.52584800

H	4.33631100	2.06626600	-3.36513200
C	5.07667300	1.94765200	1.92484200
C	6.39438100	2.33572700	1.45958100
O	6.67611300	2.61761600	0.30709800
O	7.29020900	2.36802200	2.45948400
C	8.61457000	2.80428300	2.15017400
H	9.24526500	2.37174700	2.93801000
H	8.91936700	2.38006200	1.18269900
C	8.71884800	4.31689000	2.13594500
H	8.38780200	4.73986700	3.09560500
H	9.76351500	4.61667100	1.96766800
H	8.10439200	4.74191500	1.33000200
H	4.97018800	1.62945400	2.96357500

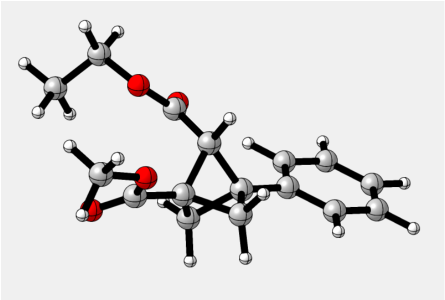

BCP 15

C	-1.20605800	0.10075100	-0.37677500
C	-0.47314600	-0.95232900	-1.27169400
C	0.46467800	0.27114800	-1.22417600
C	-0.78127100	1.16199400	-1.43285600
H	-0.94192000	-1.16240000	-2.24374500
H	-0.13452200	-1.87509100	-0.78226900
H	-1.26320800	1.08640400	-2.41802600
H	-0.70787500	2.20348700	-1.08849600
C	-2.55161700	-0.01551000	0.25669500
C	-3.59373900	0.82452000	-0.15089000
C	-2.79869000	-0.97075900	1.25201100

C	-4.86170900	0.71317600	0.42115700
H	-3.41273100	1.57455100	-0.92479700
C	-4.06457000	-1.08075800	1.82394800
H	-1.98090100	-1.61562000	1.57973100
C	-5.10033000	-0.24058300	1.40978300
H	-5.66559100	1.37573600	0.09252500
H	-4.24500500	-1.82790700	2.60031100
H	-6.09204500	-0.32904200	1.85909400
C	1.82680000	0.38457500	-1.82925800
O	2.28990100	1.62876900	-1.75522600
O	2.44369600	-0.53983100	-2.29883400
C	3.60758200	1.85377600	-2.24227600
H	4.33607500	1.26129200	-1.67107900
H	3.68064200	1.58311700	-3.30466900
H	3.80501700	2.92247900	-2.11037900
C	0.17052200	0.37968600	0.30226200
C	0.85884600	-0.62959500	1.18615200
O	0.34861900	-1.56108400	1.76031400
O	2.16037700	-0.34921500	1.23779200
C	3.02450500	-1.24925300	1.94485100
H	3.89806200	-0.64291900	2.21517700
H	2.52265100	-1.57951800	2.86497900
C	3.41398800	-2.42468500	1.07222300
H	3.86337900	-2.07645200	0.13129400
H	4.14690300	-3.05229500	1.59923500
H	2.53718000	-3.04401800	0.83598300
H	0.30571500	1.38882900	0.72228000

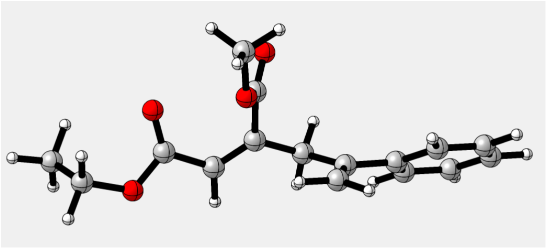

IV

C	2.55055100	0.45315000	-0.36483400
C	3.53634300	-0.35184200	-0.78338100
C	4.23022200	2.21633100	0.38601300
C	2.82726400	1.68668300	0.47865300
H	4.58487500	-0.12927900	-0.57389000
H	3.32738200	-1.26528800	-1.34462000
H	2.15261000	2.49813400	0.15709400
H	2.58654700	1.47913200	1.53310800
C	1.12021600	0.16348500	-0.66735400
C	0.10430900	0.51844400	0.23386200
C	0.74975200	-0.46795600	-1.86550200
C	-1.23166900	0.22965500	-0.04154900
H	0.35267000	1.01532500	1.17383700
C	-0.58471400	-0.75453000	-2.14271300
H	1.51659800	-0.71988000	-2.60120300
C	-1.58220900	-0.40949300	-1.22993800
H	-2.00336100	0.50745100	0.67978000
H	-0.84814400	-1.24152200	-3.08427800
H	-2.62913900	-0.63085200	-1.44826000
C	4.59976000	2.78385200	-0.96019600
O	5.58510900	2.11753400	-1.54274900
O	4.00723900	3.70713300	-1.45994100
C	6.04401900	2.61940800	-2.79208900
H	5.22293100	2.66221700	-3.52062800
H	6.46770400	3.62495800	-2.66153600

H	6.81856500	1.92564200	-3.13399900
C	5.06203100	2.26623900	1.43464600
C	6.40219400	2.90335000	1.38668000
O	6.77204900	3.68279100	0.53867800
O	7.14698500	2.51792200	2.42060800
C	8.46287000	3.07322600	2.54445000
H	9.01853200	2.34048900	3.14309600
H	8.91997400	3.13576200	1.54691800
C	8.43374800	4.42876900	3.22067000
H	7.94934700	4.36369600	4.20559800
H	9.46196900	4.79095000	3.36426100
H	7.89363200	5.16218800	2.60586500
H	4.75770200	1.84423400	2.39509300

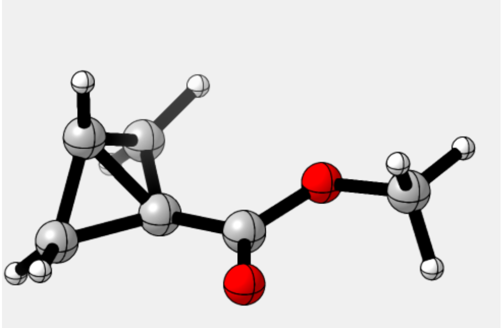

BCB 35

C	1.40108600	0.16748900	0.44124100
C	2.75363500	-0.24142200	0.91413900
C	2.51238700	1.17479100	0.47920100
C	1.23248000	1.42726000	1.21814300
H	3.34554300	-0.86820600	0.24131100
H	2.91301400	-0.43074900	1.98410300
H	0.56731400	2.18522100	0.79400600
H	1.25339500	1.38871700	2.31537100
C	3.03565400	1.92691800	-0.66770100
O	4.05857300	1.29819000	-1.25269300

O	2.59536800	2.98567900	-1.05242600
C	4.61546000	1.92136800	-2.40170200
H	3.86672400	2.00751500	-3.20199000
H	4.99142900	2.92485200	-2.15759800
H	5.44084100	1.28001700	-2.72817400
H	0.86064500	-0.16335400	-0.44385000

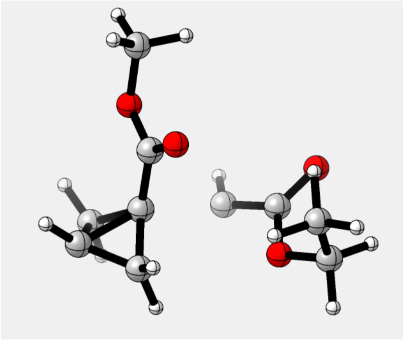

^{3}TS4a (Triplet State)

C	2.76256200	1.45236200	0.73548500
C	3.78993800	0.64196000	1.42867500
C	4.33621500	1.53463500	0.33556400
C	3.47710200	2.74397400	0.61646300
H	3.79456300	-0.43683700	1.25030600
H	4.07979100	0.93212300	2.44806900
H	3.23092300	3.38599700	-0.23365200
H	3.73248200	3.28932700	1.53442500
C	4.63554000	1.09901200	-1.04904300
O	4.96915600	-0.18940700	-1.10424100
O	4.61543700	1.83060900	-2.01141300
C	5.31367300	-0.70436800	-2.38434400
H	4.46879500	-0.61467900	-3.08129800
H	6.17756000	-0.16502800	-2.79717400
H	5.56535700	-1.75886100	-2.23166400
C	6.40504700	2.00352500	0.81675900
C	6.94133100	3.09884400	0.04372000

O	7.95277600	3.01372000	-0.63102500
O	6.18822300	4.20453900	0.11246200
C	6.55556800	5.31273500	-0.71265100
H	6.11944700	6.18958500	-0.21639300
H	7.65011700	5.41719900	-0.71271600
C	6.02281900	5.15913600	-2.12324300
H	4.93033300	5.03822300	-2.11768400
H	6.27315700	6.05283700	-2.71339200
H	6.46693400	4.28208600	-2.61337800
H	6.98042200	1.11062500	1.07605300
H	2.01878100	1.06957800	0.03590800

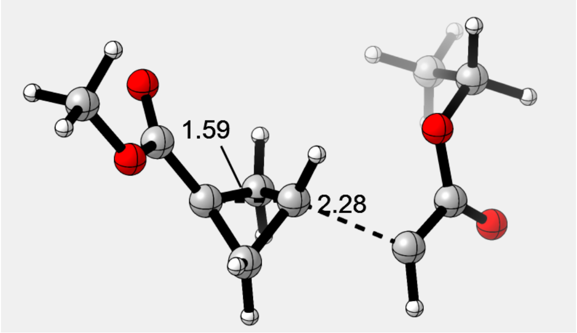

[3]**TS4b** (Triplet state)

C	3.12648200	1.24753400	0.41660600
C	4.53419300	1.40196900	-0.04216000
C	3.95199700	2.57642400	0.68965900
C	3.23540600	1.83672400	1.78128900
H	4.69443500	1.51030400	-1.11841800
H	5.30920200	0.82571500	0.48073100
H	2.33670600	2.29752800	2.20001000
H	3.86091900	1.30887100	2.51344500
C	1.96001300	1.22749300	-0.47291900
C	5.73475600	3.66750400	1.60619700
C	6.71295900	4.06564400	0.62214000
O	7.91233100	4.07521000	0.83322900

O	6.16126000	4.40033400	-0.55120300
C	7.03921800	4.73188500	-1.63089500
H	6.43628900	5.35809700	-2.30105300
H	7.87624100	5.33175800	-1.24574300
C	7.53873900	3.48995200	-2.34184100
H	6.69705100	2.88422400	-2.70783000
H	8.15739800	3.77863300	-3.20389900
H	8.15274300	2.87515100	-1.66871000
H	5.94675100	3.44653400	2.65516100
O	0.81999600	1.44313900	0.18984800
O	2.01362600	1.03828100	-1.66761600
C	-0.37057700	1.47209800	-0.58453000
H	-1.19070300	1.64257900	0.12077800
H	-0.33590500	2.28409600	-1.32478800
H	-0.51740100	0.51804100	-1.11003600
H	3.59291800	3.48416200	0.20677800

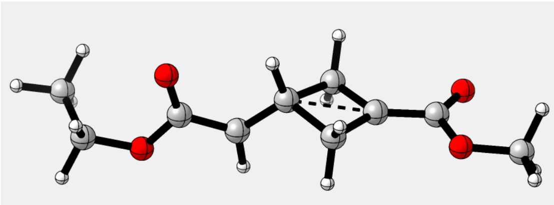

^{3}**V** (triplet state)

C	4.42322600	0.45032900	-0.13118800
C	5.52349700	-0.02372800	-1.14498500
C	4.92374100	0.89795300	-2.15117900
C	3.94662500	1.50113300	-1.19884300
H	5.51199300	-1.09700900	-1.39456600
H	6.55383100	0.24253000	-0.85005700
H	2.88352100	1.44346200	-1.48264400
H	4.16839000	2.54932100	-0.93185600
C	5.19886500	1.07395800	-3.55540300

O	4.40862900	2.00367300	-4.11620700
O	6.04767000	0.45658700	-4.17272100
C	4.60270200	2.24975500	-5.49947700
H	5.62718700	2.59735900	-5.69696500
H	4.41942300	1.34025800	-6.08995200
H	3.88371000	3.02756300	-5.77836300
C	4.88673500	0.97980800	1.16275100
C	4.30737200	0.53924900	2.42162000
O	3.41906200	-0.28736300	2.52134800
O	4.86537700	1.15610400	3.47334700
C	4.39953800	0.80000000	4.77713300
H	4.64012400	1.66584100	5.40764400
H	3.30728100	0.67667100	4.74859500
C	5.07489700	-0.45591500	5.29178300
H	6.16834700	-0.33902600	5.29188900
H	4.74848800	-0.65637000	6.32261300
H	4.81022200	-1.32417700	4.67224900
H	5.67857200	1.73336500	1.19573300
H	3.66332700	-0.32355400	0.03654700

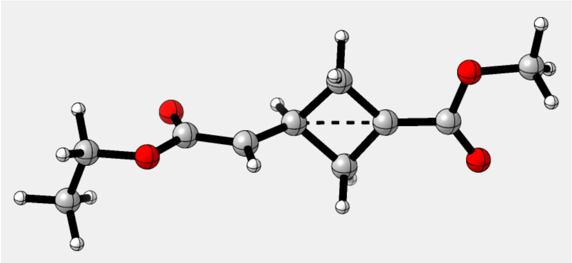

¹V

C	4.41571800	0.44110100	-0.13198900
C	5.51979800	-0.02791200	-1.14448000
C	4.91892000	0.89201400	-2.15141700
C	3.93171800	1.48499200	-1.20334300
H	5.51085900	-1.10109600	-1.39470300

H	6.54915500	0.24011400	-0.84760500
H	2.87048800	1.41275000	-1.49090700
H	4.13845800	2.53656400	-0.93733800
C	5.20411800	1.07756000	-3.55249400
O	4.41129000	2.00410000	-4.11481300
O	6.06250500	0.46987800	-4.16609700
C	4.61442500	2.25882100	-5.49523800
H	5.63747300	2.61611200	-5.68264800
H	4.44372900	1.35078200	-6.09168900
H	3.89110000	3.03189300	-5.77606800
C	4.87607300	0.97861500	1.15966100
C	4.30335000	0.53537900	2.42065100
O	3.42327900	-0.29954600	2.52403600
O	4.85732000	1.16006800	3.46989100
C	4.39742500	0.80244200	4.77537200
H	4.63091400	1.67195200	5.40349800
H	3.30635000	0.66857200	4.74923400
C	5.08576900	-0.44580500	5.29147300
H	6.17804500	-0.31843400	5.28928500
H	4.76320800	-0.64714400	6.32334300
H	4.82826900	-1.31789800	4.67429900
H	5.66079100	1.73970500	1.18956700
H	3.66180800	-0.33780300	0.03936100

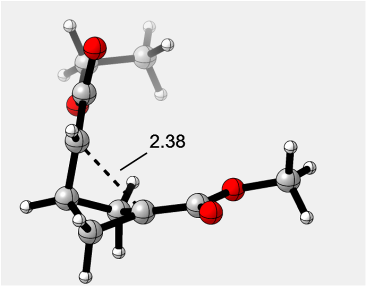

[1]TS5

C	2.09892600	1.01196400	-0.67722400

C	3.25005900	0.09157800	-0.98388900
C	3.99143100	1.45088000	-1.12746000
C	2.64379700	2.07423000	-1.58577000
H	3.10479100	-0.43339700	-1.94501200
H	3.60542700	-0.61113400	-0.22250300
H	2.40699300	1.86526700	-2.64324400
H	2.45478900	3.13250400	-1.35846500
C	4.05367300	1.95200400	0.29121800
C	4.76681500	1.31210100	1.39697800
O	4.73816800	1.72606800	2.53896500
O	5.45618200	0.22674500	1.02154700
C	6.13120800	-0.51979200	2.03821600
H	6.92941900	-1.05654900	1.50970000
H	6.58728200	0.17924500	2.75350100
C	5.19057700	-1.48073300	2.73743300
H	4.72692400	-2.16964400	2.01632800
H	5.74891400	-2.07645800	3.47407100
H	4.39762700	-0.93437900	3.26718600
H	3.70637100	2.96207700	0.52429700
C	0.82760900	0.87912000	0.01905000
O	-0.03239100	1.73524100	0.04013800
O	0.73046400	-0.28960400	0.66237000
C	-0.45530000	-0.50516000	1.41323900
H	-0.35801900	-1.49897500	1.86262500
H	-0.56223200	0.25530900	2.20012500
H	-1.34160200	-0.47015100	0.76371600
H	4.90696500	1.54064600	-1.73393500

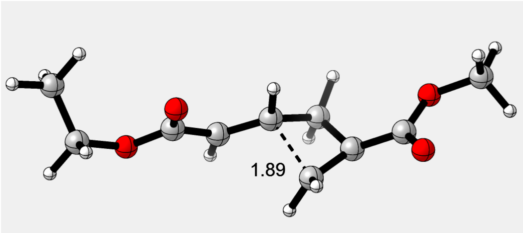

[1]TS6

C	2.42794700	0.43144600	0.59100400
C	3.80118700	0.02670900	0.50437600
C	3.97891500	1.81090700	1.11185000
C	2.53518900	1.57845500	1.55359200
H	4.24270200	-0.45965700	1.37835700
H	4.21007000	-0.32311500	-0.45196600
H	1.84735000	2.41160800	1.35506100
H	2.45545700	1.29743900	2.61611500
C	5.06550300	1.95387500	2.00171500
C	6.38278900	2.33259400	1.50156000
O	6.64245600	2.58122900	0.33999700
O	7.29096900	2.38324200	2.48761900
C	8.61924600	2.78688900	2.14880000
H	9.25315000	2.36001700	2.93703400
H	8.89993400	2.33451000	1.18680700
C	8.75211800	4.29627700	2.09833100
H	8.44272100	4.74745000	3.05233000
H	9.80015100	4.57099200	1.90971300
H	8.13589500	4.71548700	1.29067600
H	4.97574600	1.66290900	3.05096100
C	1.49358800	0.30473900	-0.51834200
O	0.37937700	1.02464500	-0.32930800
O	1.68219700	-0.38751300	-1.49952000
C	-0.61143900	0.94038500	-1.34168000
H	-0.22210300	1.30814800	-2.30192400

H	-0.95094300	-0.09723000	-1.47196300
H	-1.44463400	1.56906400	-1.01013500
H	4.08000500	2.35182400	0.16441400

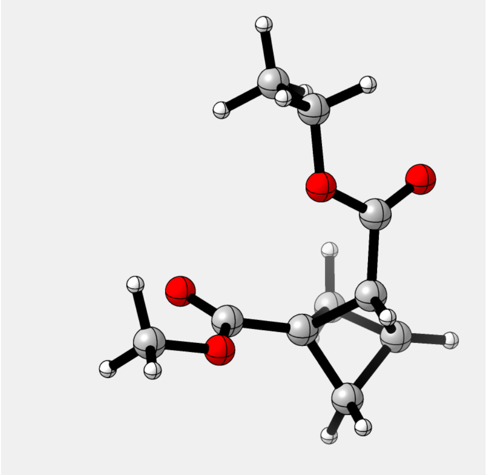

VI

C	-1.13491700	0.03898600	-0.37869900
C	-0.34966200	-0.98810600	-1.24374200
C	0.51161700	0.29354700	-1.24009100
C	-0.79788400	1.09454300	-1.46823100
H	-0.79991700	-1.26255800	-2.20851500
H	0.05854800	-1.87185700	-0.73287200
H	-1.27657300	0.95850600	-2.44828600
H	-0.78810400	2.14919900	-1.15830500
C	1.85355400	0.47318000	-1.87434900
O	2.29479000	1.72028800	-1.73249200
O	2.47119100	-0.40191000	-2.42944800
C	3.58635000	2.00742500	-2.25468000
H	4.34878400	1.38834600	-1.76129500
H	3.61905800	1.81935200	-3.33686900
H	3.77163700	3.06694200	-2.05037300
C	0.21112100	0.42712300	0.28107800
C	0.89461100	-0.53488400	1.21735400
O	0.33874100	-1.30799000	1.95752700

O	2.21847900	-0.40432000	1.12368100
C	3.04646500	-1.29210300	1.88546100
H	3.99307800	-0.75244400	2.01518200
H	2.58979600	-1.45170300	2.87217000
C	3.25154800	-2.60114100	1.15089400
H	3.65386300	-2.41991900	0.14373600
H	3.96286300	-3.23200700	1.70305000
H	2.30384400	-3.15056600	1.05899300
H	0.28771900	1.45120900	0.68126500
H	-2.09465700	-0.11215500	0.12823700

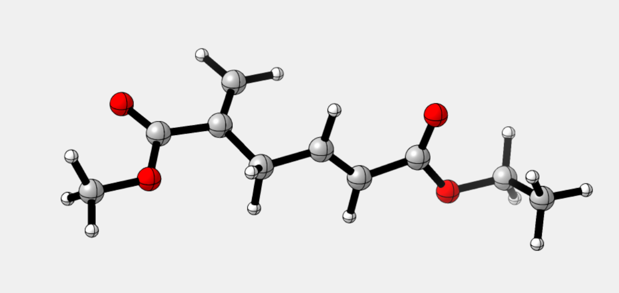

VII

C	-1.56000800	-1.29076900	-0.83545600
C	-0.70813200	-2.30843800	-0.99816900
C	0.30014100	0.31724200	-0.28595300
C	-1.17448900	0.08509400	-0.33920600
H	0.35729700	-2.20871200	-0.77983000
H	-1.07258200	-3.27399100	-1.35447600
H	-1.62585800	0.83320400	-1.01194800
H	-1.63088000	0.25634300	0.64905200
C	0.99351400	0.59121700	0.82331600
C	2.45788500	0.81888700	0.76749500
O	3.13327900	0.77601200	-0.23572200
O	2.94956100	1.07487600	1.98191600
C	4.35335300	1.33091000	2.09691700
H	4.60080900	1.07499800	3.13520200

H	4.89845800	0.65229600	1.42558700
C	4.68326600	2.77896300	1.79522600
H	4.11328100	3.45401700	2.44989600
H	5.75514200	2.95723500	1.96395000
H	4.45465200	3.02214200	0.74817800
H	0.51488800	0.65823400	1.80377500
C	-3.00497200	-1.54702500	-1.14442800
O	-3.75892300	-0.47025600	-0.92417600
O	-3.45322500	-2.59615600	-1.54127600
C	-5.15161100	-0.59674600	-1.17853000
H	-5.33175800	-0.84806200	-2.23308800
H	-5.59175100	-1.37930900	-0.54483400
H	-5.59579300	0.37537900	-0.94112100
H	0.85080600	0.25964200	-1.23262400

9. X-ray determination

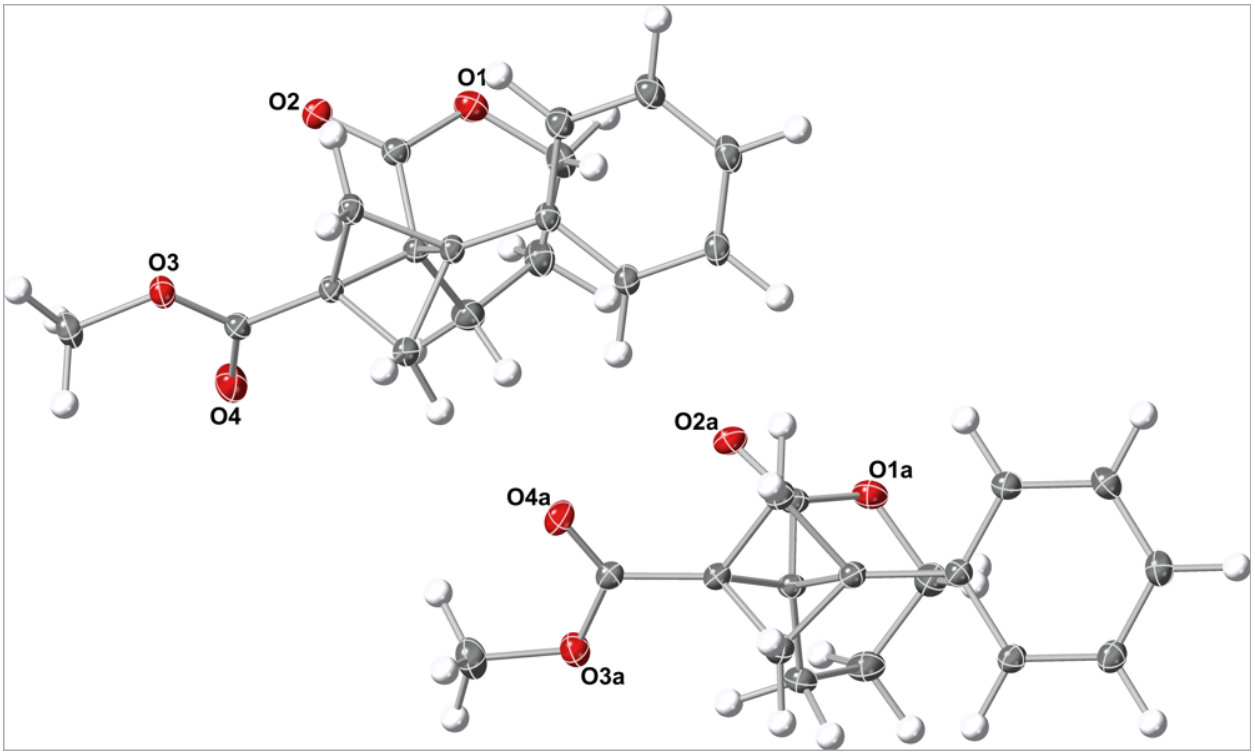

Table 1 Crystal data and structure refinement for DL-08-62-08.

Identification code	DL-08-62-08
Empirical formula	$C_{17}H_{18}O_4$
Formula weight	286.330
Temperature/K	100.01(10)
Crystal system	monoclinic
Space group	$P2_1$
a/Å	14.11139(11)
b/Å	7.20455(5)
c/Å	14.46648(10)
α/°	90
β/°	99.1803(7)
γ/°	90
Volume/Å^3	1451.913(19)

Z	4
ρ_{calc}g/cm^3	1.310
μ/mm^{-1}	0.761
F(000)	610.1
Crystal size/mm^3	0.293 × 0.225 × 0.165
Radiation	Cu Kα (λ = 1.54184)
2Θ range for data collection/°	6.18 to 154.18
Index ranges	-17 ≤ h ≤ 14, -9 ≤ k ≤ 9, -18 ≤ l ≤ 17
Reflections collected	23859
Independent reflections	5609 [R_{int} = 0.0184, R_{sigma} = 0.0145]
Data/restraints/parameters	5609/1/679
Goodness-of-fit on F^2	1.139
Final R indexes [I>=2σ (I)]	R_1 = 0.0112, wR_2 = 0.0235
Final R indexes [all data]	R_1 = 0.0117, wR_2 = 0.0236
Largest diff. peak/hole / e Å^{-3}	0.34/-0.18
Flack parameter	-0.02(3)

Table 2 Fractional Atomic Coordinates (×10^4) and Equivalent Isotropic Displacement Parameters (Å^2×10^3) for DL-08-62-08. U_{eq} is defined as 1/3 of the trace of the orthogonalised U_{IJ} tensor.

Atom	*x*	*y*	*z*	U(eq)
O1	2355.6(3)	8608.8(6)	8290.2(3)	24.38(9)
O2	1002.4(3)	7995.7(6)	7364.4(3)	23.86(9)
O3	265.3(3)	4734.3(6)	5423.8(3)	19.16(9)
O4	1517.9(3)	6136.0(6)	4947.1(3)	28.19(10)
C1	3385.8(4)	8321.4(10)	8455.9(4)	22.15(12)
C2	3756.7(5)	8460.6(9)	7531.9(4)	22.61(12)
C3	3281.6(4)	6995.5(9)	6833.2(4)	21.38(12)

Table 2 Fractional Atomic Coordinates (×10[4]) and Equivalent Isotropic Displacement Parameters (Å[2]×10[3]) for DL-08-62-08. U_{eq} is defined as 1/3 of the trace of the orthogonalised U_{IJ} tensor.

Atom	*x*	*y*	*z*	U(eq)
C4	2378.2(4)	6147.0(8)	7138.2(3)	15.24(11)
C5	1712.3(4)	4819.3(8)	6476.0(3)	15.40(11)
C6	1346.4(4)	3962.2(9)	7339.4(4)	17.91(12)
C7	2454.8(4)	4130.5(8)	7625.1(3)	15.81(11)
C8	2527.3(4)	3337.7(9)	6634.5(4)	17.40(11)
C9	1844.4(4)	7632.1(8)	7596.2(3)	18.27(11)
C10	1169.9(4)	5325.5(7)	5540.5(4)	16.45(11)
C11	-294.0(5)	5040.8(10)	4511.9(4)	23.09(13)
C12	3042.0(4)	3613.5(8)	8532.5(3)	16.10(11)
C13	4014.1(4)	3170.8(8)	8584.5(4)	18.46(11)
C14	4563.7(4)	2729.6(9)	9446.0(4)	22.19(12)
C15	4147.4(4)	2731.5(9)	10257.3(4)	24.00(13)
C16	3183.2(4)	3192.2(9)	10208.6(4)	25.56(13)
C17	2631.9(4)	3621.5(9)	9348.6(4)	21.49(12)
O1a	7749.8(3)	7234.7(5)	7856.8(3)	21.39(9)
O2a	6194.5(3)	6742.7(6)	7484.2(3)	23.04(9)
O3a	5644.1(3)	3648.2(6)	5278.9(3)	23.05(9)
O4a	4700.0(3)	2883.7(7)	6323.0(3)	32.60(11)
C1a	8680.1(4)	6764.1(10)	7618.6(4)	21.87(12)
C2a	8562.1(5)	6463.2(9)	6572.3(4)	23.60(13)
C3a	7885.6(4)	4818.1(9)	6280.5(4)	19.10(12)
C4a	7244.4(4)	4386.6(7)	7021.0(4)	15.22(11)
C5a	6390.5(4)	3000.6(8)	6817.2(3)	16.81(11)
C6a	6443.7(4)	2674.8(9)	7883.9(4)	19.13(12)
C7a	7501.2(4)	2629.0(8)	7708.0(3)	16.11(11)

Table 2 Fractional Atomic Coordinates (×10[4]) and Equivalent Isotropic Displacement Parameters (Å[2]×10[3]) for DL-08-62-08. U_{eq} is defined as 1/3 of the trace of the orthogonalised U_{IJ} tensor.

Atom	*x*	*y*	*z*	U(eq)
C8a	7135.7(4)	1395.9(8)	6834.8(4)	18.11(11)
C9a	7002.1(4)	6188.2(8)	7471.6(4)	17.65(12)
C10a	5486.1(4)	3187.5(8)	6136.4(4)	19.30(12)
C11a	4801.7(5)	3687.3(10)	4567.3(5)	29.19(14)
C12a	8369.4(4)	2317.4(7)	8418.8(3)	16.04(11)
C13a	9226.9(4)	1718.8(8)	8148.4(4)	18.34(11)
C14a	10043.1(4)	1444.3(8)	8815.1(4)	20.91(12)
C15a	10008.1(4)	1767.5(8)	9759.8(4)	22.22(12)
C16a	9155.6(4)	2344.9(8)	10035.3(4)	21.96(12)
C17a	8339.4(4)	2618.7(8)	9368.4(4)	19.63(12)

Table 3 Anisotropic Displacement Parameters (Å[2]×10[3]) for DL-08-62-08. The Anisotropic displacement factor exponent takes the form: $-2\pi^2[h^2a^{*2}U_{11}+2hka^*b^*U_{12}+\ldots]$.

Atom	U_{11}	U_{22}	U_{33}	U_{12}	U_{13}	U_{23}
O1	25.3(2)	25.4(2)	22.4(2)	0.30(19)	3.58(16)	-6.59(18)
O2	20.3(2)	30.6(2)	20.14(18)	8.75(19)	1.75(15)	-0.71(18)
O3	17.16(19)	23.6(2)	15.64(19)	-1.42(17)	-0.66(14)	2.10(16)
O4	23.8(2)	43.2(3)	17.37(19)	-4.8(2)	2.63(16)	11.47(19)
C1	24.3(3)	20.8(3)	19.9(3)	-2.7(3)	-1.0(2)	-0.1(3)
C2	20.6(3)	23.7(3)	22.6(3)	-5.3(3)	0.6(2)	2.9(3)
C3	20.7(3)	24.6(3)	19.9(3)	-5.2(3)	6.5(2)	0.7(3)
C4	13.5(2)	18.8(3)	13.6(2)	0.0(2)	2.54(19)	1.5(2)
C5	13.5(2)	19.7(3)	12.8(2)	0.0(2)	1.78(19)	1.8(2)
C6	14.2(3)	25.1(3)	14.2(3)	-1.8(3)	1.3(2)	4.0(2)
C7	14.0(3)	19.1(3)	14.0(2)	0.0(2)	1.24(19)	2.9(2)
C8	16.1(3)	19.6(3)	16.0(3)	1.6(2)	1.1(2)	0.4(2)

Table 3 Anisotropic Displacement Parameters (Å^2×10^3) for DL-08-62-08. The Anisotropic displacement factor exponent takes the form: -2π^2[h^{2}a*^{2}U$_{11}$+2hka*b*U$_{12}$+…].

Atom	U$_{11}$	U$_{22}$	U$_{33}$	U$_{12}$	U$_{13}$	U$_{23}$
C9	18.8(3)	20.8(3)	15.3(2)	3.0(2)	3.0(2)	1.4(2)
C10	16.7(3)	19.7(3)	12.9(3)	0.9(2)	2.4(2)	1.5(2)
C11	25.1(3)	23.3(3)	18.0(3)	1.1(3)	-5.3(3)	-0.5(3)
C12	15.1(3)	18.3(3)	14.2(2)	-0.9(2)	0.40(19)	2.5(2)
C13	15.8(3)	23.1(3)	15.5(3)	0.8(2)	-0.1(2)	1.3(2)
C14	17.1(3)	29.5(3)	18.3(3)	0.3(3)	-2.4(2)	2.9(2)
C15	21.5(3)	32.1(3)	16.6(3)	-3.0(3)	-2.4(2)	4.9(3)
C16	23.5(3)	37.9(4)	14.9(3)	-1.1(3)	2.1(2)	6.9(2)
C17	17.8(3)	30.7(3)	15.9(3)	-0.1(3)	2.8(2)	5.2(2)
H1a	40(5)	25(5)	34(4)	-1(4)	4(4)	7(4)
H1b	38(5)	25(5)	38(5)	-5(4)	-4(4)	0(4)
H2a	71(6)	30(5)	25(4)	-15(5)	5(4)	4(4)
H2b	20(4)	71(7)	45(5)	-1(5)	0(4)	-6(5)
H3a	47(5)	74(7)	29(4)	-29(5)	6(4)	14(5)
H3b	25(5)	32(5)	82(7)	-6(4)	24(5)	-11(5)
H6a	32(4)	26(5)	28(4)	-21(4)	4(3)	1(4)
H6b	24(4)	37(5)	23(4)	-1(4)	8(3)	12(4)
H8a	33(4)	25(5)	24(4)	6(4)	-3(3)	-2(3)
H8b	15(4)	43(5)	32(4)	5(4)	6(3)	-5(4)
H11a	54(6)	43(6)	38(5)	-7(5)	-18(4)	0(5)
H11b	73(6)	68(7)	23(4)	20(5)	-1(4)	-17(5)
H11c	26(5)	70(7)	45(5)	-20(5)	-11(4)	13(5)
H13	25(4)	47(5)	18(4)	7(4)	2(3)	1(4)
H14	23(4)	56(6)	30(4)	6(4)	-6(3)	5(4)
H15	38(4)	64(6)	19(4)	0(5)	-8(3)	13(4)

Table 3 Anisotropic Displacement Parameters (Å^2×10^3) for DL-08-62-08. The Anisotropic displacement factor exponent takes the form: $-2\pi^2[h^2a^{*2}U_{11}+2hka^*b^*U_{12}+\ldots]$.

Atom	U_{11}	U_{22}	U_{33}	U_{12}	U_{13}	U_{23}
H16	37(4)	89(7)	20(4)	-2(5)	11(3)	8(5)
H17	16(4)	67(6)	38(4)	3(4)	12(3)	11(4)
O1a	25.8(2)	17.8(2)	21.30(18)	0.02(18)	6.19(15)	-5.01(17)
O2a	22.3(2)	23.8(2)	23.65(19)	8.07(18)	5.39(15)	-3.14(17)
O3a	22.4(2)	26.5(2)	19.15(18)	0.47(18)	0.13(15)	1.86(17)
O4a	14.93(19)	56.9(3)	25.9(2)	3.3(2)	3.34(16)	0.6(2)
C1a	21.7(3)	17.8(3)	25.7(3)	-1.1(3)	2.5(2)	-2.5(3)
C2a	25.3(3)	21.9(3)	25.9(3)	-2.8(3)	10.8(2)	-1.8(3)
C3a	22.1(3)	20.3(3)	16.1(3)	-0.7(3)	6.5(2)	-1.1(2)
C4a	16.5(3)	15.4(3)	14.4(2)	2.1(2)	4.1(2)	-0.9(2)
C5a	14.9(2)	18.8(3)	16.9(2)	1.6(2)	3.1(2)	-0.5(2)
C6a	16.5(3)	23.4(3)	18.1(3)	0.0(3)	4.8(2)	2.3(2)
C7a	15.3(2)	16.9(3)	16.5(2)	1.4(2)	3.67(19)	0.7(2)
C8a	17.8(3)	15.8(3)	20.4(3)	0.9(2)	2.1(2)	-1.0(2)
C9a	20.4(3)	17.3(3)	15.7(2)	4.2(2)	4.7(2)	-0.5(2)
C10a	16.5(3)	21.7(3)	19.3(3)	3.3(2)	1.8(2)	-2.0(2)
C11a	32.3(4)	28.6(4)	23.3(3)	3.6(3)	-5.8(3)	1.3(3)
C12a	16.2(2)	15.9(3)	16.1(2)	0.0(2)	3.0(2)	0.9(2)
C13a	17.3(3)	20.2(3)	17.7(3)	3.6(2)	3.4(2)	2.0(2)
C14a	17.9(3)	23.2(3)	21.5(3)	3.0(3)	2.5(2)	3.4(2)
C15a	20.9(3)	24.4(3)	19.9(3)	0.7(3)	-1.2(2)	2.5(2)
C16a	24.6(3)	24.5(3)	16.2(3)	-0.6(3)	1.4(2)	0.2(2)
C17a	19.6(3)	22.8(3)	16.9(3)	1.1(3)	4.3(2)	-0.1(2)
H1aa	34(5)	24(5)	47(5)	9(4)	-1(4)	-4(4)
H1ab	32(4)	24(4)	37(4)	-10(4)	6(3)	-3(4)

Table 3 Anisotropic Displacement Parameters (Å^2×10^3) for DL-08-62-08. The Anisotropic displacement factor exponent takes the form: -2π^2[h^{2}a*^{2}U$_{11}$+2hka*b*U$_{12}$+…].

Atom	U$_{11}$	U$_{22}$	U$_{33}$	U$_{12}$	U$_{13}$	U$_{23}$
H2aa	54(5)	39(6)	37(5)	14(5)	14(4)	2(4)
H2ab	29(5)	60(6)	64(6)	-11(4)	28(4)	-26(5)
H3aa	38(5)	63(6)	28(5)	-10(5)	6(4)	2(4)
H3ab	57(6)	29(5)	56(5)	10(5)	38(4)	-3(4)
H6aa	31(5)	36(5)	30(4)	-7(4)	5(4)	6(4)
H6ab	31(5)	32(5)	33(4)	8(4)	6(3)	6(4)
H8aa	39(5)	28(5)	36(5)	2(4)	5(4)	-2(4)
H8ab	25(4)	32(5)	35(4)	4(4)	11(4)	-3(4)
H11d	51(6)	65(7)	33(5)	3(5)	-2(4)	7(5)
H11e	85(7)	35(6)	56(6)	-22(6)	-33(5)	6(5)
H11f	41(5)	64(6)	35(5)	17(5)	-1(4)	-1(5)
H13a	33(5)	47(6)	23(4)	12(4)	5(3)	5(4)
H14a	22(4)	47(5)	39(5)	11(4)	5(4)	8(4)
H15a	39(5)	70(7)	25(4)	9(5)	-8(4)	-7(4)
H16a	37(4)	54(6)	28(4)	2(4)	7(3)	-7(4)
H17a	33(4)	56(6)	27(4)	6(4)	8(3)	2(4)

Table 4 Bond Lengths for DL-08-62-08.

Atom	Atom	Length/Å	Atom	Atom	Length/Å
O1	C1	1.4500(7)	O1a	C1a	1.4496(7)
O1	C9	1.3388(7)	O1a	C9a	1.3433(7)
O2	C9	1.2106(6)	O2a	C9a	1.2104(7)
O3	C10	1.3308(7)	O3a	C10a	1.3368(6)
O3	C11	1.4422(6)	O3a	C11a	1.4433(7)
O4	C10	1.2055(7)	O4a	C10a	1.2028(7)

Table 4 Bond Lengths for DL-08-62-08.

Atom	Atom	Length/Å	Atom	Atom	Length/Å
C1	C2	1.5146(8)	C1a	C2a	1.5118(8)
C2	C3	1.5392(8)	C2a	C3a	1.5381(8)
C3	C4	1.5405(7)	C3a	C4a	1.5402(7)
C4	C5	1.5583(7)	C4a	C5a	1.5565(8)
C4	C7	1.6106(7)	C4a	C7a	1.6148(7)
C4	C9	1.5200(7)	C4a	C9a	1.5158(7)
C5	C6	1.5537(7)	C5a	C6a	1.5508(7)
C5	C7	1.8846(7)	C5a	C7a	1.8827(7)
C5	C8	1.5590(8)	C5a	C8a	1.5603(8)
C5	C10	1.4899(7)	C5a	C10a	1.4885(7)
C6	C7	1.5574(7)	C6a	C7a	1.5537(7)
C7	C8	1.5611(7)	C7a	C8a	1.5631(8)
C7	C12	1.4838(7)	C7a	C12a	1.4856(7)
C12	C13	1.3986(7)	C12a	C13a	1.3980(7)
C12	C17	1.3952(7)	C12a	C17a	1.3980(7)
C13	C14	1.3954(8)	C13a	C14a	1.3934(8)
C14	C15	1.3938(8)	C14a	C15a	1.3950(8)
C15	C16	1.3914(8)	C15a	C16a	1.3899(8)
C16	C17	1.3929(8)	C16a	C17a	1.3935(8)

Table 5 Bond Angles for DL-08-62-08.

Atom	Atom	Atom	Angle/˚	Atom	Atom	Atom	Angle/˚
C9	O1	C1	117.06(4)	C9a	O1a	C1a	116.83(4)
C11	O3	C10	116.16(4)	C11a	O3a	C10a	115.25(5)
C2	C1	O1	108.86(5)	C2a	C1a	O1a	108.20(5)
C3	C2	C1	111.01(5)	C3a	C2a	C1a	110.46(5)

Table 5 Bond Angles for DL-08-62-08.

Atom	Atom	Atom	Angle/˚	Atom	Atom	Atom	Angle/˚
C4	C3	C2	112.19(5)	C4a	C3a	C2a	111.87(5)
C5	C4	C3	120.86(4)	C5a	C4a	C3a	121.38(4)
C7	C4	C3	118.84(5)	C7a	C4a	C3a	119.16(4)
C7	C4	C5	72.96(4)	C7a	C4a	C5a	72.81(4)
C9	C4	C3	109.62(5)	C9a	C4a	C3a	108.91(4)
C9	C4	C5	114.07(4)	C9a	C4a	C5a	114.20(4)
C9	C4	C7	116.59(4)	C9a	C4a	C7a	116.79(4)
C6	C5	C4	89.71(4)	C6a	C5a	C4a	89.74(4)
C7	C5	C4	54.80(3)	C7a	C5a	C4a	55.02(3)
C7	C5	C6	52.80(3)	C7a	C5a	C6a	52.74(3)
C8	C5	C4	88.37(4)	C8a	C5a	C4a	88.44(4)
C8	C5	C6	86.65(4)	C8a	C5a	C6a	86.91(4)
C8	C5	C7	52.89(3)	C8a	C5a	C7a	53.00(3)
C10	C5	C4	125.46(5)	C10a	C5a	C4a	128.56(5)
C10	C5	C6	129.73(5)	C10a	C5a	C6a	124.91(4)
C10	C5	C7	176.77(4)	C10a	C5a	C7a	176.38(5)
C10	C5	C8	124.14(4)	C10a	C5a	C8a	125.61(5)
C7	C6	C5	74.57(4)	C7a	C6a	C5a	74.67(4)
C5	C7	C4	52.24(3)	C5a	C7a	C4a	52.17(3)
C6	C7	C4	87.69(4)	C6a	C7a	C4a	87.54(4)
C6	C7	C5	52.63(3)	C6a	C7a	C5a	52.60(3)
C8	C7	C4	86.46(4)	C8a	C7a	C4a	86.31(4)
C8	C7	C5	52.79(3)	C8a	C7a	C5a	52.86(3)
C8	C7	C6	86.45(4)	C8a	C7a	C6a	86.72(4)
C12	C7	C4	127.04(5)	C12a	C7a	C4a	128.60(5)
C12	C7	C5	179.27(5)	C12a	C7a	C5a	179.15(4)

Table 5 Bond Angles for DL-08-62-08.

Atom	Atom	Atom	Angle/˚	Atom	Atom	Atom	Angle/˚
C12	C7	C6	127.64(4)	C12a	C7a	C6a	126.69(4)
C12	C7	C8	127.67(5)	C12a	C7a	C8a	127.08(5)
C7	C8	C5	74.32(4)	C7a	C8a	C5a	74.14(4)
O2	C9	O1	118.79(5)	O2a	C9a	O1a	119.18(5)
C4	C9	O1	116.85(4)	C4a	C9a	O1a	116.28(5)
C4	C9	O2	124.35(5)	C4a	C9a	O2a	124.51(5)
O4	C10	O3	123.79(5)	O4a	C10a	O3a	123.35(5)
C5	C10	O3	112.35(4)	C5a	C10a	O3a	112.59(4)
C5	C10	O4	123.83(5)	C5a	C10a	O4a	123.99(5)
C13	C12	C7	120.62(5)	C13a	C12a	C7a	120.53(4)
C17	C12	C7	119.94(5)	C17a	C12a	C7a	120.38(5)
C17	C12	C13	119.41(5)	C17a	C12a	C13a	119.09(5)
C14	C13	C12	120.04(5)	C14a	C13a	C12a	120.45(5)
C15	C14	C13	120.20(5)	C15a	C14a	C13a	120.00(6)
C16	C15	C14	119.86(5)	C16a	C15a	C14a	119.89(6)
C17	C16	C15	120.03(5)	C17a	C16a	C15a	120.10(5)
C16	C17	C12	120.46(5)	C16a	C17a	C12a	120.46(5)

Table 6 Torsion Angles for DL-08-62-08.

A	B	C	D	Angle/˚	A	B	C	D	Angle/˚
O1	C1	C2	C3	-59.63(6)	O1a	C1a	C2a	C3a	-62.59(5)
O1	C9	C4	C3	-52.04(5)	O1a	C9a	C4a	C3a	-55.70(5)
O1	C9	C4	C5	168.87(4)	O1a	C9a	C4a	C5a	165.07(4)
O1	C9	C4	C7	86.65(5)	O1a	C9a	C4a	C7a	82.89(5)
O2	C9	O1	C1	-171.25(5)	O2a	C9a	O1a	C1a	-164.61(5)
O2	C9	C4	C3	126.80(6)	O2a	C9a	C4a	C3a	122.53(6)

Table 6 Torsion Angles for DL-08-62-08.

A	B	C	D	Angle/˚	A	B	C	D	Angle/˚
O2	C9	C4	C5	-12.29(6)	O2a	C9a	C4a	C5a	-16.71(6)
O2	C9	C4	C7	-94.51(6)	O2a	C9a	C4a	C7a	-98.88(5)
O3	C10	C5	C4	-134.29(4)	O3a	C10a	C5a	C4a	50.24(6)
O3	C10	C5	C6	-8.57(6)	O3a	C10a	C5a	C6a	173.57(5)
O3	C10	C5	C8	109.57(5)	O3a	C10a	C5a	C8a	-71.65(6)
O4	C10	O3	C11	2.66(7)	O4a	C10a	O3a	C11a	-2.44(7)
O4	C10	C5	C4	47.54(7)	O4a	C10a	C5a	C4a	-132.68(6)
O4	C10	C5	C6	173.25(6)	O4a	C10a	C5a	C6a	-9.35(8)
O4	C10	C5	C8	-68.61(7)	O4a	C10a	C5a	C8a	105.42(6)
C1	O1	C9	C4	7.66(5)	C1a	O1a	C9a	C4a	13.72(5)
C1	C2	C3	C4	15.54(6)	C1a	C2a	C3a	C4a	20.46(6)
C2	C1	O1	C9	48.95(6)	C2a	C1a	O1a	C9a	45.85(6)
C2	C3	C4	C5	172.29(5)	C2a	C3a	C4a	C5a	169.99(5)
C2	C3	C4	C7	-101.19(5)	C2a	C3a	C4a	C7a	-103.22(5)
C2	C3	C4	C9	36.44(6)	C2a	C3a	C4a	C9a	34.23(6)
C3	C4	C5	C6	156.45(6)	C3a	C4a	C5a	C6a	156.76(6)
C3	C4	C5	C7	113.86(7)	C3a	C4a	C5a	C7a	114.13(7)
C3	C4	C5	C8	69.80(5)	C3a	C4a	C5a	C8a	69.84(5)
C3	C4	C5	C10	-62.19(6)	C3a	C4a	C5a	C10a	-66.49(6)
C3	C4	C7	C5	-116.34(6)	C3a	C4a	C7a	C5a	-116.84(6)
C3	C4	C7	C6	-158.84(5)	C3a	C4a	C7a	C6a	-159.42(6)
C3	C4	C7	C8	-72.26(5)	C3a	C4a	C7a	C8a	-72.56(5)
C3	C4	C7	C12	63.83(5)	C3a	C4a	C7a	C12a	63.62(6)
C4	C5	C6	C7	-43.96(5)	C4a	C5a	C6a	C7a	-44.20(5)
C4	C5	C7	C6	121.84(6)	C4a	C5a	C7a	C6a	121.69(6)
C4	C5	C7	C8	-119.34(5)	C4a	C5a	C7a	C8a	-119.06(5)

Table 6 Torsion Angles for DL-08-62-08.

A	B	C	D	Angle/˚	A	B	C	D	Angle/˚
C4	C5	C8	C7	45.45(4)	C4a	C5a	C8a	C7a	45.76(4)
C4	C7	C5	C6	-121.84(6)	C4a	C7a	C5a	C6a	-121.69(6)
C4	C7	C5	C8	119.34(5)	C4a	C7a	C5a	C8a	119.06(5)
C4	C7	C6	C5	42.24(5)	C4a	C7a	C6a	C5a	42.27(5)
C4	C7	C8	C5	-43.67(5)	C4a	C7a	C8a	C5a	-43.77(4)
C4	C7	C12	C13	-83.24(6)	C4a	C7a	C12a	C13a	-79.04(6)
C4	C7	C12	C17	94.79(6)	C4a	C7a	C12a	C17a	100.99(5)
C5	C4	C7	C6	-42.51(4)	C5a	C4a	C7a	C6a	-42.58(4)
C5	C4	C7	C8	44.07(4)	C5a	C4a	C7a	C8a	44.29(4)
C5	C4	C7	C12	-179.84(3)	C5a	C4a	C7a	C12a	-179.54(3)
C5	C6	C7	C8	-44.36(4)	C5a	C6a	C7a	C8a	-44.17(4)
C5	C6	C7	C12	179.14(3)	C5a	C6a	C7a	C12a	-179.43(3)
C5	C7	C4	C9	108.88(3)	C5a	C7a	C4a	C9a	108.93(4)
C5	C8	C7	C6	44.23(4)	C5a	C8a	C7a	C6a	43.97(4)
C5	C8	C7	C12	-179.29(3)	C5a	C8a	C7a	C12a	178.94(3)
C5	C10	O3	C11	-175.52(5)	C5a	C10a	O3a	C11a	174.66(5)
C6	C5	C4	C7	42.59(4)	C6a	C5a	C4a	C7a	42.63(4)
C6	C5	C4	C9	-69.49(4)	C6a	C5a	C4a	C9a	-69.59(4)
C6	C5	C7	C8	118.82(6)	C6a	C5a	C7a	C8a	119.25(6)
C6	C5	C8	C7	-44.36(5)	C6a	C5a	C8a	C7a	-44.06(5)
C6	C7	C4	C9	66.38(4)	C6a	C7a	C4a	C9a	66.35(4)
C6	C7	C5	C8	-118.82(6)	C6a	C7a	C5a	C8a	-119.25(6)
C6	C7	C12	C13	155.54(6)	C6a	C7a	C12a	C13a	159.21(6)
C6	C7	C12	C17	-26.43(7)	C6a	C7a	C12a	C17a	-20.76(7)
C7	C4	C5	C8	-44.07(4)	C7a	C4a	C5a	C8a	-44.29(4)
C7	C4	C5	C10	-176.05(3)	C7a	C4a	C5a	C10a	179.38(3)

Table 6 Torsion Angles for DL-08-62-08.

A	B	C	D	Angle/˚	A	B	C	D	Angle/˚
C7	C5	C4	C9	-112.08(3)	C7a	C5a	C4a	C9a	-112.22(3)
C7	C6	C5	C8	44.42(4)	C7a	C6a	C5a	C8a	44.25(4)
C7	C6	C5	C10	177.44(3)	C7a	C6a	C5a	C10a	176.59(4)
C7	C8	C5	C10	178.44(3)	C7a	C8a	C5a	C10a	-175.85(3)
C7	C12	C13	C14	178.49(5)	C7a	C12a	C13a	C14a	179.39(5)
C7	C12	C17	C16	-178.09(6)	C7a	C12a	C17a	C16a	-179.36(5)
C8	C5	C4	C9	-156.15(3)	C8a	C5a	C4a	C9a	-156.51(3)
C8	C7	C4	C9	152.96(3)	C8a	C7a	C4a	C9a	153.22(4)
C8	C7	C12	C13	35.76(6)	C8a	C7a	C12a	C13a	40.95(6)
C8	C7	C12	C17	-146.20(6)	C8a	C7a	C12a	C17a	-139.02(6)
C9	C4	C5	C10	71.87(5)	C9a	C4a	C5a	C10a	67.16(5)
C9	C4	C7	C12	-70.95(5)	C9a	C4a	C7a	C12a	-70.60(5)
C12	C13	C14	C15	-0.12(7)	C12a	C13a	C14a	C15a	-0.02(7)
C12	C17	C16	C15	-0.70(7)	C12a	C17a	C16a	C15a	-0.03(7)
C13	C12	C17	C16	-0.04(7)	C13a	C12a	C17a	C16a	0.67(6)
C13	C14	C15	C16	-0.62(7)	C13a	C14a	C15a	C16a	0.66(7)
C14	C13	C12	C17	0.44(7)	C14a	C13a	C12a	C17a	-0.65(7)
C14	C15	C16	C17	1.02(7)	C14a	C15a	C16a	C17a	-0.64(7)

Table 7 Hydrogen Atom Coordinates (Å×10^4) and Isotropic Displacement Parameters (Å^2×10^3) for DL-08-62-08.

Atom	*x*	*y*	*z*	U(eq)
H1a	3548(5)	6947(11)	8788(5)	32.9(18)
H1b	3654(5)	9410(10)	8940(5)	34.8(19)
H2a	3621(6)	9849(12)	7261(5)	43(2)
H2b	4542(5)	8264(13)	7657(5)	46(2)

Table 7 Hydrogen Atom Coordinates (Å×10[4]) and Isotropic Displacement Parameters (Å[2]×10[3]) for DL-08-62-08.

Atom	*x*	*y*	*z*	U(eq)
H3a	3077(5)	7590(13)	6136(5)	50(2)
H3b	3809(5)	5891(12)	6760(6)	45(2)
H6a	1078(5)	2546(11)	7246(4)	28.8(17)
H6b	882(5)	4818(11)	7701(5)	27.7(17)
H8a	2270(5)	1896(10)	6531(4)	27.9(17)
H8b	3171(4)	3606(11)	6340(4)	29.6(18)
H11a	-281.8(5)	6394(6)	4351.5(8)	48(2)
H11b	-15.6(13)	4302(3)	4033(2)	56(3)
H11c	-974(3)	4640(2)	4517.3(4)	49(2)
H13	4337(4)	3162(11)	7946(4)	30.2(18)
H14	5318(5)	2347(12)	9490(4)	38(2)
H15	4565(5)	2357(12)	10926(4)	42(2)
H16	2854(5)	3208(14)	10830(4)	48(2)
H17	1886(5)	3971(12)	9304(5)	40(2)
H1aa	8945(5)	5505(11)	8009(6)	36(2)
H1ab	9143(5)	7943(11)	7855(5)	31.2(18)
H2aa	8257(5)	7764(12)	6225(5)	42(2)
H2ab	9263(5)	6219(13)	6381(6)	49(2)
H3aa	7423(6)	5119(13)	5617(5)	43(2)
H3ab	8302(5)	3608(11)	6174(5)	45(2)
H6aa	6167(5)	1336(11)	8073(5)	32.4(19)
H6ab	6224(5)	3816(11)	8305(5)	32.1(18)
H8aa	6866(5)	47(11)	7010(5)	34(2)
H8ab	7558(5)	1361(10)	6271(5)	29.7(18)
H11d	4988.9(9)	4133(2)	3944(3)	50(2)

Table 7 Hydrogen Atom Coordinates (Å×10^4) and Isotropic Displacement Parameters (Å^2×10^3) for DL-08-62-08.

Atom	*x*	*y*	*z*	U(eq)
H11e	4512.0(13)	2374(6)	4481.9(6)	64(3)
H11f	4303(2)	4583(4)	4767.8(9)	48(2)
H13a	9257.3(4)	1465.0(17)	7411(4)	34(2)
H14a	10703(4)	981(3)	8600.5(15)	36(2)
H15a	10641(4)	1569.9(15)	10275(3)	46(2)
H16a	9125.9(4)	2580.9(17)	10765(4)	40(2)
H17a	7683(4)	3064(3)	9585.2(14)	38(2)

Experimental

Single crystals of $C_{17}H_{18}O_4$ **[DL-08-62-08]** were **[The material was recrystallised from hexane by as supplied]**. A suitable crystal was selected and **[The crystal was mounted on a loop with paratone]** on a **XtaLAB Synergy, Dualflex, HyPix** diffractometer. The crystal was kept at 100.01(10) K during data collection. Using Olex2 [1], the structure was solved with the SHELXT [2] structure solution program using Intrinsic Phasing and refined with the olex2.refine [3] refinement package using Gauss-Newton minimisation.

1. Dolomanov, O.V., Bourhis, L.J., Gildea, R.J, Howard, J.A.K. & Puschmann, H. (2009), J. Appl. Cryst. 42, 339-341.
2. Sheldrick, G.M. (2015). Acta Cryst. A71, 3-8.
3. Bourhis, L.J., Dolomanov, O.V., Gildea, R.J., Howard, J.A.K., Puschmann, H. (2015). Acta Cryst. A71, 59-75.

Crystal structure determination of [DL-08-62-08]

Crystal Data for $C_{17}H_{18}O_4$ (M =286.330 g/mol): monoclinic, space group $P2_1$ (no. 4), a = 14.11139(11) Å, b = 7.20455(5) Å, c = 14.46648(10) Å, β = 99.1803(7)°, V = 1451.913(19) Å^3, Z = 4, T = 100.01(10) K, μ(Cu Kα) = 0.761 mm^{-1}, $Dcalc$ = 1.310 g/cm^3, 23859 reflections measured (6.18° ≤ 2Θ ≤ 154.18°), 5609 unique (R_{int} = 0.0184, R_{sigma} = 0.0145) which were used in all calculations. The final R_1 was 0.0112 (I>=2u(I)) and wR_2 was 0.0236 (all data).

Refinement model description

Number of restraints - 1, number of constraints - 7.

Details:

1. Twinned data refinement
 Scales: 0.51(4)
 0.49(4)
2.a Aromatic/amide H refined with riding coordinates and stretchable bonds:
 C13a(H13a), C17a(H17a), C16a(H16a), C15a(H15a), C14a(H14a)

2.b Idealised Me refined with riding coordinates and stretchable bonds:
C11a(H11d,H11e,H11f), C11(H11a,H11b,H11c)

10. References

1. Sharland, J. C.; Davies, H. M. L., One-Pot Synthesis of Difluorobicyclo[1.1.1]pentanes from α-Allyldiazoacetates. *Org. Lett.* **2023,** *25* (28), 5214-5219.
2. Keipour, H.; Ollevier, T., Iron-Catalyzed Carbene Insertion Reactions of α-Diazoesters into Si–H Bonds. *Org. Lett.* **2017,** *19* (21), 5736-5739.
3. Toma, T.; Shimokawa, J.; Fukuyama, T., N,N'-Ditosylhydrazine: A Convenient Reagent for Facile Synthesis of Diazoacetates. *Org. Lett.* **2007,** *9* (16), 3195-3197.
4. Fu, L.; Mighion, J. D.; Voight, E. A.; Davies, H. M. L., Synthesis of 2,2,2,-Trichloroethyl Aryl- and Vinyldiazoacetates by Palladium-Catalyzed Cross-Coupling. *Chem. Eur. J.* **2017,** *23* (14), 3272-3275.
5. Chanthamath, S.; Ozaki, S.; Shibatomi, K.; Iwasa, S., Highly Stereoselective Synthesis of Cyclopropylphosphonates Catalyzed by Chiral Ru(II)-Pheox Complex. *Org. Lett.* **2014,** *16* (11), 3012-3015.
6. Le, T. V.; Romero, I.; Daugulis, O., “Sandwich” Diimine-Copper Catalyzed Trifluoroethylation and Pentafluoropropylation of Unactivated C(sp3)−H Bonds by Carbene Insertion. *Chem. Eur. J.* **2023,** *29* (48), e202301672.
7. Chen, K.; Zhang, S.-Q.; Brandenberg, O. F.; Hong, X.; Arnold, F. H., Alternate Heme Ligation Steers Activity and Selectivity in Engineered Cytochrome P450-Catalyzed Carbene-Transfer Reactions. *J. Am. Chem. Soc.* **2018,** *140* (48), 16402-16407.
8. Chen, K.; Huang, X.; Zhang, S.-Q.; Zhou, A. Z.; Kan, S. B. J.; Hong, X.; Arnold, F. H., Engineered Cytochrome c-Catalyzed Lactone-Carbene B–H Insertion. *Synlett* **2019,** *30* (04), 378-382.
9. O'Brien, J. G. K.; Conway, L. P.; Ramaraj, P. K.; Jadhav, A. M.; Jin, J.; Dutra, J. K.; Evers, P.; Masoud, S. S.; Schupp, M.; Saridakis, I.; Chen, Y.; Maulide, N.; Pezacki, J. P.; am Ende, C. W.; Parker, C. G.; Fox, J. M., Mechanistic differences between linear vs. spirocyclic dialkyldiazirine probes for photoaffinity labeling. *Chem. Sci.* **2024,** *15* (37), 15463-15473.
